# Ion‐Driven Interfacial Engineering of MXene–Polyacrylamide Hydrogels for Advanced Wearable Electrocardiography and AI‐Driven Blood Pressure Monitoring

**DOI:** 10.1002/smsc.202500526

**Published:** 2026-03-28

**Authors:** Bangul Khan, Bilawal Khan, Syed Bilal Ahmed, Wasim Ullah Khan, Junchen Liao, Md. Shohidul Islam, Iyappan Gunasekaran, Rafi U Shan Ahmad, Mohamed Elhousseini Hilal, Bee Luan Khoo

**Affiliations:** ^1^ Department of Biomedical Engineering College of Biomedicine City University of Hong Kong Kowloon Tong 999077 Hong Kong; ^2^ Hong Kong Centre of Cerebro‐Cardiovascular Health Engineering (COCHE) Shatin 999077 Hong Kong; ^3^ Department of Materials Science and Engineering City University of Hong Kong Kowloon Tong 999077 Hong Kong; ^4^ School of Information Engineering Yango University Fuzhou Fujian 350015 China; ^5^ Institute of Digital Medicine City University of Hong Kong Kowloon Tong 999077 Hong Kong

**Keywords:** artificial intelligence, blood pressure monitoring, conductive hydrogel, MXene, Ti_3_C_2_T_*x*_, wearable electrocardiography

## Abstract

MXene (Ti_3_C_2_T_*x*_)‐based hydrogels hold great promise for wearable bioelectronics but are limited by unstable interlayer spacing, poor mechanical resilience, and inadequate skin compatibility. An ion‐driven interfacial engineering strategy is introduced that stabilizes Ti_3_C_2_T_*x*_ nanosheets via Ca^2+^ intercalation and Cl^−^ electrostatic screening, expanding interlayer spacing and enabling ultrafast gelation (<5 min). The resulting Ti_3_C_2_T_*x*_−polyacrylamide hydrogel exhibits ultrastretchability (2920%), high conductivity (0.39 s m^−1^), and ≈99% cell viability, surpassing existing MXene and commercial hydrogels in terms of cytocompatibility, durability, and skin adherence. Molecular dynamics simulations reveal dynamic interlayer adaptability (6.2–8.2 Å) and ion transport mechanisms that underpin strain‐adaptive sensing and stability across a temperature range of –20 to 40 °C. Integrated into wearable electrodes, the hydrogel enables high‐quality electrocardiography (ECG) acquisition across diverse skin types, achieving signal‐to‐noise ratios of up to 27.2 dB without the need for auxiliary treatments. Coupled with a convolutional neural network–bidirectional gated recurrent unit model trained on ECG data from 17 subjects, the system delivers real‐time, cuffless blood pressure estimation with MAE ± SD of 2.91 ± 3.03 mmHg (systolic blood pressure) and 2.36 ± 2.33 mmHg (diastolic blood pressure), meeting Association for the Advancement of Medical Instrumentation and British Hypertension Society standards. This synergistic material and AI framework establishes a new paradigm for smart, long‐term cardiovascular monitoring.

## Graphical abstract

Interfacial engineering of Ti_3_C_2_T_X_ nanosheets via Ca^2+^ and Cl^−^ ion intercalation stabilized interlayer spacing within a PAM matrix. Combined with UV‐enhanced crosslinking, this approach enabled rapid gelation of an ultra‐stretchable, conductive Ti_3_C_2_T_X_‐PAM hydrogel. The hydrogel achieved high‐resolution ECG acquisition and AI‐driven blood pressure prediction, offering a promising platform for advanced cardiovascular monitoring.

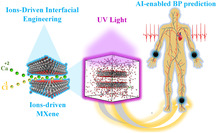

## 1 Introduction

The emergence of wearable bioelectronics has transformed cardiovascular health by facilitating continuous, noninvasive monitoring of physiological parameters. These technologies, particularly wearable interfaces that capture high‐fidelity signals under dynamic mechanical conditions, are essential for cardiovascular monitoring, personalized health tracking, and early disease detection.^[^ ^1^ ^]^ However, Ti_3_C_2_T_*x*_−based hydrogels used in wearable devices often suffer from limitations, including poor mechanical flexibility, low durability, and suboptimal signal quality under strain, which hinders their widespread adoption in real‐life scenarios.^[^ ^2^ ^]^

Cardiovascular diseases represent 31% of global fatalities, serving as a major contributor to worldwide mortality. Each year, these diseases result in around 17 million deaths, highlighting the urgent need for practical preventive, diagnostic, and treatment methods with long‐term physiological monitoring. Nevertheless, conventional wearable devices are bulky and unsuitable for prolonged use due to their unappealing design, material selection, and biocompatibility with the skin.^[^ ^3^, ^4^, ^5^ ^]^

Recent advancements in wearable bioelectronics have yielded a wide array of noninvasive blood pressure (BP) monitoring, facilitating continuous cardiovascular health monitoring. However, these devices suffer from motion artefacts, poor skin‐electrode adhesion, and signal degradation.^[^ ^6^ ^]^ The limited practical utility in everyday life arises from the accuracy and reliability of BP measurements, which are significantly influenced by physical movement, perspiration, and skin characteristics.^[^ ^7^, ^8^, ^9^ ^]^ Flexible electrodes are capable of overcoming these challenges by delivering accurate and reliable readings over prolonged durations. Developing hydrogel electrodes with multiple enhanced properties has garnered considerable research attention.^[^ ^10^ ^]^ Strategies such as incorporating catechol groups, zwitterionic moieties, and oxygen‐containing functional groups have been explored to improve the adhesive performance of hydrogel electrodes.^[^ ^11^ ^]^ Coupling hydrogels with conductive materials, including metallic nanoparticles,^[^ ^12^ ^]^ carbon‐derived nanoparticles,^[^ ^13^ ^]^ ionic liquids,^[^ ^14^ ^]^ and ionic salts,^[^ ^15^ ^]^ enhances their conductivity. Techniques such as constructing dual networks,^[^ ^14^ ^]^ introducing functional monomers,^[^ ^15^ ^]^ and utilizing nanocomposite materials are commonly employed to improve mechanical properties. However, it is essential to note that these high‐performance hydrogels, with controlled strain sensing, improve durability while maintaining excellent transmission performance and wear comfort, which remain significant challenges.^[^ ^16^ ^]^

Currently, 2D conductive MXene (Ti_3_C_2_T_*x*_) nanosheets with theoretical conductivity (>1000 s m^−1^) have emerged as a highly desirable reinforced material to tackle the mentioned challenges.^[^ ^17^ ^]^ The enhanced aspect ratio nanostructures and widespread surface terminations (—OH, —F, —O) of Ti_3_C_2_T_*x*_ promote hydrogen bonding and ionic interactions with various polymers.^[^ ^18^ ^]^ However, during polymer interactions, Ti_3_C_2_T_*x*_ nanosheets form aggregates due to strong interlayer attractions and unstable interlayer spacing,^[^ ^19^ ^]^ which compromises their mechanical stability and prolongs the gelation process.^[^ ^20^ ^]^ Under dynamic conditions, Ti_3_C_2_T_*x*_−based hydrogels gradually lose functionality due to the self‐stacking of Ti_3_C_2_T_*x*_ nanosheets. This occurs due to van der Waals forces and electrostatic interactions between the layers, resulting in aggregation. As a result, the effective dispersion in polymer matrices is reduced, thereby compromising the structural integrity of the hydrogels.^[^ ^21^, ^22^ ^]^

We propose an ion‐driven interfacial engineering strategy to overcome these limitations involving in situ intercalation of Ca^2+^ ions and electrostatic screening by Cl^−^ ions. Ca^2+^ ions coordinate with surface oxygen groups to form molecular pillars, while Cl^−^ ions occupy interstitial voids, mitigating electrostatic interactions and stabilizing interlayer spacing. Molecular dynamics (MD) simulations confirm that this dual‐ion mechanism promotes dynamic interlayer adaptability, enhancing flexibility, mechanical durability, and structural integrity under deformation.

Incorporating ion‐intercalated Ti_3_C_2_T_*x*_ into a polyacrylamide (PAM) matrix yields a conductive hydrogel with rapid gelation (<5 min), ultrahigh stretchability (2920 ± 72.7%), high conductivity (0.39 S m^−^^1^), and strain‐adaptive sensing (gauge factor = 0.1–0.4) across a broad strain range (10–1000%). These properties enable stable, high‐fidelity electrocardiogram (ECG) acquisition (SNR = 26.22 dB) with minimal motion artefacts across diverse skin tones and anatomical sites. Furthermore, integration with a convolutional neural network–bidirectional gated recurrent unit (CNN–BiGRU) deep learning model enables continuous, cuffless BP estimation with MAE ± SD of 2.91 ± 3.03 mmHg (systolic blood pressure [SBP]) and 2.36 ± 2.33 mmHg (diastolic blood pressure [DBP]), outperforming existing approaches. This synergistic material–AI framework offers a robust platform for real‐time cardiovascular monitoring in clinical and nonclinical settings.

## 2 Results and Discussion

### 2.1 In Situ Intercalation of Ti_3_C_2_T_*x*_ Nanosheets

This section details the in situ intercalation of Ca^+2^ and Cl^−^ into Ti_3_C_2_T_*x*_ nanosheets, followed by extensive morphological analysis using scanning electron microscopy (SEM), transmission electron microscopy (TEM), and energy‐dispersive spectroscopy (EDS) to elucidate the visual morphology, homogeneity, surface analysis, and elemental composition of ion‐driven Ti_3_C_2_T_*x*_ nanosheets. The interlayer spacing was calculated using Bragg's law from X‐ray diffraction (XRD) data, followed by chemical and elemental state analysis using X‐ray photoelectron spectroscopy (XPS).

**Figure 1:**
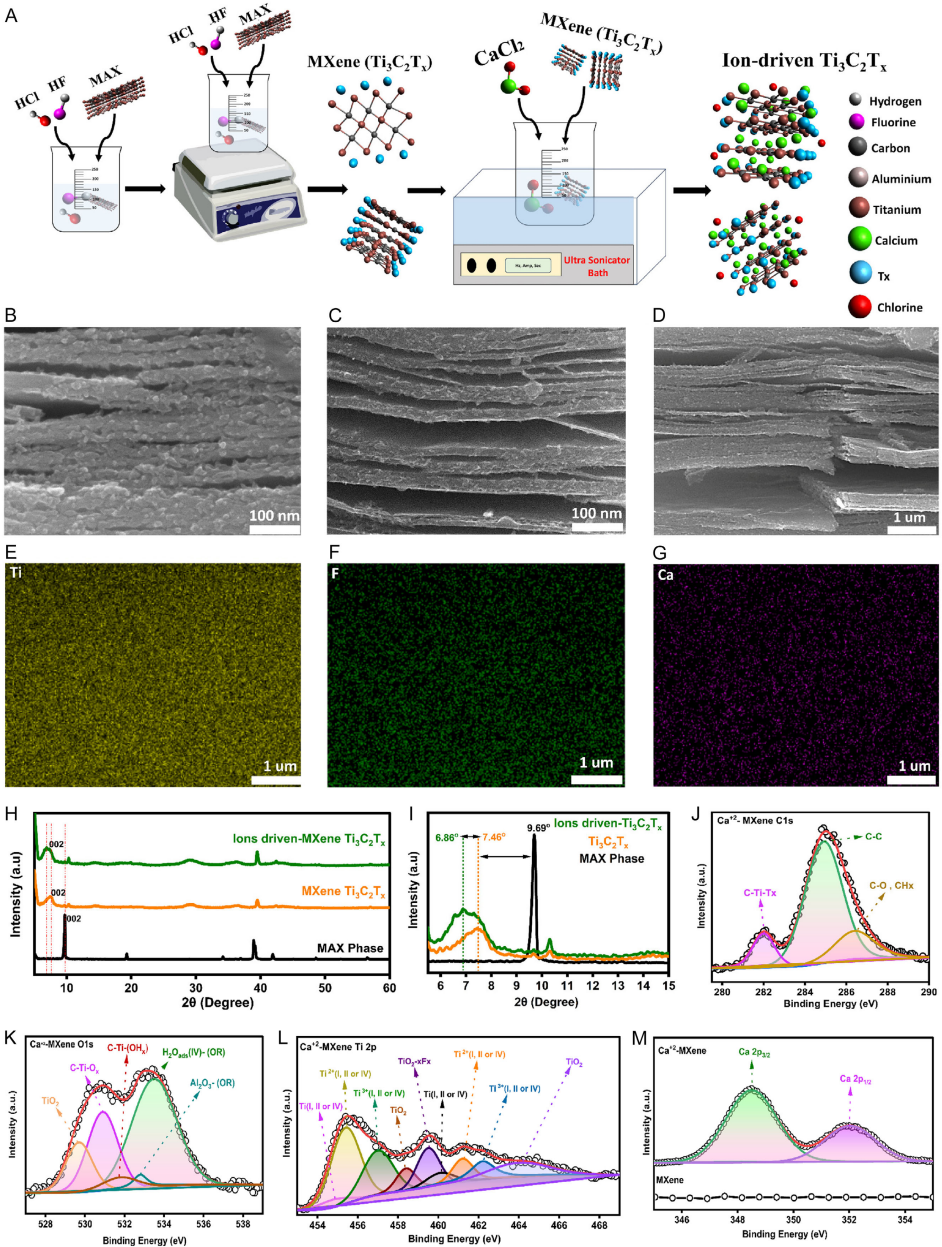
In situ ions Intercalation of pristine Ti_3_C_2_T_*x*_ and characterization of Ti_3_C_2_T_*x*_ and ion‐driven Ti_3_C_2_T_*x*_ nanosheets. A) Experimental scheme of pristine Ti_3_C_2_T_*x*_ synthesis following in situ ion intercalation of Ti_3_C_2_T_*x*_ nanosheets. B) SEM of pristine Ti_3_C_2_T_*x*_, demonstrating a clear layer‐by‐layer structure. C) SEM of ion‐driven Ti_3_C_2_T_*x*_ nanosheets, highlighting the expansion of the layer‐by‐layer structure. D) SEM–EDS mapping of ion‐driven Ti_3_C_2_T_*x*_ nanosheets at 1um scale. E) SEM–EDS mapping of titanium (Ti) of ion‐driven Ti_3_C_2_T_*x*_ nanosheets. F) SEM–EDS mapping of fluorine (F) of ion‐driven Ti_3_C_2_T_*x*_ nanosheets. G) SEM–EDS mapping of calcium (Ca) of ion‐driven Ti_3_C_2_T_*x*_ nanosheets. H) XRD of MAX phase, pristine Ti_3_C_2_T_*x*_, and ion‐driven Ti_3_C_2_T_*x*_ nanosheets. I) High‐resolution XRD of the 002 peak of MAX phase, pristine Ti_3_C_2_T_*x*_, and ion‐driven Ti_3_C_2_T_*x*_ nanosheets. J) XPS deconvolution of the C 1*s* peak in ion‐driven Ti_3_C_2_T_*x*_ nanosheets. K) XPS deconvolution of the O 1*s* peak in ion‐driven Ti_3_C_2_T_*x*_ nanosheets. L) XPS deconvolution of the Ti 2*p* peak in ion‐driven Ti_3_C_2_T_*x*_ nanosheets. M) XPS deconvolution of the Ca 2*p* peak of ion‐driven Ti_3_C_2_T_*x*_ nanosheets and pristine Ti_3_C_2_T_*x*_ nanosheets.

MXene (Ti_3_C_2_T_*x*_) was synthesized by etching aluminum from Ti_3_AlC_2_ using a solution of LiF in HCl, followed by stirring, washing, and centrifugation to obtain Ti_3_C_2_T_*x*_ nanosheets, as reported in our previous work.^[^ ^23^ ^]^ The synthesized Ti_3_C_2_T_*x*_ was stirred with a 0.5 m CaCl_2_·2H_2_O solution, followed by bath sonication, as illustrated in **Figure** **1**A.

The surface morphology and elemental composition of Ti_3_C_2_T_*x*_ nanosheets and ion‐driven Ti_3_C_2_T_*x*_ nanosheets, as well as the intercalation structure, were analyzed using SEM–EDS, TEM–EDS, and XRD analysis. Figure 1B shows the high‐resolution SEM of Ti_3_C_2_T_*x*_ nanosheets at a 100 nm scale, demonstrating the delaminated and uniform layer‐by‐layer structure. Upon addition of ions, the layer‐by‐layer structure of Ti_3_C_2_T_*x*_ nanosheets expanded significantly due to intercalation of Ca^2+^ ions and electrostatic screening of Cl^−^ ions, which can be visualized in Figure 1C.

Figure 1D–G and S1A–D, Supporting Information, illustrate the SEM–EDS mapping photograph (Figure 1D) at a 1 μm scale. Figure S1A, Supporting Information, shows the homogenous distribution of the combined elemental map of ion‐driven Ti_3_C_2_T_*x*_ nanosheets, which was further quantified with all key elements, including carbon (Figure S1B, Supporting Information), oxygen (Figure S1C, Supporting Information), titanium (Figure 1E), fluorine (Figure 1F), calcium (Figure 1G), and chlorine (Figure S1D, Supporting Information). Moreover, the atomic percentages of all elements were measured to confirm the successful synthesis of Ti_3_C_2_T_*x*_ nanosheets and ion‐driven Ti_3_C_2_T_*x*_ nanosheets, as highlighted in Table S1, Supporting Information. In line with SEM–EDS, TEM–EDS mapping was performed to examine the ion‐driven Ti_3_C_2_T_*x*_ (Figure S2A–G, Supporting Information) at a scale of 200 nm, investigating the surface structure and intercalated ions that maintain their structural integrity and functionality after intercalation.

The high‐resolution surface morphology of ion‐driven Ti_3_C_2_T_*x*_ is shown in Figure S2A, Supporting Information, followed by homogeneous elemental mapping including carbon (Figure S2B, Supporting Information), oxygen (Figure S2C, Supporting Information), titanium (Figure S2D, Supporting Information), fluorine (Figure S2E, Supporting Information), calcium (Figure S2F, Supporting Information), and chlorine (Figure S2G, Supporting Information).

The SEM–EDS and TEM–EDS result shows effective in situ intercalation of Ca^2+^ and Cl^−^ ions, which increases interlayer spacing, possibly increasing ion mobility and the stability of Ti_3_C_2_T_*x*_ nanosheets.^[^ ^24^ ^]^ In situ Ca^2+^ intercalation, Cl^−^ electrostatic repulsion, and other functional groups of O's and OH activate more sites, which enhance extreme stability and accelerate the hydrogel gelation process.^[^ ^25^ ^]^ In summary, the homogeneous elemental distribution and structural features of Ti_3_C_2_T_*x*_ nanosheets tune their conductivity and stability.

The interlayer expansion of Ti_3_AlC_2_, Ti_3_C_2_T_*x*_ nanosheets, and ion‐driven Ti_3_C_2_T_*x*_ nanosheets was examined by XRD, as illustrated in Figure 1H–I. The corresponding interlayer distance peak (002), shifted from 9.69° to 7.46°, demonstrates the successful synthesis of Ti_3_C_2_T_*x*_ nanosheets from Ti_3_AlC_2_. The interlayer spacing peak (002) further decreases to 6.86° from 7.46° upon intercalation of Ca^2+^ ions and electrostatic repulsion of Cl^−^ ions. Moreover, to ensure the successful ion‐driven engineering of Ti_3_C_2_T_*x*_ nanosheets, Bragg's law (*nλ* = 2*d*sin*θ*)^[^ ^26^ ^]^ was employed to calculate the *d*‐spacing from the XRD (002) peak data. The calculations (Table S2, Supporting Information) demonstrated the *d*‐spacing corresponding to the expansion of the interlayer spacing of Ti_3_AlC_2_, Ti_3_C_2_T_*x*_ nanosheets, and ion‐driven Ti_3_C_2_T_*x*_ nanosheets from 9.11 to 11.83 and 12.86 Å, respectively. Additionally, the ion‐driven interfacial engineering of Ca^2+^ and Cl^−^ ions enhances the dispersibility of Ti_3_C_2_T_*x*_ within the solution, as demonstrated by UV–vis absorbance investigations (Figure S3, Supporting Information). The dispersion of Ti_3_C_2_T_*x*_ nanosheets increases with the concentration of CaCl_2_. At the highest concentration of 1 m, the dispersion was very high; however, this can lead to a high crosslinking density. To balance the higher stretchability and better dispersion, a 0.5 m CaCl_2_ solution was quantified for further investigation, making it a promising condition for high‐performance wearable ECG electrodes.

XPS analysis was performed to investigate the electronic structure, chemical structure, and state of bonding in pristine Ti_3_C_2_T_*x*_ nanosheets and ion‐driven Ti_3_C_2_T_*x*_ nanosheets, to elucidate the roles of in situ Ca^2+^ intercalation and Cl^−^ electrostatic repulsion on enhancing active sites and stability of Ti_3_C_2_T_*x*_ and thereby advancing the hydrogel gelation process with excellent stretchability for physiological monitoring.

Figure S4A, Supporting Information, shows the XPS survey of pristine Ti_3_C_2_T_*x*_ nanosheets (black graph) and ion‐driven Ti_3_C_2_T_*x*_ nanosheets (red graph), indicating the core‐level elements: C 1*s*, F 1*s*, O 1*s*, and Ti 2*p*, as well as an additional peak of Ca^+2^ following intercalation, which confirms the successful intercalation of ions in pristine Ti_3_C_2_T_*x*_ nanosheets. The C 1*s* spectra of pristine Ti_3_C_2_T_*x*_ nanosheets (Figure S4B, Supporting Information) demonstrated peaks at C—Ti—T_*x*_ (282 eV),^[^ ^27^ ^]^ C—C (284.6 eV),^[^ ^28^ ^]^ and C—O, CH_*x*_ (286.8 eV).^[^ ^29^ ^]^ In ion‐driven Ti_3_C_2_T_*x*_ nanosheets Figure 1J, the peak C—Ti—T_*x*_ (≈282.1 eV) decreases due to surface termination by oxygen‐rich binding sites (C—Ti—O), which destabilizes charge distribution and reduces the Ti—C bond.^[^ ^30^ ^]^ Concurrently, the C—C (285 eV) increment indicates the enhancement of the electrical conductivity by providing percolation channels and maintaining interlayer space for Ca^2+^ and Cl^−^ mobility. The Ca^2+^ stabilizes oxygen groups through ionic interactions as supported by the binding energy C—O peak (≈286.5 eV), which is similar to the hydration shell effect that was realized in Mg^2+^‐intercalated Ti_3_C_2_T_*x*_.^[^ ^26^ ^]^

O 1*s* spectra of pristine Ti_3_C_2_T_*x*_ nanosheets are illustrated in Figure S4C, Supporting Information, showing peaks at 530 eV (TiO_2_), 531.2 eV (C—Ti—O_*x*_), 532 eV (C—Ti—(OH_*x*_)), 532.9 eV (Al_2_O_3_—(OR)), and 533.8 eV (H_2_Oads (IV)—OR).^[^ ^31^, ^32^ ^]^ On the intercalation of Ca^2+^ and electrostatic repulsion due to Cl^−^, the corresponding peaks (Figure 1K) shifted uniformly to lower energies: 529.7 eV (TiO_2_), 530.9 eV (C—Ti—O_*x*_), 531.8 eV (C—Ti—(OH_*x*_)), 532.8 eV (Al_2_O_3_—(OR)), and 533.5 eV (H_2_Oads (IV)—OR), indicating a shift due to significant changes in electron density around oxygen atoms.^[^ ^33^ ^]^ Additionally, the 533.5 eV (H_2_Oads (IV)—OR) hydration layer peak is higher in intensity, indicating a strong hydration shell formed by the intercalation of Ca^2+^, which enhances stability and introduces additional functional groups.^[^ ^34^, ^35^ ^]^

The pristine Ti_3_C_2_T_*x*_ nanosheets Ti 2*p* spectra are shown in Figure S4D, Supporting Information, demonstrating a multititanium environment with the coexistence of peaks for Ti (I, II, or IV) (455 and 461.2 eV), Ti^2+^ (I, II, or IV) (455.8 and 461.5 eV), Ti^3+^ (I, II, or IV) (457.3 and 463.1 eV), TiO_2_ (458.5 and 464.5 eV), and TiO_2_–*x*F_*x*_ (459.2 eV). However, upon intercalation of Ca^2+^ intercalation and electrostatic repulsion of Cl^−^ ions (Figure 1L), the titanium complex environment shows reduction trend in peaks of Ti (I, II, or IV) peaks (454.7 and 460.21 eV), Ti^2+^ (I, II, or IV) peaks (455.5 and 461.2 eV), and Ti^3+^ (I, II, or IV) peaks (457 and 462.4 eV), highlighting the increased electron density surrounding the titanium atoms.^[^ ^31^ ^]^ The persistent peak separation for Ti^0^/Ti^2+^/Ti^3+^ reflects structural integrity after ion‐driven interfacial engineering. The low change compared to TiO_2_ (458.4 and 464.2 eV) suggests that the intercalation has a reduced effect on oxide groups. TiO_2_–*x*F_*x*_ oxidation (459.5 eV) reflects increased electron pull‐up by fluorine, likely from structural readjustments after Ca^+2^ and Cl^−^ incorporation. The increased density of the Ca^2+^ charge enhances the surface character of Ti_3_C_2_T_*x*_, resulting in outstanding stability.^[^ ^25^ ^]^

The F1 spectra of pristine Ti_3_C_2_T_*x*_ nanosheets (Figure S4E, Supporting Information) contain the peaks corresponding to fluorine termination, such as C—Ti—F_*x*_ (684.9 eV) and TiO_2_–*x*F_*x*_ (685.3 eV), owing to HF etching.^[^ ^36^ ^]^ In addition, Al—F_*x*_ (686.4 eV) and Al—OF_*x*_ (687.5 eV) peaks indicate the presence of aluminum residues.^[^ ^31^ ^]^ In ion‐driven Ti_3_C_2_T_*x*_ nanosheets, as shown in (Figure S4F, Supporting Information), the C—Ti—F_*x*_ peak (685.1 eV) and the TiO_2–*x*_F_*x*_ peak (686.2 eV) both shift to higher binding energies, which signifies the introduction of additional surface functionalities through oxidation by interaction with Ca^2+^ and Cl^−^ ions.

Lastly, the Ca 2*p* spectra were scanned on pristine Ti_3_C_2_T_*x*_ nanosheets and ion‐driven Ti_3_C_2_T_*x*_ nanosheets to confirm the successful ion interfacial engineering of ions, as shown in Figure 1M. The pristine Ti_3_C_2_T_*x*_ in black graph shows no peaks on the scan, while in ion‐driven Ti_3_C_2_T_*x*_, the calcium peaks appear at 348.5 eV (Ca 2*p*_3_/_2_) and 352.0 eV (Ca 2*p*_1_/_2_).^[^ ^26^ ^]^ This is indicative of electrostatic interaction with oxygen‐containing functional groups.^[^ ^37^ ^]^ Overall, the elemental and chemical states of ions enhance the hydrophilicity, surface activity, and stability of Ti_3_C_2_T_*x*_, making it suitable for ultrastretchable, fast‐gelated hydrogels for long‐term physiological monitoring. Furthermore, the detailed XPS peaks are presented in Table S3, Supporting Information.

### 2.2 Mechanistic Analysis of Ion Interactions in Ti_3_C_2_T_*x*_–PAM Hydrogel

This section demonstrated the synthesis mechanism of the ion‐driven Ti_3_C_2_T_*x*_−activated PAM hydrogel, supported with noncovalent interaction simulations to understand the mechanism and interactions at the molecular level.

**Figure 2:**
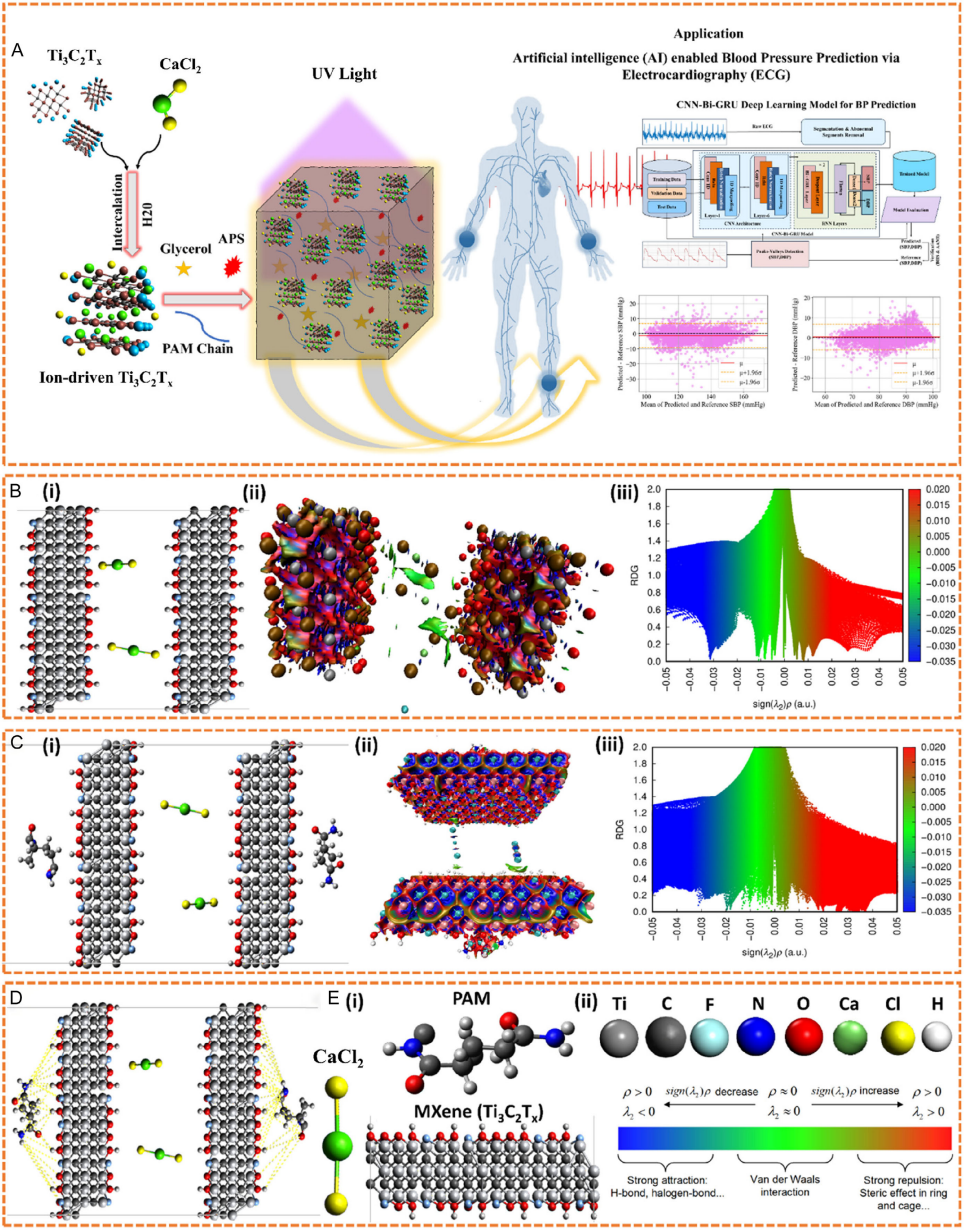
Analysis of a rapidly gelled ultrastretchable ion‐driven Ti_3_C_2_T_*x*_−PAM hydrogel via NCIs. A) Graphical illustration of in situ Ca^+2^ and Cl^−^ Intercalation of Ti_3_C_2_T_*x*_ nanosheets reinforces PAM hydrogel, started with the experimental procedure followed by the BP prediction from ECG data via CNN–BiGRU model. B) NCIs of Ti_3_C_2_T_*x*_ and CaCl_2_: (i) molecular structure of Ti_3_C_2_T_*x*_ and CaCl_2_, (ii) NCI visuals of Ti_3_C_2_T_*x*_ and CaCl_2_, (iii) RDG of Ti_3_C_2_T_*x*_ and CaCl_2_. C) NCIs of Ti_3_C_2_T_*x*_, PAM, and CaCl_2_: (i) molecular structure of Ti_3_C_2_T_*x*_, PAM, and CaCl_2_, (ii) NCI visuals of Ti_3_C_2_T_*x*_, PAM, and CaCl_2_, (iii) RDG of Ti_3_C_2_T_*x*_, PAM, and CaCl_2_. D) Hydrogen bonding between Ti_3_C_2_T_*x*_, PAM, and CaCl_2_. E) Molecular structure of Ti_3_C_2_T_*x*_, PAM, and CaCl_2_ (i), and color coding of the element and RDG graph (ii).

The synthesis mechanism, as highlighted in **Figure** **2**A, demonstrates the in situ intercalation of pristine Ti_3_C_2_T_*x*_ nanosheets with CaCl_2_ in aqueous solution to form the ion‐driven Ti_3_C_2_T_*x*_ solution. A specific quantity of AM, MBAA, APS, and Glycerol (Table S4, Supporting Information) was added into the ion‐driven Ti_3_C_2_T_*x*_ solution, followed by focused UV light to crosslink the ion‐driven Ti_3_C_2_T_*x*_−activated PAM hydrogel. The ion‐driven interfacial engineering of Ca^2+^ and Cl^−^ ions reinforced the stability of interlayer spacing in Ti_3_C_2_T_*x*_ nanosheets, boosting the crosslinking and gelation time within the polymeric matrix.^[^ ^38^ ^]^ This strategy developed an ultrastretchable hydrogel with high electrical conductivity.

Noncovalent interactions (NCIs) are crucial to determining the molecular behavior of hydrogel systems. Figure 2B–E and S5A–B, Supporting Information, show molecular interactions calculated by Avogadro, Gaussian, and Multiwfm software.^[^ ^39^ ^]^ These investigations feature reduced density gradient (RDG) data plotted against sign(*λ*_2_)*ρ*. Blue regions indicate strong, attractive interactions such as hydrogen and halogen bonds. Green areas represent weak van der Waals forces, and red indicates repulsive interactions or steric effects of substantial magnitude, such as those caused by rings and cages. Generally, an electron density (*ρ*) less than 0.1 a.u. indicates noncovalent interactions, while greater than 0.2 a.u. shows covalent bonding. These thresholds interpret the various types of interactions,^[^ ^40^, ^41^ ^]^ as shown in Figure 2E(ii).

Figure S5A(i), Supporting Information, illustrates the PAM interactions with CaCl_2_. The chemical structure (Figure S5A(ii), Supporting Information) describes the interactions of Ca^2+^ ions with PAM's amide groups.^[^ ^42^ ^]^ The corresponding RDG plot (Figure S5A(iii), Supporting Information) shows broad regions of blue areas signifying high attractions between Ca^2+^ ions and O^2−^ atoms in PAM's amide groups, some green regions highlight the moderate van der Waals interaction, and several red spots signify steric repulsion.^[^ ^40^ ^]^ These interactions contributed to crosslinking in the hydrogel network, as Ca^2+^ is reported to be an ionic crosslinker for PAM polymer chains.^[^ ^43^ ^]^

Figure 2B(i) demonstrates the interactions between Ti_3_C_2_T_*x*_ nanosheets and CaCl_2_. The chemical structure (Figure 2B(ii)) shows the behavior of Ca^2+^ and Cl^−^ ions with Ti_3_C_2_T_*x*_ nanosheets. However, the RDG analysis (Figure 2B(iii)) reveals significant blue regions for strong electrostatic attraction by intercalation of Ca^2+^ ions, irregular green regions for van der Waals interaction, and strong red regions for repulsive interactions contributed by Cl^−^ ions in the close packing of the structure.^[^ ^40^ ^]^ This NCI study confirms the effective ion‐driven interfacial engineering of Ca^+2^ and Cl^−^ ions with Ti_3_C_2_T_*x*_ nanosheets, retaining the electrical and structural properties necessary for long‐term ECG monitoring employing developed hydrogel electrodes.

Figure S5B, Supporting Information, shows the interactions between Ti_3_C_2_T_*x*_ nanosheets and PAM without Ca^2+^ and Cl^−^ (Figure S5B(i), Supporting Information). Direct interactions between PAM molecules and Ti_3_C_2_T_*x*_ nanosheets are depicted (Figure S5B(ii), Supporting Information). The RDG plot (Figure S5B(iii), Supporting Information) illustrates moderate blue regions indicating hydrogen bonding, extensive green areas representing weak van der Waals interactions at the interface, and minimal red regions reflecting limited steric hindrance.^[^ ^40^ ^]^ These interactions align with previous studies, which show that polymer chains can form noncovalent bonds with Ti_3_C_2_T_*x*_ surface terminations, primarily through hydrogen bonding (Figure 2D).^[^ ^44^ ^]^

The NCIs of ion‐driven Ti_3_C_2_T_*x*_–PAM hydrogel are depicted in Figure 2C. The structural model features (Figure 2C(i) a composite hydrogel with Ti_3_C_2_T_*x*_ nanosheets layered between PAM chains, where Ca^2+^ ions serve as intercalant with polymeric ionic crosslinking and Cl^−^ as electrostatic repulsive ions. The molecular structure (Figure 2C(ii)) and RDG map (Figure 2C(iii)) display a dominance of blue dots, indicating strong, attractive interactions, with green patches highlighting the importance of van der Waals forces for structural stability and flexibility.^[^ ^40^ ^]^ The blue areas in the RDG are characteristic of strong ionic and hydrogen bonding interactions (Figure 2D). In contrast, the green and zero red spots indicate the ultrastretchability and effective packing ability of the hydrogel, respectively, without significant voids.^[^ ^45^ ^]^ In addition, Figure 2E(i) is the molecular structure of the PAM, CaCl_2_, and Ti_3_C_2_T_*x*_ layer, and Figure 2E(ii) is the color coding of the elements and the RDG graph.

This NCI's investigation provides molecular‐level interpretations for the macroscopic behavior of ion‐driven Ti_3_C_2_T_*x*_–PAM hydrogels. Repulsive and attractive interactions give rise to a stable energy profile that induces structural cohesion and facilitates stress‐induced dynamic reconfiguration. The ionic crosslinking and noncovalent surface contacts between ion‐driven Ti_3_C_2_T_*x*_ and PAM polymers design strategy enable the development of next‐generation soft materials with tailor‐made mechanical and conductive properties for long‐term ECG monitoring.

### 2.3 Physical and Structural Analysis of Ion‐Driven Ti_3_C_2_T_*x*_–PAM Hydrogel

This section outlines the real‐time behavior of ion‐driven Ti_3_C_2_T_*x*_ within the PAM polymeric complex matrix using MD, supported by morphological, chemical, thermal, and structural investigations using SEM–EDS, Fourier transform infrared spectroscopy (FTIR), X‐ray photoelectron spectroscopy (XPS), thermogravimetric analysis (TGA), and differential scanning calorimetry (DSC).

**Figure 3:**
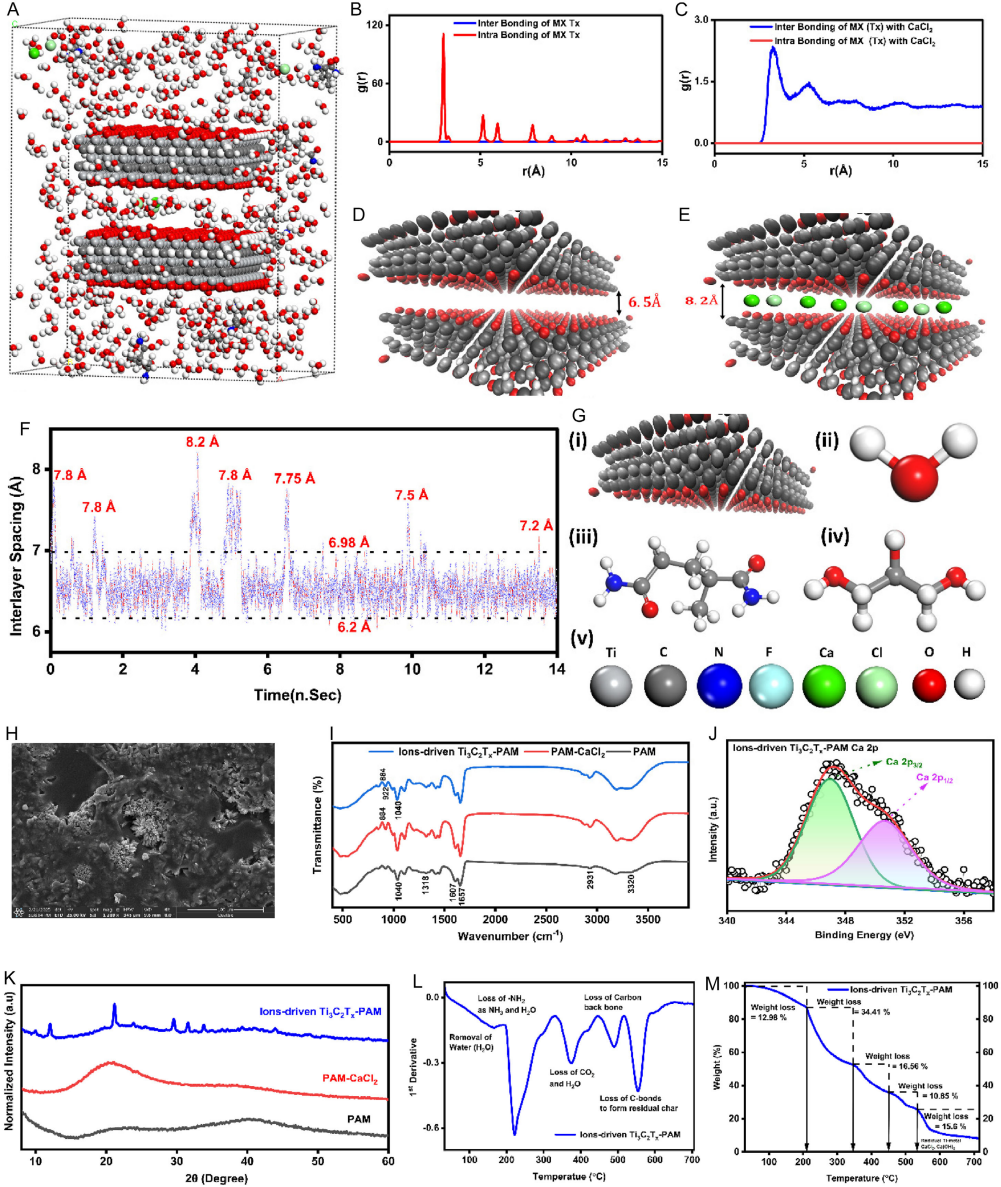
MD, morphological, elemental, structural, and chemical characterization of PAM, PAM–CaCl_2_, and ion‐driven Ti_3_C_2_T_*x*_–PAM hydrogels. A) MD simulation model of ion‐driven Ti_3_C_2_T_*x*_–PAM hydrogel, B) radial density function (RDF) plot of the inter bonding of MX T_*x*_ (Ti_3_C_2_T_*x*_) and intrabonding of MX T_*x*_, C) RDF plot of the inter bonding of MX T_*x*_ with CaCl_2_ and intrabonding of MX T_*x*_ with CaCl_2_, D) MXene layers with initial 6.5 Å interlayer spacing, and E) MXene layers with 8.2 Å interlayer spacing while in reaction. F) Interlayer spacing of Ti_3_C_2_T_*x*_ in the complex environment is flexible (6.2–8.2 Å) over time (14 n.s). G) Structure of Ti_3_C_2_T_*x*_ (i), H_2_O (ii), PAM (iii), and glycerol (iv), along with color coding of atoms used in MD simulations (v). H) SEM image of ion‐driven Ti_3_C_2_T_*x*_−PAM hydrogel. I) FTIR of PAM, PAM–CaCl_2_, and ion‐driven Ti_3_C_2_T_*x*_–PAM hydrogels, J) XPS deconvolution of the Ca 2*p* peak of ion‐driven Ti_3_C_2_T_*x*_–PAM hydrogel. K) XRD of PAM, PAM–CaCl_2_, and ion‐driven Ti_3_C_2_T_*x*_–PAM hydrogel. L) DSC graph of ion‐driven Ti_3_C_2_T_*x*_ hydrogel. M) TGA graph of ion‐driven Ti_3_C_2_T_*x*_–PAM hydrogel.

**Figure** **3**A illustrates the complex MD simulation model, which consists of a Ti_3_C_2_T_*x*_ nanosheet placed between the PAM, glycerol, Ca^2+^, and Cl^−^ ions with aqueous solvent. This strategic arrangement forms a stable 3D hydrogel model to investigate the behavior of Ti_3_C_2_T_*x*_ in the presence of ions and its surrounding environment. Figure 3B,C shows the radial distribution function (RDF) plots of pristine Ti_3_C_2_T_*x*_ nanosheet and ion‐intercalated Ti_3_C_2_T_*x*_ nanosheets in a complex 3D environment. As demonstrated in Figure 3B, the RDF of pristine Ti_3_C_2_T_*x*_ exhibits sharp and high peaks at short distances for intralayer bonding (red curve), indicating strong and well‐defined interactions in intralayers. For comparison, interlayer bonding (blue curve) is low and flat,^[^ ^46^ ^]^ revealing weak interactions between layers due to the absence of Ca^2+^ and Cl^−^ ions. However, in Figure 3C, the RDF of Ti_3_C_2_T_*x*_ with Ca^2+^ and Cl ions reveals, in the interlayer bonding (blue curve), a strong peak at ≈3–5 Å distance due to stable interlayer interactions through the intercalation of Ca^2+^ ions and the repulsion by Cl^−^ ions. Intralayer bonding (red curve) remains largely unchanged, indicating that Ca^2+^ and Cl^−^ ions have a minimal effect on bonding within individual layers. The emergence of a strong interlayer peak and shift of the blue curve to higher *r* values are evidence that Ca^2+^ ions act as molecular pillars, stabilizing the interlayer structure via coordination with surface oxygen groups. Meanwhile, Cl^−^ ions reduce electrostatic repulsion among negatively charged layers, allowing them to move further apart and increase the interlayer spacing.^[^ ^47^, ^48^ ^]^

Figure 3D illustrates the inherent bonding character of the Ti_3_C_2_T_*x*_ nanosheet model, demonstrating well‐ordered atomic structures with a 6.5 Å interlayer spacing. Interestingly, upon the addition of Ca^2+^ and Cl^−^ ions, Ti_3_C_2_T_*x*_ nanosheets demonstrated flexible behavior in interlayer spacing, which expands from 6.2 to 8.2 Å (Figure 3E,F). This behavior interprets the Ca^2+^ intercalation and Cl^−^ electrostatic repulsion within a complex 3D polymeric matrix, enhancing mechanical, electrical, and electrochemical performance of the developed hydrogel.

In conclusion, the MD analysis supports the demonstrated mechanism of temporal behavior of ion‐driven Ti_3_C_2_T_*x*_ in 3D polymeric structures, retaining the electrical conductivity of hydrogel with ultrastretchable properties. Moreover, Figure 3G illustrates the structure of Ti_3_C_2_T_*x*_ (i), H_2_O (ii), PAM (iii), Glycerol (iv), and color coding (v) of atoms used in MD simulations.

Morphological analysis, along with elemental distribution, of the ion‐driven Ti_3_C_2_T_*x*_–PAM hydrogel was investigated using SEM–EDS techniques, as shown in Figure 3H and S6A–F, Supporting Information. The SEM micrograph of the ion‐driven Ti_3_C_2_T_*x*_–PAM hydrogel (Figure 3H) shows highly porous, interconnected surfaces with some flat areas, likely due to the presence of glycerol, which acts as a plasticizer within the hydrogel network.^[^ ^49^ ^]^ The EDS elemental mapping, as shown in Figure S6A–F, Supporting Information, confirms the presence and distribution of key elements in the hydrogel. Fluorine (F) in Figure S6A, Supporting Information, and titanium (Ti) in Figure S6B, Supporting Information, present a homogeneous distribution, revealing the well‐aligned Ti_3_C_2_T_*x*_ nanosheets within the hydrogel matrix,^[^ ^50^ ^]^ which enhances electrical conductivity and mechanical strength. Figure S6C, Supporting Information, presents the high concentration of carbon (C) in the ion‐driven Ti_3_C_2_T_*x*_–PAM hydrogel, as it is the fundamental element of Ti_3_C_2_T_*x*_ and the PAM polymer.

Oxygen (O) distribution, as shown in Figure S6D, Supporting Information, is consistent with the presence of hydrophilic functional groups (—OH, —O, —COOH) on Ti_3_C_2_T_*x*_ and PAM polymers, which promotes surface functionalization.^[^ ^51^ ^]^ There was a low concentration of nitrogen (Figure S6E, Supporting Information) due to the APS polymerizing agent, with a discrete concentration of calcium (Figure S6F, Supporting Information), supporting the intercalation of Ti_3_C_2_T_*x*_ nanosheets. In Conclusion, SEM–EDS analysis of ion‐driven Ti_3_C_2_T_*x*_–PAM hydrogel demonstrated a uniform microstructure with interconnected porosity and homogeneous distribution of (F, Ti, C, O, N and Ca), thereby confirming the hydrogel's efficiency as an advanced, highly stretchable, and conductive material for long‐term, high‐performance ECG monitoring.

FTIR analyzed the vibrational and molecular structures of the PAM, PAM–CaCl_2_, and ion‐driven Ti_3_C_2_T_*x*_–PAM hydrogels as illustrated in Figure 3I. The FTIR of base sample PAM hydrogel (gray plot in Figure 3I) demonstrated the characteristic peaks corresponding to N—H stretching at 3320 cm^−^^1^, C—H stretching at 2931 cm^−^^1^, C=O stretching at 1657 cm^−^^1^, N—H bending at 1607 cm^−^^1^, C—N stretching at 1318 cm^−^^1^, and C—C stretching at 1040 cm^−1^.^[^ ^52^ ^]^ With the addition of CaCl_2_, the spectrum (red graph in Figure 3I) exhibits all PAM's characteristic peaks with increased intensity in addition to another peak at 884 cm^−1^, which is likely due to Ca—O or Ca—Cl stretching.^[^ ^53^ ^]^ The appearance of main peaks at 1040 and 922 cm^−1^ might be due to Ti—O or Ca—Ti interactions (blue graph in Figure 3I), demonstrating the presence of Ti_3_C_2_T_*x*_ nanolayers.^[^ ^51^ ^]^ These spectral changes suggest that the ion‐driven intercalation of Ti_3_C_2_T_*x*_ and the incorporation of Ca^2+^ and Cl^−^ strengthen hydrogen bonding and ionic interactions, thereby improving the mechanical strength and stability of the hydrogel while maintaining the PAM core structure.^[^ ^54^ ^]^

XPS was used to investigate the electronic structure, chemical composition, and bonding states of PAM, PAM–CaCl_2_, and ion‐driven Ti_3_C_2_T_*x*_–PAM hydrogels, revealing the influence of in situ intercalation of Ca^2+^ and Cl^−^ on the gelation process, which led to improved mechanical properties of the PAM hydrogel. Figure S7, Supporting Information, displays the XPS scans, which reveal the chemical and electrical states of PAM, PAM–CaCl_2_, and ion‐driven Ti_3_C_2_T_*x*_–PAM hydrogel. The PAM spectrum (Figure S7, Supporting Information, gray graph) shows peaks for C 1*s*, N 1*s*, and O 1*s*, corresponding to the PAM amide backbone and functional groups. The PAM–CaCl_2_ spectrum (Figure S7, Supporting Information, red graph) shows a Ca 2*p* peak, confirming Ca^2+^ presence and ionic interactions with PAM functional groups. In contrast, the Ti_3_C_2_T_*x*_–PAM hydrogel spectrum (Figure S7, Supporting Information, blue graph) displays additional Ti 2*p*, C, N, O, and Ca peaks, indicating a hybrid structure with complex interactions between Ti_3_C_2_T_*x*_ and Ca^2+^ that enhance crosslinking and mechanical stability.

The C 1*s* XPS analysis reveals that PAM exhibits peaks (Figure S8A, Supporting Information) at 285 eV (C—C), 286.2 eV (C—O/C—N), and 288.1 eV (C=O), which correspond to the polymer backbone and amide groups.^[^ ^55^ ^]^ For PAM–CaCl_2_, Figure S9A, Supporting Information, indicates slight shifts to 285.1 eV (C—C), 286.2 eV (C—O/C—N), and 288.6 eV (C=O), indicating stronger interactions between the Ca^2+^ and the nitrogen or oxygen atoms in PAM, possibly through ionic bonds.^[^ ^55^, ^56^ ^]^ However, Figure S10A, Supporting Information, shows further minor shifts at 285.3 eV (C—C), 286.6 eV (C—O/C—N), and 288.5 eV (C=O),^[^ ^57^ ^]^ which reflect enhanced electrical interactions between PAM, Ti_3_C_2_T_*x*_, and Ca^2+^ and Cl^−^. The interaction of Ti_3_C_2_T_*x*_ facilitates electron extraction through hydrogen bonding or van der Waals interactions.^[^ ^58^ ^]^ The slight decrease in the (C=O) peak against PAM–CaCl_2_ is indicative of the stabilization of the carbonyl oxygen by Ti_3_C_2_T_*x*_, which moderates the electron‐withdrawing effect of Ca^2+^. Additionally, a new peak at 284.4 eV (Ti–C) in ion‐engineered Ti_3_C_2_T_*x*_–PAM hydrogel confirms the effective integration of Ti_3_C_2_T_*x*_ into the polymer matrix.^[^ ^36^ ^]^

O 1*s* spectra of PAM–CaCl_2_ in Figure S9B, Supporting Information, show peaks at ≈531.9 eV (C=O) and 532.8 eV (O—H),^[^ ^59^ ^]^ corresponding to the amide and hydroxyl groups of PAM chains, respectively. The slight shift from pure PAM O1*s* spectra (Figure S8B, Supporting Information) indicates interactions between Ca^2+^ and OH groups within PAM, enhancing ionic crosslinking.^[^ ^60^ ^]^ However, the ion‐driven Ti_3_C_2_T_*x*_–PAM hydrogel spectrum (Figure S10B, Supporting Information) demonstrates weak shifts at 532 eV (C=O) and 533 eV (O—H), indicating strong Ca^2+^ intercalation and strong Cl^−^ repulsive electrostatic interaction with Ti_3_C_2_T_*x*_ with increased crosslinking density and mechanical stability.

N 1*s* spectra of PAM, PAM–CaCl_2_, and ion‐driven Ti_3_C_2_T_*x*_–PAM hydrogel provide a detailed analysis of nitrogen bonding states and interfacial interaction. Figure S8C, Supporting Information, illustrates the N 1*s* spectrum of pure PAM with peaks at 399.5 eV (C—N) and 400.2 eV (N—H_2_)^[^ ^61^ ^]^ characteristic of amide groups. However, the addition of CaCl_2_ results in a shift of the peak to 400.1 eV (C—N) (Figure S9C, Supporting Information), accompanied by an increase in intensity, indicating the formation of a packed Ca^2+^ and Cl^−^ coordination with the carbonyl oxygen and high crosslinking. In the ion‐driven Ti_3_C_2_T_*x*_–PAM hydrogel (Figure S1C, Supporting Information), the C—N peak is shifted to 400.5 eV with strong intensity, indicating successful intercalation of Ca^2+^ and Cl^−^ ions in Ti_3_C_2_T_*x*_, which results in ultrastretchability and rapid gelation. Meanwhile, the N—H_2_ peak remains constant at 400.2 eV.

The Ca 2*p* spectra (Figure S9D, Supporting Information) of PAM–CaCl_2_ exhibit peaks at ≈347.8 eV (Ca 2*p*_3/2_) and 351.3 eV (Ca 2*p*_1/2_),^[^ ^62^ ^]^ indicating ionic coordination with the oxygen and nitrogen groups of the PAM chain to form a crosslinked stable network. In ion‐driven Ti_3_C_2_T_*x*_–PAM hydrogel, slight shifts of these peaks in Figure 3J reflect additional interactions with Ca^2+^and Ti_3_C_2_T_*x*_,^[^ ^26^ ^]^ which enhances the crosslink density and mechanical strength of the hydrogel. The presence of Ti_3_C_2_T_*x*_ is also supported by a low‐intensity Ti 2*p* peak in the ion‐driven Ti_3_C_2_T_*x*_–PAM hydrogel (Figure S10D, Supporting Information), observed at 454–460 eV.^[^ ^63^ ^]^ Additionally, the complete deconvoluted peaks of all the hydrogel samples are provided in Table S5, Supporting Information These observations collectively support the mechanism of rapid gelation and excellent mechanical stability of the ion‐driven Ti_3_C_2_T_*x*_–PAM hydrogel, suitable for long‐term wearable ECG and physiological monitoring.

XRD analysis also revealed that the control PAM hydrogel (Figure 3K, black graph) has an amorphous structure with extremely few crystalline regions, consistent with its polymeric nature,^[^ ^64^ ^]^ as evidenced by the baseline peaks. Incorporation of CaCl_2_ within the PAM matrix (Figure 3K, red graph) significantly altered the profile, with an increasing intensity of baseline peaks, indicating changes in the polymeric matrix due to ionic interactions between Ca^2+^ and Cl^−^ ions, thereby improving the mechanical properties of the hydrogel.^[^ ^65^ ^]^ Furthermore, the inclusion of ion‐driven Ti_3_C_2_T_*x*_ in the PAM matrix (Figure 3K, blue graph) showed sharp, intense peaks, demonstrating increased crystallinity.^[^ ^66^ ^]^

The DSC and TGA results of PAM, PAM–CaCl_2_, and ion‐driven Ti_3_C_2_T_*x*_–PAM hydrogels are presented in Figure 3L–M and S11A–D, Supporting Information, providing essential insights into their thermal stability and transition temperatures, which are crucial for long‐term physiological monitoring. Specifically, PAM (Figure S11A,C, Supporting Information) exhibits an initial weight reduction of 20.97% up to ≈200 °C due to the evaporation of physically adsorbed water, followed by a 25.14% loss between 200 and 350 °C from the decomposition of amide groups, a 27.68% reduction between 350 and 500 °C resulting from the release of CO_2_ and H_2_O, and a final 18.07% loss between 500 and 700 °C due to the breakdown of carbon bonds, leaving behind residual carbon char.^[^ ^67^ ^]^ PAM–CaCl_2_ (Figure S11B,D, Supporting Information) also exhibits the same initial weight loss on account of water (20.57% up to ≈200 °C). Still, this total weight loss drops to 73.5%, suggesting that CaCl_2_ stabilizes the polymer through ionic interactions. The degradative trend indicates the presence of Ca^2+^‐associated complexes that influence the thermal decomposition process.^[^ ^68^ ^]^ However, the ion‐driven Ti_3_C_2_T_*x*_–PAM hydrogel (Figure 3L,M) experiences a 12.98% loss of water up to ≈200 °C, indicating a dense hydrogel network with fewer unbound water molecules, which is beneficial in maintaining conductivity over long‐term ECG recordings. The subsequent weight loss (34.41% in the range of ≈200–350 °C) accounts for the enhanced degradation with CaCl_2_ and Ti_3_C_2_T_*x*_, followed by a further 16.56% loss in the range 350–500 °C due to the decomposition of CO_2_ and H_2_O, 10.85% loss between 500 and 600 °C due to backbone degradation of carbon, and a final 15.6% loss in the range 600–700 °C, with residues of Ti metal, CaCl_2_, and Ca(OH)_2_.^[^ ^69^ ^]^

The DSC results are characterized by key thermal events, such as the glass transition and melting points, which are of tremendous relevance in assessing the flexibility and mechanics of the hydrogel. The ion‐driven Ti_3_C_2_T_*x*_–PAM hydrogel contains a lower initial water content, which is favorable for long‐term ECG monitoring by reducing signal quality drift due to changes in hydration levels.^[^ ^70^ ^]^ Inorganic residues (Ti metal, Ca(OH)_2_, and CaCl_2_) influence the long‐term biocompatibility and electrochemical behavior of the hydrogels. In general, the incorporation of Ca^2+^, Cl^−^, and Ti_3_C_2_T_*x*_ increases the thermal stability and mechanical performance of PAM hydrogels for practical, long‐term ECG monitoring.

### 2.4 Performance Characteristics of Ion‐Driven Ti_3_C_2_T_*x*_ PAM Hydrogel

This section discusses the mechanical, strain, electrical, electrochemical, and biocompatibility characteristics of ion‐driven Ti_3_C_2_T_*x*_–PAM hydrogel. These properties of hydrogel are the critical requirements for long‐term cardiovascular monitoring.

**Figure 4:**
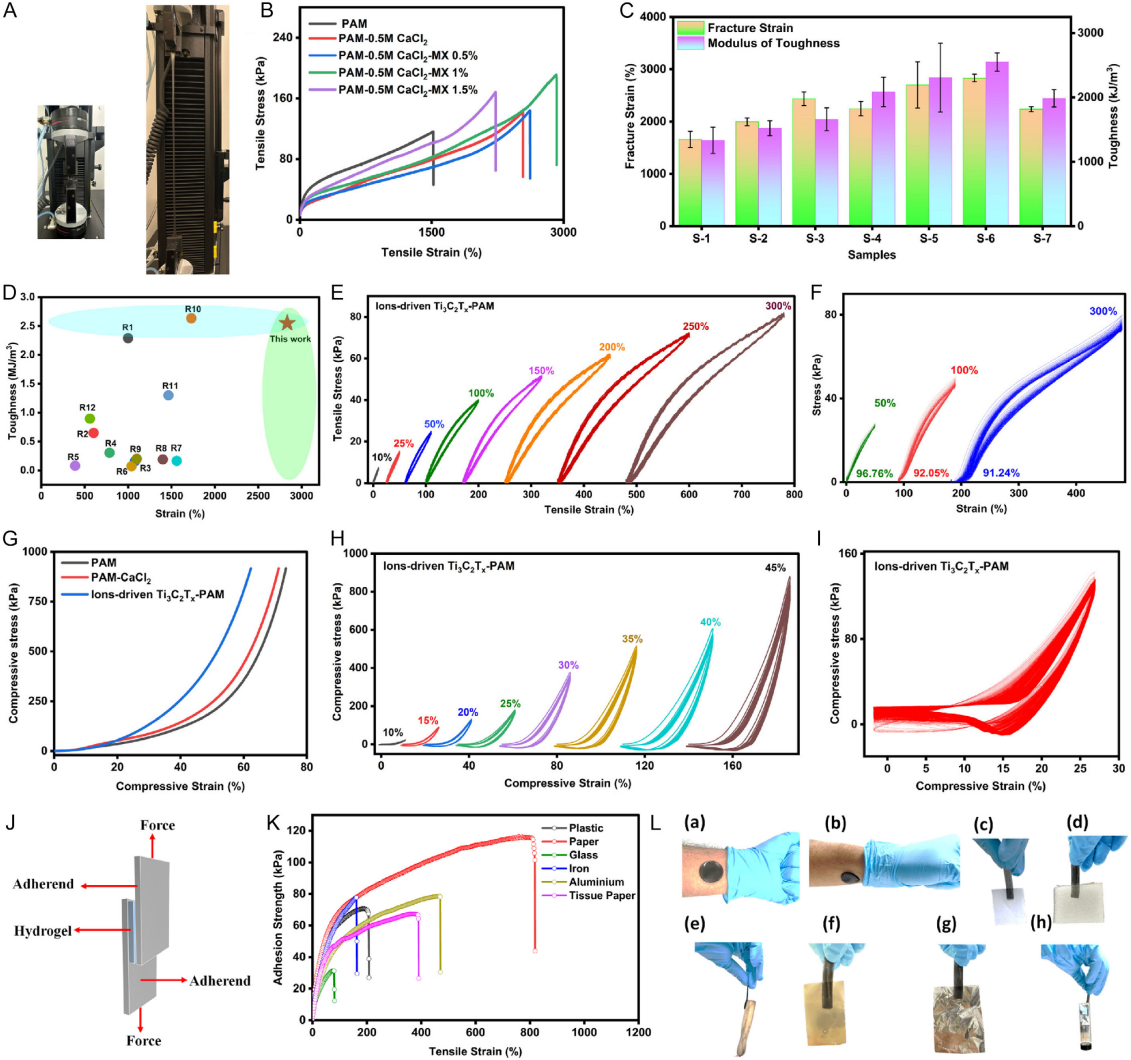
Mechanical performance characterization of PAM, PAM–CaCl_2_, and ion‐driven Ti_3_C_2_T_*x*_–PAM hydrogel. A) Visually mechanical ultrastretchability of ion‐driven Ti_3_C_2_T_*x*_–PAM hydrogel. B) Stress–strain curves for PAM, PAM–0.5 m CaCl_2_, PAM–0.5 m CaCl_2_–MX 0.5%, PAM–0.5 m CaCl_2_–MX 1%, and PAM–0.5 m CaCl_2_–MX 1.5% hydrogels. C) Analysis of fracture strain and toughness for S‐1 (PAM), S‐2 (PAM–0.1 m CaCl_2_), S‐3 (PAM–0.5 m CaCl_2_), S‐4 (PAM–1 m CaCl_2_), S‐5 (PAM–0.5 m CaCl_2_–MX 0.5%), S‐6 (PAM–0.5 m CaCl_2_–MX 1%), and S‐7 (PAM–0.5 m CaCl_2_–MX 1.5%) hydrogels. Error bars represent mean ± SD (*n* = 3). D) Comparative graph of toughness (kJ m^−^^3^) and strain (%) with existing literature values. E) Cyclic stress–strain curves of ion‐driven Ti_3_C_2_T_*x*_–PAM hydrogels at strains of 10%, 25%, 50%, 100%, 150%, and 300%. F) Successive 100‐cycle stress–strain stability of ion‐driven Ti_3_C_2_T_*x*_–PAM hydrogels at 50%, 100%, and 300% strain. G) Compressive stress–strain curves for PAM, PAM–0.5 m CaCl_2_, and ion‐driven Ti_3_C_2_T_*x*_–PAM hydrogels. H) Cyclic compressive stress–strain curves of ion‐driven Ti_3_C_2_T_*x*_–PAM hydrogel at 10–45% incremental strains. I) Consecutive 1000‐cycle cyclic compressive stress–strain analysis of ion‐driven Ti_3_C_2_T_*x*_–PAM hydrogel at 25% strain, demonstrating ultrastability. J) Visual demonstration of a lap‐shear test for analyzing adhesion properties. K) Stress–strain curves showing adhesion strengths of PAM–0.5 _M_ CaCl_2_–MX 1% hydrogel to various substrates, including plastic, paper, glass, iron, aluminum, and tissue paper, highlighting superior adhesion properties. L) Visual demonstration of the adhesion of PAM–0.5_M_ CaCl_2_–MX 1% hydrogel to diverse substrates: human skin (a,b), foam (c), paper (d), wood (e), copper (f), aluminum (g), and glass (h).

The reusability of hydrogel electrodes is a significant challenge in clinical settings due to poor mechanical properties. In addressing this challenge, our developed ion‐driven Ti_3_C_2_T_*x*_–PAM hydrogel exhibits ultrastretchable properties, as visually demonstrated in **Figure** **4**A. This is due to the rich functional groups of Ti_3_C_2_T_*x*_ (—OH, —F, =O),^[^ ^71^ ^]^ which enhance the stretchability and flexibility of the PAM polymeric matrix.

Initially, we determined the optimum concentration of CaCl_2_ for PAM before selecting the final concentration for in situ intercalation with Ti_3_C_2_T_*x*_ nanosheets through a series of stress–strain tests. Pure PAM initially demonstrated a tensile strain of 1520% and a tensile stress of 116 kPa, as shown in Figure 4B (black graph). The performance was enhanced by testing various concentrations of CaCl_2_, as shown in Figure S12, Supporting Information. When the hydrogel was doped with 0.1 m CaCl_2_, the tensile strain was 1926% with a stress of 141 kPa. As the concentration is increased to 0.5 m CaCl_2_, the tensile strain increases to 2538% and the stress reaches 140 kPa. However, further increasing the concentration to 1 m CaCl_2_, the tensile strain decreased to ≈2305%, while the stress increased to 180 kPa, due to over‐crosslinking.

We also investigated the effect of different concentrations of Ti_3_C_2_T_*x*_ nanosheets (0.5%, 1%, and 1.5%) on the optimized 0.5 m CaCl_2_–PAM hydrogels, as shown in Figure 4B, red graph. Incorporation of 0.5% Ti_3_C_2_T_*x*_ nanosheets increases the flexibility of the hydrogel, up to a strain of ≈2617% at a stress level of 144 kPa, as shown in (Figure 4B, blue graph). However, at the concentration of 1% Ti_3_C_2_T_*x*_, ion‐driven Ti_3_C_2_T_*x*_–PAM hydrogel demonstrated a 190 kPa stress with exceptional strain of 2920%, as shown in (Figure 4B, green graph). Increasing the concentration to 1.5% Ti_3_C_2_T_*x*_, the tensile strain decreased significantly to around 2227% at a stress of 169 kPa, as indicated by the pink graph in Figure 4B, implying excessive crosslinking reduces flexibility. The 0.5 m CaCl_2_–MX (Ti_3_C_2_T_*x*_) hydrogels with 1% Ti_3_C_2_T_*x*_ exhibited the outstanding mechanical properties among all concentrations investigated.

Figure 4C illustrates fracture strain and toughness of various hydrogel samples: S‐1 (PAM), S‐2 (PAM–0.1 m CaCl_2_), S‐3 (PAM–0.5 m CaCl_2_), S‐4 (PAM–1 m CaCl_2_), S‐5 (PAM–0.5 m CaCl_2_–MX (Ti_3_C_2_T_*x*_) 0.5%), S‐6 (PAM–0.5 m CaCl_2_–MX 1%), and S‐7 (PAM–0.5 m CaCl_2_–MX 1.5%). Fracture strain is a very crucial property of hydrogels in wearable ECG applications. It can be defined as the values that describe the highest deformation a material can sustain before it fractures.^[^ ^72^ ^]^ In our investigation, sample S‐6 (PAM–0.5 _M_ CaCl_2_–MX 1%) exhibited the highest fracture strain of 2920%, indicating superior stretchability and mechanical stability. The toughness, or the energy absorbed per unit volume to fracture, was also calculated, and the sample S‐6 (PAM–0.5 _M_ CaCl_2_–MX 1%) demonstrated 2550 kJ m^−3^. All measurements were investigated a minimum of 3 times to establish the consistency in the data. Finally, Figure 4D and Table S6, Supporting Information, illustrate the mechanical performance of the hydrogel in comparison with the literature, describing the superior mechanical properties of the ion‐driven Ti_3_C_2_T_*x*_–PAM hydrogel for long‐term physiological monitoring.

The cyclic tensile test for PAM–0.5 _M_ CaCl_2_–MX 1% was conducted to evaluate its hysteresis and elasticity, as depicted in Figure 4E. PAM–0.5 _M_ CaCl_2_–MX 1% hydrogel was stretched for five cycles under various strains (10%, 25%, 50%, 100%, 150%, and 300%). It exhibited a small hysteresis loop and residual strain during the cycles, indicating its excellent elasticity. Additionally, the fatigue resistance required to endure cyclic actuation in reusable ECG electrodes was also tested through 100 repeated loading–unloading cycles at strains of 50%, 100%, and 300%. Measured recovery ratios were 96.76%, 92.05%, and 91.24%, respectively, as shown in Figure 4F. These results demonstrate that the ion‐driven Ti_3_C_2_T_*x*_–PAM hydrogel is a promising candidate for long‐term, reusable ECG electrodes.

Traditional hydrogels tend to break under low compression due to reduced mobility of chain entanglements and nonhomogeneous distribution of stress. We conducted compression stress–strain measurement of PAM, PAM–0.5 m CaCl_2_, and PAM–0.5 m CaCl_2_–MX 1%, as indicated in Figure 4G. The PAM hydrogel exhibited lower compressive strength; however, the addition of Ti_3_C_2_T_*x*_ significantly enhanced the mechanical performance of the hydrogel. The PAM–0.5 m CaCl_2_ exhibited medium performance, which was influenced by ionic interactions but to a lesser extent compared to that of the PAM–0.5 m CaCl_2_–MX 1% hydrogel. All three materials exhibited a nonlinear increase in compressive stress with increasing strain; however, the PAM–0.5 m CaCl_2_–MX 1% hydrogel had a more curved trend, indicating higher energy absorption and deformation resistance. The high compressive strength of PAM–0.5 m CaCl_2_–MX 1% hydrogel makes it suitable for applications requiring toughness and compression resistance. Moreover, the hydrogel was further tested under cyclic compression with varying strains (10%, 15%, 20%, 25%, 30%, 35%, 40%, and 45%), as highlighted in Figure 4H, exhibiting greater stability, enhanced energy absorption, and excellent deformation resistance.

In addition, the recyclability of the ECG electrodes was examined by subjecting the PAM–0.5 m CaCl2–MX 1% hydrogel to 1000 cycles of compressive stress under a strain of 25%, as shown in Figure 4I. A cumulative effect of residual strains was observed with increasing cycle numbers; however, the hydrogel withstands over 1000 loading–unloading cycles without breaking, indicating its excellent stability for long‐term ECG monitoring.

Adhesion is a critical property of hydrogels, which enhances the skin material interface for the acquisition of high‐resolution physiological signals. To test the adhesion properties of PAM–0.5 m CaCl_2_–MX 1% hydrogels, lap‐shear testing was employed, as illustrated in Figure 4J. Several substrates (Figure 4K) were tested, including plastic (69 kPa), paper (115 kPa), glass (31 kPa), iron (77 kPa), aluminum (78 kPa), and tissue paper (66 kPa), demonstrating the excellent adhesion due to the supramolecular chemistry of polymers and the hydrophilicity of glycerol and Ti_3_C_2_T_*x*_. Moreover, the physical adhesion was also investigated and illustrated in Figure 4L (a–h), where PAM‐0.5 _M_ CaCl_2_–MX 1% attached with different substrates, including human skin (a,b), foam (c), paper (d), wood (e), copper (f), aluminum (g), and glass (h). The robust adhesion of this hydrogel to the human body and different surfaces highlights its multimodal use in health monitoring devices.

The adaptive strain behavior of conductive hydrogels offers a significant advantage for long‐term ECG monitoring, due to stable mechanical properties and low strain sensing. This stability maintains consistent skin contact and minimizes motion artefacts with reduced unwanted noise, ensuring reliable signal acquisition. Their mechanical strength and tensile resistance make wearable electrode design easy, enabling them to withstand long‐term use without degradation.^[^ ^73^ ^]^ These attributes make adaptive strain hydrogels promising prospects for long‐term ECG monitoring.

**Figure 5:**
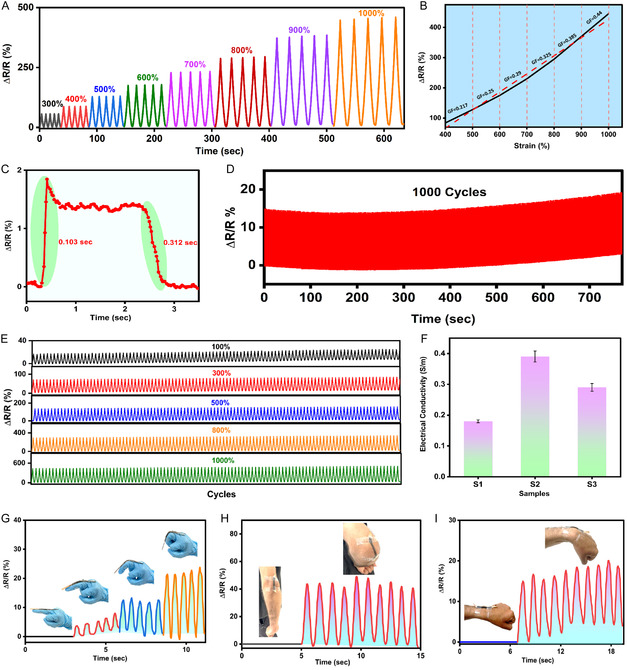
Strain performance characterization of PAM, PAM–CaCl_2_, and ion‐driven Ti_3_C_2_T_*x*_–PAM hydrogel. A) Relative resistance changes (Δ*R*/*R*_0_) in ion‐driven Ti_3_C_2_T_*x*_–PAM hydrogel over a strain range of 300–1000%. B) Gauge factors and relative resistance changes (Δ*R*/*R*_0_) in ion‐driven Ti_3_C_2_T_*x*_–PAM hydrogel at 400–1000% strain. C) Response and recovery time for the ion‐driven Ti_3_C_2_T_*x*_–PAM hydrogel at 25% strain. D) Stability characterization through relative resistance variations (Δ*R*/*R*_0_) in ion‐driven Ti_3_C_2_T_*x*_–PAM hydrogel when stretched to 100%, across 1000 consecutive cycles. E) Stability characterization through relative resistance variations (Δ*R*/*R*_0_) in ion‐driven Ti_3_C_2_T_*x*_–PAM hydrogel when stretched to 100%, 300%, 500%, 800%, and 1000% strain across 100 consecutive cycles. F) Conductivity measurements for hydrogel samples: S‐1 (PAM + 0.5 _M_ CaCl_2_ + MX 0.5%), S‐2 (PAM + 0.5 _M_ CaCl_2_ + MX 1%), and S‐3 (PAM + 0.5 _M_ CaCl_2_ + MX 1.5%). Error bars represent mean ± SD (*n* = 3). G) Real‐time monitoring of relative resistance changes (Δ*R*/*R*_0_) in ion‐driven Ti_3_C_2_T_*x*_–PAM hydrogel during finger bending at various angles. H) Real‐time monitoring of relative resistance changes (Δ*R*/*R*_0_) in ion‐driven Ti_3_C_2_T_*x*_–PAM hydrogel during elbow bending at various angles. I) Real‐time relative resistance changes (Δ*R*/*R*_0_) in ion‐driven Ti_3_C_2_T_*x*_–PAM hydrogel are monitored during wrist bending at various angles.

To evaluate the adaptive sensing ability of the ion‐driven Ti_3_C_2_T_*x*_–PAM hydrogel, tensile strain behavior was investigated under controlled conditions (**Figure** **5**A and S13, Supporting Information). Tensile tests were conducted at a rate of 100 mm min^−1^ for strains ranging from 10% to 300%, and at a rate of 300 mm min^−1^ for strains from 300% to 1000%. The hydrogel exhibited a linear and stable response over the entire range of strains with gauge factors 0.103–0.19 (Figure S14, Supporting Information) between 10% and 300% strain and 0.217–0.44 (Figure 5B) between 400% and 1000% strain. In addition, temperature‐sensitive strain sensing by Ti_3_C_2_T_*x*_–PAM hydrogel is demonstrated in (Figure S15, Supporting Information). The hydrogel demonstrated stable but higher sensitivity at −20 °C than at 0 °C, with Δ*R* of ≈30% and Δ*R* of ≈15%, respectively. This increment suggests that at −20 °C, partial freezing of water within the hydrogel leads to heterogeneous phase separation, in which only confined water domains remain unfrozen. These confined water domains provide highly concentrated ionic regions and fragile percolation networks. Under strain, the discontinuous conductive pathways are easily broken, resulting in a larger relative resistance change, even though the ionic conductivity is lower. In contrast, at 0 °C, the hydrogel remains mostly in a liquid‐like state, with more homogeneous ionic channels and resilient MXene networks, resulting in less variation in resistance upon strain. A similar phenomenon has been observed in antifreezing hydrogels and their MXene‐based composites. At 40 °C, the change in resistance was ≈50% due to the higher mobility of ions.^[^ ^74^, ^75^, ^76^ ^]^ This confirms its stable performance in extensive thermal environments, making it ideal for wearable ECG monitoring. The hydrogel exhibits a rapid response time (0.103 s) and recovery time (0.312 s), as shown in Figure 5C, due to the flexible nature of ion‐driven Ti_3_C_2_T_*x*_ in the PAM matrix.

Long‐term durability was assessed by cyclic loading tests, a crucial parameter for wearable applications. The hydrogel exhibited excellent performance over 1000 consecutive cycles at 100% strain (Figure 5D) and 100 repeated cycles of stretching/releasing at strains of 100%, 300%, 500%, 800%, and 1000% (Figure 5E), demonstrating excellent reliability and fatigue resistance. The hydrogel displayed excellent electrical stability, as supported by an LED indicator test (Figure S16, Supporting Information). Under stretching, the LEDs’ brightness remained constant, indicating that the hydrogel is conductive when deformed, a very desirable property for strain‐adaptive sensing.

Electrical conductivity measurements (Figure 5F) were performed on samples with varying concentrations of MX (Ti_3_C_2_T_*x*_): S1 (PAM–0.5 m CaCl_2_–MX 0.5%), S2 (PAM–0.5 m CaCl_2_–MX 1%), and S3 (PAM–0.5 m CaCl_2_–MX 1.5%) with corresponding conductivities of 0.18 ± 0.0048, 0.39033 ± 0.01762, and 0.29017 ± 0.01265 S m^−1^, respectively. The slight decrease at 1.5% Ti_3_C_2_T_*x*_ is attributed to excessive crosslinking, which restricts ion mobility. These results confirm that 1% Ti_3_C_2_T_*x*_ loading provides the optimal balance between conductivity and mechanical flexibility.

The ion‐driven Ti_3_C_2_T_*x*_–PAM hydrogel exhibits enhanced adhesion and adaptive sensing characteristics, allowing it to conformably adhere to various regions of the human body, such as fingers (Figure 5G), elbows (Figure 5H), and wrists (Figure 5I), for real‐time monitoring of wearable strain.

**Figure 6:**
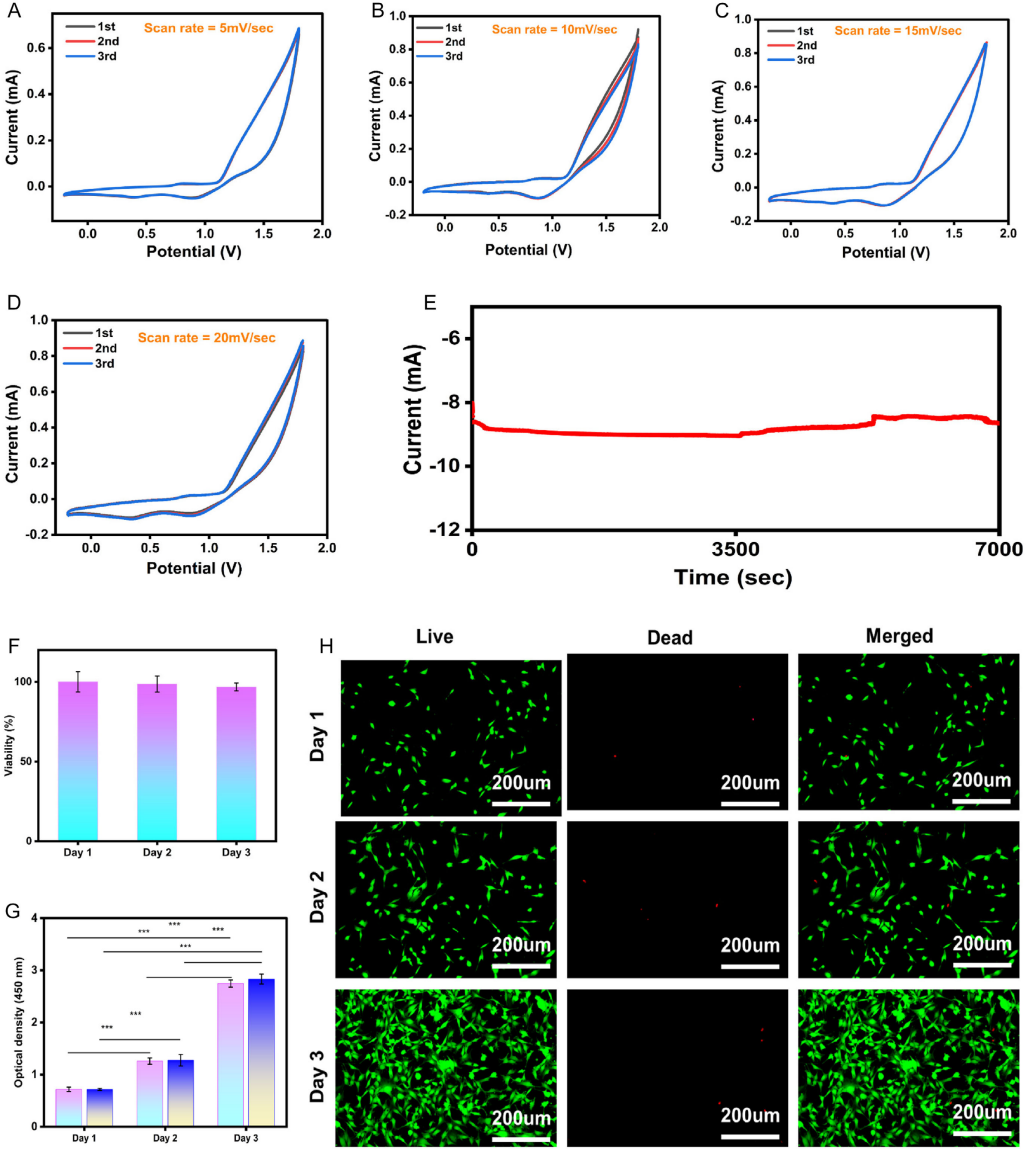
Electrochemical and biocompatibility characterization of PAM, PAM–CaCl_2_, and ion‐driven Ti_3_C_2_T_*x*_–PAM hydrogel. A) Cyclic voltammograms (CVs) of ion‐driven Ti_3_C_2_T_*x*_–PAM hydrogel at a scan rate of 5 mV s^−1^. B) CVs of ion‐driven Ti_3_C_2_T_*x*_–PAM hydrogel at a scan rate of 10 mV s^−1^. C) CVs of ion‐driven Ti_3_C_2_T_*x*_–PAM hydrogel at a scan rate of 15 mV s^−1^. D) CVs of ion‐driven Ti_3_C_2_T_*x*_–PAM hydrogel at a scan rate of 20 mV s^−1^. E) Chronoamperometric analysis of ion‐driven Ti_3_C_2_T_*x*_–PAM hydrogel over 8 h to check the stability in PBS solution. F) Cell viability of ion‐driven Ti_3_C_2_T_*x*_–PAM hydrogel. G) Optical density graph of ion‐driven Ti_3_C_2_T_*x*_–PAM hydrogel. H) Fluorescence cell viability of ion‐driven Ti_3_C_2_T_*x*_–PAM hydrogel. The cell viability and optical density results were expressed as mean ± standard deviation from three independent experiments. Statistical analysis was performed using Student's *t*‐test to evaluate associations between variables. Statistical significance was defined as follows: *p < 0.01, **p < 0.001, ***p < 0.0001, and NS = no significance.

The electrochemical properties are crucial parameters for understanding the mechanical and electrical trade‐off between polymers and conductive agents, which are key factors in acquiring high‐resolution physiological signals.^[^ ^77^ ^]^ In this context, we employed a three‐electrode system to investigate the cyclic voltammetry (CV) of ion‐driven Ti_3_C_2_T_*x*_–PAM hydrogel under different scan rates (5, 10, 15, and 20 mV s^−1^) as shown in **Figure** **6**A–D.

Figure 6A illustrates the CV curve of ion‐driven Ti_3_C_2_T_*x*_–PAM hydrogel at a scan rate of 5 mV s^−1^, which demonstrates a wide potential window (−0.2 to 1.8 V) dominated by capacitive behavior. Higher current response with consecutive increasing cycles indicates the effective storage and rapid distribution^[^ ^78^ ^]^ of Ca^+2^ and Cl^−^ ions within the polymeric matrix. However, at 10 mV s^−1^ (Figure 6B), a sharp negative current with 0.9 mA peak was observed, which denotes the redox process due to ultrafast ion diffusion kinetics.^[^ ^79^, ^80^ ^]^ Increasing the scan rate to 15 mV s^−1^ (Figure 6C) and 20 mV s^−1^ (Figure 6D) did not result in any notable change, indicating stable electrochemical reversibility. This stability is attributed to the synergistic effect of Ca^2+^ intercalation and Cl^−^ electrostatic repulsion, which form robust and dynamic ionic channels within the Ti_3_C_2_T_*x*_ network, enabling efficient charge transfer even under high scan rate conditions.^[^ ^81^, ^82^ ^]^ This performance demonstrates the ion‐driven Ti_3_C_2_T_*x*_–PAM hydrogel's ability for stable electrochemical performance under fast scanning, an essential requirement for real‐time bioelectronics devices. The long‐term electrochemical stability was investigated using chronoamperometric analysis (Figure 6E), which displayed a stable current response of approximately −1.0 to −0.8 mA for 8 h, without significant current oscillation due to the intercalation of ions in Ti_3_C_2_T_*x*_ nanosheets.^[^ ^82^ ^]^ This high stability demonstrates that the ion‐driven Ti_3_C_2_T_*x*_–PAM hydrogel is compatible with long‐term physiological monitoring. Interestingly, the electrochemical stability of the hydrogel was maintained in a phosphate‐buffered saline (PBS) solution, which simulates the ionic environment of human skin, thereby ensuring its wearability and comfort.

In summary, the electrochemical characterization confirms that the ultrastretchable, ion‐driven Ti_3_C_2_T_*x*_–PAM hydrogel exhibits remarkable stability, a wide range of potential windows, and rapid ion transport accompanied by long‐term operational reliability. The reproducible CV response across various cycles with different scan rates, as well as the steady state chronoamperometric response, makes it a potential wearable hydrogel electrode for cardiovascular monitoring.

The biocompatibility of the ion‐driven Ti_3_C_2_T_*x*_–PAM hydrogel was evaluated using a cell viability assay with NIH‐3T3 fibroblast cells, as shown in Figure 6F–H. Fluorescence microscopy images revealed predominantly green fluorescence, indicating viable cells, while red fluorescence, representing dead cells, was minimal compared to the control (Figure S17, Supporting Information). Quantitative analysis demonstrated consistently high cell viability (≈100%) over three consecutive days (Figure 6F), confirming the hydrogel's excellent cytocompatibility.

Further evidence of biocompatibility was provided by the steady increase in optical density (OD) values over time (Figure 6G), suggesting that the hydrogel extract medium does not inhibit cell proliferation. Statistical differences in OD values for day 1, day 2, and day 3 (*p* < 0.001) were observed, as determined by statistical analysis, indicating the reproducibility and reliability of the results. Additionally, live/dead staining (Figure 6H) confirmed that most cells remained viable throughout the culture period, with very few cytotoxic effects observed. In conclusion, the ion‐driven Ti_3_C_2_T_*x*_–PAM hydrogel exhibits consistent cell viability, with promising cell survival and proliferation, thereby addressing a significant challenge in biomaterials for wearable bioelectronic devices.

### 2.5 Application of Ion‐Driven Ti_3_C_2_T_*x*_–PAM Hydrogel Electrodes for Real‐Time Monitoring of Human ECG Signals

**Figure 7:**
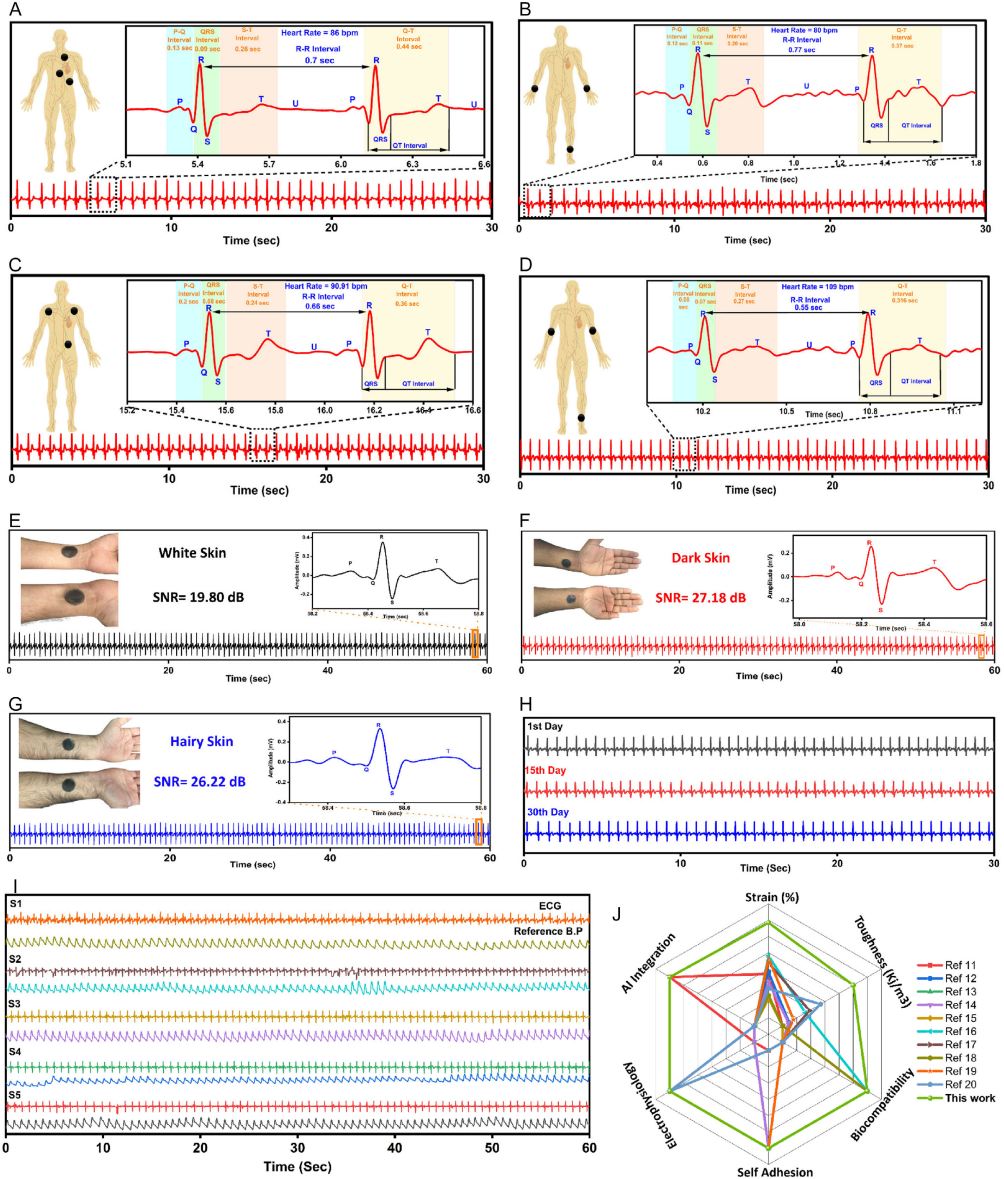
Ion‐driven Ti_3_C_2_T_*x*_–PAM hydrogel electrodes for human ECG signal monitoring. A) High‐resolution ECG monitoring from the heart region, showing intervals: P–Q (0.13 s), QRS (0.09 s), S–T (0.26 s), R–R (0.7 s), and Q–T (0.44 s). B) High‐resolution ECG monitoring from the wrist, displaying intervals: P–Q (0.12 s), QRS (0.11 s), S–T (0.20 s), R–R (0.77 s), and Q–T (0.37 s) at 80 bpm. C) High‐resolution ECG monitoring from the chest area, indicating intervals: P–Q (0.2 s), QRS (0.08 s), S–T (0.24 s), R–R (0.66 s), and Q–T (0.38 s) at 90.91 bpm. D) High‐resolution ECG monitoring from the hinge region, noting intervals: P–Q (0.08 s), QRS (0.07 s), S–T (0.27 s), R–R (0.65 s), and Q–T (0.316 s) at 92.3 bpm. E) ECG monitoring on white skin resulted in a SNR of 19.80 dB. F) ECG monitoring on dark skin, achieving an SNR of 27.18 dB. G) ECG monitoring on hairy skin, with an SNR of 26.22 dB. H) ECG signal monitoring using ion‐driven Ti_3_C_2_T_*x*_–PAM hydrogel electrodes on the 1st, 15th, and 30th days to analyze electrode stability. I) Collection of high‐resolution ECG data from healthy subjects alongside reference BP data for AI‐enabled BP prediction. J) Overall comparison radar of ion‐driven Ti_3_C_2_T_*x*_–PAM hydrogel with literature; references are listed in the Supporting Information.

The ion‐driven Ti_3_C_2_T_*x*_–PAM hydrogel electrodes, which demonstrated outstanding electrochemical sensitivity, superior flexibility, enhanced conductivity, and excellent adaptability, were further applied to human subjects to monitor real‐time ECG signals, as shown in **Figure** **7**.

The ECG is a crucial cardiac parameter that generally consists of the P wave, QRS complex, and T wave. It provides vital information about heart rate, BP, and heart rhythm, which are key indicators for assessing cardiovascular health.^[^ ^83^ ^]^ In conventional ECG setups, there are significant challenges, including dry electrode skin issues, complex setups, a lack of standardized functional representation, and noisy signals. Moreover, the additional treatments of alcohol and spray swabs on the skin can be irritating and time‐consuming.^[^ ^84^, ^85^ ^]^

To address these issues, we applied our ion‐driven Ti_3_C_2_T_*x*_–PAM hydrogel electrodes directly on the skin without using sprays or alcohol. The ion‐driven Ti_3_C_2_T_*x*_–PAM hydrogel electrodes with a size of 1.5 mm thickness and 25 mm diameter were positioned on the healthy subjects with normal BP and an approximate age of 28 years, as illustrated in Figure 7A–D. Figure 7A demonstrates the ECG signal acquisition from the heart region, which resulted in the precise identification of P, Q, R, S, and T waves. The measured intervals were P–Q (0.13 s), QRS (0.09 s), S–T (0.26 s), R–R (0.7 s), and Q–T (0.44 s), corresponding to a heart rate of 86 beats per minute (bpm), which is a reference value from standard ECG measurements of the heart position.^[^ ^86^ ^]^ Heart rate was calculated from the RR intervals, verified with the standard heart rate (60–100 bpm). Figure 7B presents the exceptionally stable ECG signal of the wrist region, with accurate P–Q (0.12 s), QRS (0.11 s), S–T (0.20 s), R–R (0.77 s), and Q–T (0.37 s) intervals at 80 bpm. Figure 7C highlights the high‐resolution ECG taken from the chest area with P–Q (0.2 s), QRS (0.08 s), S–T (0.24 s), R–R (0.66 s), and Q–T (0.38 s) intervals at 90.91 bpm. Simultaneously, Figure 7D illustrates the high‐resolution ECG of the hinge region with P–Q (0.08 s), QRS (0.07 s), S–T (0.27 s), R–R (0.65 s), and Q–T (0.316 s) intervals at 92.3 bpm.^[^ ^86^, ^87^ ^]^ Results from the various locations validate that the ion‐driven Ti_3_C_2_T_*x*_–PAM hydrogel is biocompatible in all body areas examined and is an optimal option for long‐term ECG recording.

Skin–electrode impedance has a significant impact on the quality of electrophysiological signals. The signal‐to‐noise ratio (SNR) is a critical parameter that ensures the signal is less prone to noise from the outside environment or other physiological signals. The quality of the signal deteriorates as the impedance at the skin–electrode interface is high and varies at different electrode locations. Therefore, the choice of an electrode material that minimizes skin–electrode impedance is crucial for current transfer and signal quality. Hair density, skin color, and moisture content in the skin have profound impacts on signal quality.^[^ ^88^, ^89^ ^]^ To investigate the performance of the ion‐driven Ti_3_C_2_T_*x*_–PAM hydrogel, we placed electrodes on various skin types for the acquisition of a high‐resolution ECG signal, such as white skin (Figure 7E), dark skin (Figure 7F), and hairy skin (Figure 7G), using SNR as the crucial parameter. The ion‐driven Ti_3_C_2_T_*x*_–PAM hydrogel electrodes captured high‐fidelity ECG signals, comparable to those of commercial electrodes,^[^ ^90^ ^]^ which typically reduce skin impedance by using an alcohol swab on the skin. Specifically, white skin demonstrated an SNR of 19.80 dB (Figure 7E) with distinct P, Q, R, S, and T peaks. The dark skin resulted in a 27.18 dB SNR (Figure 7F), and the hairy skin closely trailed with a 26.22 dB SNR (Figure 7G). The experiment confirms the impedance stability of ECG signal quality across different skin types, providing proof of the adaptability of ion‐driven Ti_3_C_2_T_*x*_–PAM hydrogel electrodes in ECG acquisition.

Long‐term stability and reusability are crucial properties for wet gel ECG electrodes. To investigate these properties, ion‐driven Ti_3_C_2_T_*x*_–PAM hydrogel electrodes were placed on the subjects’ skin on different days (1st day, 15th day, and 30th day). The developed electrodes demonstrated high‐resolution ECG and exhibited all necessary features, including P wave, QRS waves, and T waves, as highlighted in Figure 7H. This sustained performance over different days highlights the ultramechanical stability and conductivity of ion‐driven Ti_3_C_2_T_*x*_–PAM hydrogel electrodes. Moreover, Figure 7I illustrates the potential for obtaining high‐resolution ECG data of other subjects along with corresponding BP measurements, enabling AI‐based prediction of BP. With this potential, ion‐driven Ti_3_C_2_T_*x*_–PAM hydrogel electrodes can be integrated into various medical wearable devices for both diagnostic and therapeutic purposes. This flexibility and non‐invasive nature of this technology open doors to a wide range of novel medical applications, such as integrating real‐time health diagnostics and monitoring into mobile health management systems. Ultrastretchable ion‐driven Ti_3_C_2_T_*x*_–PAM hydrogel electrodes hold great promise for next‐generation wearable physiological monitoring devices. Performance benchmarking (Figure 7J and Table S7, Supporting Information) against multiple reference materials demonstrates high strain tolerance, AI integration capacity, and adhesion, showcasing a highly versatile material for multimodal applications.

### 2.6 Blood Pressure Prediction via CNN–BIGRU Model Using High‐Resolution ECG Data

**Figure 8:**
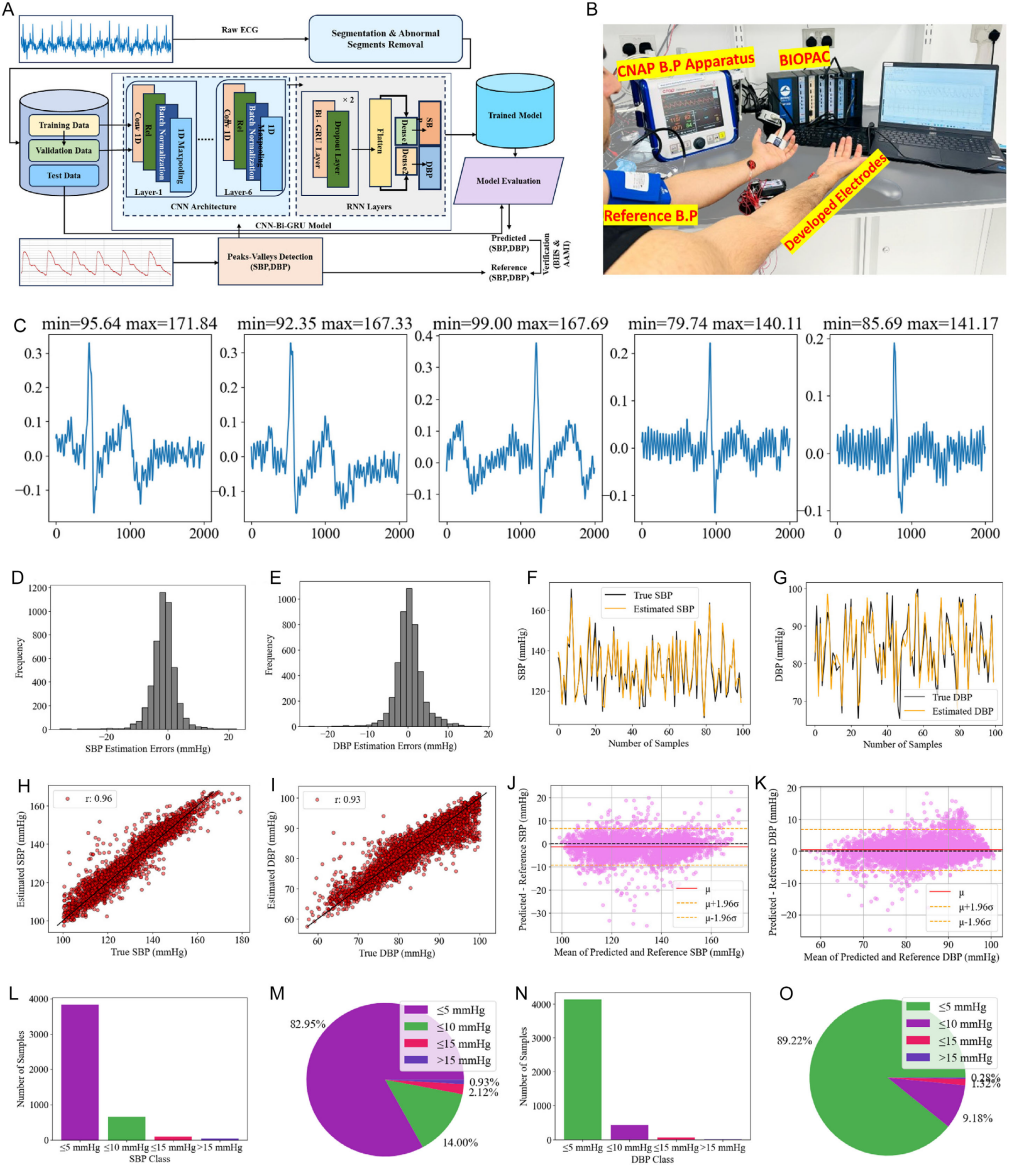
Blood pressure estimation using the CNN–BiGRU model from high‐resolution ECG data. A) Diagram of the CNN–BiGRU model's architecture for predicting BP. B) Diagram of ECG data collection setup with reference BP recording. C) Preprocessing of ECG data. D) Histograms representing the distribution of SBP estimation errors in mmHg. E) Histograms of DBP estimation error distribution in mmHg. F) Comparison plot of reference versus predicted SBP values for 100 ECG data segments (*n* = 100). G) Comparison plot of reference versus predicted DBP values for 100 ECG data segments (*n* = 100). H) The SBP correlation plot displays the Pearson correlation coefficient between the reference and predicted values. I) DBP correlation plot showing the Pearson correlation coefficient between reference and predicted values. J) Bland–Altman plot for SBP in ECG dataset, with ME as a central red line and limits of agreement as dashed orange lines. K) Bland–Altman plot for DBP using the ECG dataset, where the ME is indicated by a red central line and the limits of agreement by dashed orange lines. L–O) Bar and pie charts categorizing the prediction errors for SBP and DBP according to the BHS criteria using the ECG dataset.

This section demonstrates the integration of a hybrid deep learning (DL) approach (Figure S18, Supporting Information) that combines CNN–BiGRU architectures for estimating SBP and DBP from collected ECG signals and reference BP (Figure 7I). The model processes the ECG signal as a singular input to produce outputs for SBP and DBP values simultaneously. The framework architecture is demonstrated in **Figure** **8**A. It begins with six layers of a one‐dimensional CNN (1D‐CNN) to automate the extraction process of unique features from input signals. Each of the CNN layers features a rectified linear unit (ReLU) activation, a batch normalization layer, and a max pooling layer to enhance the learning process and model efficiency. Data from 17 healthy subjects were recorded using our developed ion‐driven Ti_3_C_2_T_*x*_–PAM hydrogel electrodes, a wireless ECG Biopac module, and a Biopac system. For reference, BP, a commercial BP measurement system, the CNAP system, was also employed, as shown in Figure 8B.

The datasets underwent extensive preprocessing, as evident from the raw segments of arterial BP and ECG in Figure 8C. The dataset was segmented into 2000 samples first, corresponding to an interval of one second. The segmentation was irrespective of pulse location markers, such as peaks and valleys. The hybrid CNN–BiGRU model performed well in both training and testing when applied to the recorded ECG dataset for estimating SBP and DBP.

The mean absolute error (MAE), mean error (ME), root mean squared error (RMSE), and standard deviation (STD) for SBP, as listed in **Table** **1**, were 2.91, 1.21, 4.27, 3.03 mmHg, respectively. For DBP, the corresponding values were 2.36, −0.38, 3.31, and 2.33 mmHg. These results demonstrate high accuracy despite the limited data. These results indicate the excellent performance of the suggested model on the ECG dataset, which is particularly significant given the small training dataset comprising only 17 subject records, making this model highly suitable for small datasets.

**Table 1:** Performance evaluation of the proposed hybrid CNN–BiGRU BP estimation framework on ECG datasets.

Technology	BP parameter	MAE [mmHg]	ME [mmHg]	RMSE [mmHg]	STD [mmHg]
ECG	SBP	2.91	1.21	4.27	3.03
	DBP	2.36	–0.38	3.31	2.33

The prediction error histograms of SBP (Figure 8D) and DBP (Figure 8E) from ECG data show that the test errors are normally distributed around the center at zero, with their ranges ranging between [–20, 20] for SBP and [–12, 12] for DBP. Notably, the range of SBP's error distribution is 1.5 times larger than for DBP, which indicates higher variability of SBP values. Moreover, Figure 8F,G illustrates a comparison plot of the reference and predicted BP for SBP and DBP across 100 segments, each with 2000 points. The graphs indicate excellent correlation between the reference and predicted values, SBP in black and DBP in yellow. Pearson's *r* correlation between labeled and predicted BPs of reference SBP (Figure 8H) and DBP (Figure 8I) is a significant linear correlation of 0.96 and 0.93, respectively. The Bland–Altman plots of ground truth versus estimated SBP and DBP values are presented in Figure 8J–K, with agreement intervals for SBP and DBP at [–6.45, 7.05] and [–6.25, 7.14], respectively.

These plots indicate that most of the errors fall within the acceptable range of agreement, i.e., provided by the 95% confidence interval [*μ* − 1.96*σ*, *μ *+ 1.96*σ*], with *μ* as the difference of ME and *σ* as the standard deviation in mmHg. Furthermore, the results indicate the model's capability to provide very accurate estimations of BP across a wide range of SBP and DBP values without significant distortion. The ME values of 1.21 mmHg for ECG–SBP and −0.38 mmHg for ECG–DBP validate the model. The validation of the derived BP estimation model is based on its performance in accordance with internationally recognized standards, such as those established by the British Hypertension Society (BHS) and the Association for the Advancement of Medical Instrumentation (AAMI).

The BHS grading presented in Table S8, Supporting Information, classifies the measurement accuracy into three groups: (A) based on the percentage of measurements (or estimates) in ≤5, ≤10, and ≤15mmHg of the target values on unseen data. Using these standards, our model achieves a grade A rating solely for SBP and DBP estimation from ECG data. A comparative performance analysis of error classification by the hybrid CNN–BiGRU method, based on the BHS standard, is presented in Figure 8L–O and **Table** **2** for both datasets. Additionally, we also adhere to the AAMI standard, where a measurement algorithm is acceptable if the ME of the measurements is less than 5 mmHg and the standard deviation (STD) of errors is less than 8 mmHg for both SBP and DBP, as shown in Table S9, Supporting Information. The evidence in **Table** **3** supports our proposed model as being consistent with the AAMI standard requirements for SBP and DBP on our Center of Cerebro‐Cardiovascular Health Engineering Hong Kong (COCHE) dataset. In summary, the findings show the feasibility and effectiveness of the CNN–BiGRU model for cuffless BP monitoring.

**Table 2:** Performance comparison with BHS standard.

Dataset	BP parameter	≤5 mmHg	≤10 mmHg	≤15 mmHg
(Collected ECG)	SBP	82.95% (A)	96.95% (A)	99.07% (A)
	DBP	89.22% (A)	98.40% (A)	99.72% (A)

**Table 3:** Performance comparison with AAMI standard.

Dataset	BP parameter	ME [mmHg]	STD [mmHg]
(Collected ECG)	SBP	–0.38	3.03
	DBP	1.21	2.33

The performance of the proposed hybrid CNN–BiGRU model is also compared with some of the latest state‐of‐the‐art research methods that have investigated their approaches on ECG signals in relation to the MIMIC and other private databases, as listed in **Table** **4**. As can be observed, most prior work involved complex feature engineering before BP value estimation, which increases system complexity and reduces implementation feasibility on resource‐constrained platforms. On the other hand, our proposed approach does not need feature engineering and the estimation of SBP and DBP can be done concurrently through a single model. This makes computation much cheaper and enhances accuracy in real‐time BP monitoring. Our hybrid CNN–BiGRU approach has also achieved mean estimation accuracies, MAE ± STD of 2.91 ± 3.03 mmHg for SBP and 2.36 ± 2.33 mmHg for DBP, compared to other approaches. These results unveil the efficacy of the proposed approach in nonstop BP monitoring in clinical and nonclinical environments. The proposed approach outperforms previous works, authenticating its clinical utility for noninvasive BP monitoring. The proposed approach gives a cost‐effective solution with reduced sensor units. It improves reliability by eliminating individualized model training, a key limitation for broader deployment. Thus, the suggested framework facilitates continuous BP monitoring, improving early diagnosis and clinical outcomes, such as monitoring in intensive care units and remote health monitoring.

**Table 4:** Performance comparison with literature.

Work	ML/DL model	Database	Performance (MAE ± SD)	
			SBP [mmHg]	DBP [mmHg]
[98]	Complexity analysis + ML	Own dataset (ECG)	7.72 ± 10.22	9.45 ± 10.03
[99]	CNN + LSTM	PulseDB dataset (PPG + ECG)	5.16 ± 7.73	3.24 ± 5.14
[100]	CNN + LSTM	MIMIC‐II (PPG + ECG)	3.97 ± 0.06	2.30 ± 0.19
[101]	CNN‐LSTM	UCI Dataset (PPG + ECG)	5.31 ± 7.25	3.30 ± 4.76
[102]	BiGRU, GRU, attention	MIMIC‐II (PPG)	4.51 ± 7.81	2.6 ± 4.41
[103]	Gaussian process with optimal features decision	MIMIC‐II (PPG + ECG)	7.22 ± 10.79	5.18 ± 8.07
This work	Hybrid DL (CNN‐BiGRU)	(ECG)	2.91 ± 3.03	2.36 ± 2.33

## 3 Conclusion

The ion‐driven Ti_3_C_2_T_*x*_–PAM hydrogel represents a transformative advancement in wearable bioelectronics, delivering a robust, biocompatible, and multifunctional platform for next‐generation cardiovascular monitoring. The hydrogel achieves rapid gelation, ultrastretchability, and high electrical conductivity through an innovative interfacial engineering strategy, ensuring reliable performance under dynamic physiological conditions and enabling the acquisition of high‐fidelity ECG signals. Integrating an AI‐enabled BP prediction framework further enhances its utility, providing accurate and continuous cardiovascular health monitoring. Systematic MD simulations and structure analysis validate the mechanical stability and electrochemical stability of the hydrogel, while in vitro biocompatibility testing confirms its long‐term skin safety. Such data position the ion‐driven Ti_3_C_2_T_*x*_–PAM hydrogel on the horizon of promising materials for next‐generation wearable devices, aiming toward advanced, intelligent healthcare systems with high potential to improve patient outcomes.

## 4 Experimental Section

4.1

4.1.1

##### Materials Acquisition

The MAX phase (Ti_3_AlC_2_, Aladdin 196506‐01‐1), LiF (7789‐24‐4), and APS (A112448) were obtained from Aladdin. Acrylamide (AM, A800656), *N*,*N*′‐methylene bisacrylamide (MBAA, N813086), and calcium chloride dihydrate (CaCl_2_·2H_2_O) were sourced from Macklin. Glycerol (G8190) was procured from Solarbio. The NIH‐3T3 fibroblast cell line, obtained from ATCC, was cultured in Dulbecco's Modified Eagle Medium (DMEM, Gibco, #11875085, USA) with 10% fetal bovine serum (FBS, Gibco, #10270106, USA) and 1% penicillin–streptomycin (Gibco, #15140122, USA). The medium was refreshed every two days. Cells were maintained in a humidified atmosphere with 5% CO_2_ at 37 °C and passaged when they reached 80% confluence. All chemicals and reagents were used as received without further purification.

##### MXene (Ti_3_ *C*_2_ *T*_*x*_ *) Preparation*

MXene (Ti_3_C_2_T_*x*_) (Figure S19, Supporting Information) was synthesized by selectively etching aluminum from Ti_3_AlC_2_ (Figure S20, Supporting Information). In a typical procedure, 1 g of LiF was dissolved in 20 mL of 9 m HCl within a Teflon container. Subsequently, 1 g of Ti_3_AlC_2_ powder was introduced to the mixture and stirred continuously for 24 h at 35 °C. After the reaction slurry was washed multiple times with deionized water and centrifuged at 3500 rpm for 10 min, it was adjusted to a pH of 5.5–6. The resultant Ti_3_C_2_T_*x*_ was then ultrasonicated under argon for 1 h to disperse the sheets uniformly. The suspension was further concentrated by centrifugation at 7000 rpm for 30 min and then dried in a vacuum oven for subsequent use.

##### Intercalation of Ions into MXene (Ti_3_ *C*_2_ *T*_*x*_ *) Nanolayers*

To modify the Ti_3_C_2_T_*x*_ nanosheets, solutions of CaCl_2_·2H_2_O were prepared in three concentrations (0.5, 1, 1.5 m) and stirred for 10 min. Ti_3_C_2_T_*x*_ nanosheets were added to the prepared solutions at varying concentrations (0.5%, 1%, 1.5% w/w), ultrasonicated for 30 min, and dried for 24 h to study the effects of Ca^2+^ and Cl^−^ ions on the Ti_3_C_2_T_*x*_ nanosheets.

##### Hydrogel Preparation

A specific quantity of AM, MBAA, APS, and Glycerol was incorporated into ion‐driven Ti_3_C_2_T_*x*_ to achieve the desired formulation (Table S2, Supporting Information). This was followed by the application of focused UV light to cross‐link the hydrogel within 5 min.

##### Characterizations

Morphological analyses of Ti_3_C_2_T_*x*_ nanosheets and ion‐driven Ti_3_C_2_T_*x*_ nanosheets were conducted using a SEM (Czech TESCAN MIRA LMS) and hydrogel samples with an environmental scanning electron microscope (Philips XL30) at 10 kV in low‐vacuum mode to avoid material degradation. Samples were prepared by mounting on conductive carbon tape on an SEM stub.

The structure and elemental composition of the ion‐driven Ti_3_C_2_T_*x*_ nanosheets nanolayers were analyzed using TEM (JEOL JEM‐F200 (Japan) and a JEM‐F200(URP) TEM for high‐resolution imaging and elemental mapping.

XPS (Thermo Scientific K‐Alpha) was performed using standard protocols, in which the samples were sputtered with a 5 nm gold thickness and affixed to the XPS holder using conductive carbon tape. The scans were taken at 200 eV with a 20 eV resolution.

PerkinElmer FTIR was used in transmission mode to scan the samples from 400 to 4000 cm^−1^ with a 4 cm^−1^ resolution range. A Bruker D2 Phaser XRD was used for structural analysis with scans from 5° to 60° 2*θ* at a rate of 1° per minute. PerkinElmer DSC 8000 was employed for thermal characterizations with a temperature range of 120–800 °C and a heating rate of 20 °C min^−1^. The Ti_3_C_2_T_*x*_ nanosheets and ion‐driven Ti_3_C_2_T_*x*_ colloidal solutions were analyzed by an Agilent CARY 3500 Multicell UV–vis spectrophotometer.

The mechanical and strain properties were characterized using an Instron 5942 microtester at various rates. Initially, different batches of samples, each measuring 20 × 10 × 1.5 mm, were prepared and then subjected to multiple mechanical characterizations, including stress–strain, compression, cyclic strain, and adhesive tests. The error bars were calculated for the accuracy of the data and represent mean ± SD (*n* = 3). However, in strain testing, the resistance was measured using the Keithley DAQ6510 system. The rate of change of relative resistance^[^ ^91^ ^]^ is defined as

(1) $$\frac{\Delta R}{R_{0}}\left(\%\right)=\left(\frac{R-R_{0}\;}{R_{0}}\right)\times 100\;$$

where *R*_0_ is the hydrogel's initial resistance value and *R* is the final hydrogel's resistance value at a specific strain rate.

Thermal strain stability was assessed using an Instron 5942 microtester, with a Keithley DAQ6510 system. The hydrogel samples, measuring 20 × 10 × 1.5 mm, were prepared and placed at –20, 0, and 40 °C for 24 h before testing.

The strain factor (GF) is calculated as:^[^ ^91^ ^]^

(2) $$\text{GF}=\left(\left(\Delta R÷R_{0}\right)/\varepsilon \right)\times 100$$

where *ε* represents the strain

The electrical conductivity of the ion‐driven Ti_3_C_2_T_*x*_–PAM hydrogel was measured using the principle of the two‐probe method. The thickness, length, and width of the hydrogel were measured using a vernier calliper and a micrometre screw gauge. The conductivity formula^[^ ^92^ ^]^ used was

(3) σ = l/R×A

where *σ* is the electrical conductivity, *l* is the length of the ion‐driven Ti_3_C_2_T_*x*_–PAM hydrogel, and *A* represents the area (thickness and width) of the ion‐driven Ti_3_C_2_T_*x*_–PAM hydrogel. At the same time, *R* is the measured resistance of the ion‐driven Ti_3_C_2_T_*x*_–PAM hydrogel, respectively. The error bars were calculated for the accuracy of the data and represent mean ± SD (*n* = 3).

A simple LED circuit was used to demonstrate the conductivity of the hydrogel. The hydrogel was placed between two copper electrodes connected to a power supply and an LED. When intact, the hydrogel completed the circuit and lit the LED. Cutting the hydrogel turned the LED off, indicating loss of conductivity. After self‐healing, the LED lit up again, confirming restoration of the conductive network. The LED remained on even during stretching, indicating that the hydrogel retained conductivity under elongation.

CV was measured to understand the electrochemical behavior of ion‐driven Ti_3_C_2_T_*x*_–PAM hydrogel. Initially, the samples, measuring 2 × 2 cm, were placed on working electrodes and immersed in PBS electrolyte. The Ag/AgCl electrode and the platinum electrode were used as the reference and counter electrode. The potential was scanned from 1.8 to −0.2 V at the scan rate of 5, 10, 15, 20 mV s^−1^. For chronoamperometry, a −1.5 V potential was applied for 8 h.

The biocompatibility of ion‐driven Ti_3_C_2_T_*x*_–PAM hydrogel was characterized by the cell viability method. Initially, the hydrogel samples were sterilized by immersing them in a 75% ethanol solution overnight and then purified with a PBS solution. Additionally, UV light was used to enhance the sterilization. The extraction procedure followed the GB/T 16886.12/ISO 10993‐12 guidelines. The sterilized samples were immersed in DMEM for 24 h at 4 °C, followed by filtration using a 0.2 μm filter and stored at 4 °C for further use. Cells with a density of 2 × 10^3^ cells mL^−1^ per 100 μL of DMEM were seeded into 96‐well plates. The DMEM solution was removed after an overnight incubation period and replaced with 100 μL of the prepared extraction medium.

The Cell Counting Kit‐8 assay was used to assess cell viability over a 3 day period, and the absorbance of the supernatant was measured at 450 nm. The live cells and dead cells were identified by staining the cells with 5 μM Calcein AM (Invitrogen, #C3100MP, USA) at 37 °C for 20 min, followed by staining with 5 μM Propidium Iodide (PI) (Sigma–Aldrich, #81845, USA) at room temperature for 1 min. A Nikon Eclipse Ci, Japan, fluorescent microscope was used for imaging the stained samples, and ImageJ software was employed for processing the fluorescence images.

The results were expressed as mean ± standard deviation from three independent experiments. Statistical analysis was performed using Student's *t*‐test to evaluate associations between variables. Statistical significance was defined as follows: *p < 0.01, **p < 0.001, ***p < 0.0001, and NS = no significance

The application and ECG data were collected using continuous BP monitoring equipment and a computer‐based data acquisition system (NIBP100D, BIOPAC Systems Inc.). Blood pressure and ECG data were recorded simultaneously for all individuals at a sampling rate of 2000 Hz. Data collection for all 17 subjects was conducted in accordance with the Human Ethics guidelines of the Hong Kong Science Park (reference number: HKSTP:2023‐005), and consent was obtained from each participant before data collection. The images presented in the figures feature B.K., W.K., and I.G., who have given their consent for publication.

##### AI Model Training

During model training, the CNN network initiates the process of feature extraction by applying convolution as the primary operation in each layer. Filters, like neurons, process weighted inputs to produce output values. Convolution layers in this process utilize a sequence of learnable filters of sizes 32, 64, 128, 256, 512, and 1024, along with a kernel size of 3 × 1, to effectively extract features from the ECG data. Following convolution, the ReLU activation function introduces nonlinearity, which enables accelerated training. Batch normalization is performed after each convolutional operation to stabilize the training and enhance the model's capacity to generalize. The 1D max pooling layer with a kernel size of 2 × 1 downsamples the complexity of the convoluted feature maps to reduce the size of the feature maps. After the CNN layers, the features are input into two BiGRU layers to predict SBP and DBP values. Both BiGRU layers with 128 and 350 hidden units, respectively, utilize hard‐sigmoid functions as the recurrent activation for the forget, input, and output gates. The BiGRU network, a type of recurrent neural network, addresses the declining gradient issue through memory blocks with trainable gates that determine data relevance during learning. The topology enables the model to capture and process temporal and long‐range interdependencies in time‐series ECG data. Both the input sequence and its reverse are processed within every layer, and in the “concat” merge mode, they are concatenated to preserve rich temporal information.

In the training process, a dropout layer with a factor of 0.2 is inserted after each BiGRU layer, preventing overfitting and making the model more robust and generalizing its performance to new, unseen data. The architecture and prediction capability of the CNN–BiGRU approach are optimized with the aid of a heuristic grid search technique.^[^ ^93^, ^94^ ^]^

This strategic approach in model designing enables it to predict SBP and DBP from the ECG signal with a high output reliability and accuracy. Data segregation was initially performed in 2000‐sample segments, corresponding to a duration of 1 s. This segregation was done irrespective of the position markers of pulses, such as peaks and troughs. The dataset was initially composed of a total of 6025 segments. During preprocessing, “bad” segments were removed, resulting in a clean dataset of 5143 “good” segments. These segments had a mean SBP of 129 mmHg and a mean DBP of 87 mmHg. DBP had a minimum of 57 mmHg and a maximum of 100 mmHg, while SBP had a range from 100 to 179 mmHg. Extensive descriptions of the dataset appear in Table S10–12, Supporting Information.

Moreover, the statistical distribution of the SBP and DBP within our own data house is demonstrated in Figure S21A–B, Supporting Information. The preprocessing tasks played an instrumental role in ascertaining the quality and reliability of data, which enabled us to record several heart cycles over each 2000‐sample record successfully.

The proposed CNN–BiGRU model architecture was implemented and trained under Python 3.9.17, along with Keras 2.10.0 over the TensorFlow 2.10.0 backend. The experiments of training and testing were conducted on a workstation equipped with a NVIDIA GeForce RTX 4080 Laptop GPU, featuring 32 GB of dedicated memory. It comes with a 64‐bit Windows 11 Pro N operating system preinstalled. It is powered by a 13th Generation Intel(R) Core (TM) i9‐13900HX processor, which operates at 2.2 GHz, featuring 24 cores and 32 logical processors, along with 32 GB of RAM.

The data were split into a test set (10%) and a training set (90%). Additionally, a 10% validation set of the training data was established to test the model. This is the typical validation scheme used to select the optimal model based on the performance of the validation data. Precautions were taken to avoid any overlap between the training and test sets, thereby maintaining the integrity of the evaluation process and reducing the risk of bias. For training, the model used a batch size of 64 and a learning rate of 1.0, and early stopping was performed after at most 100 epochs to prevent overfitting. Empirical evidence has shown that the Adadelta optimizer performs better than other optimizers, such as Adam, RMSProp, and gradient descent, in terms of performance and stability. To evaluate the model's performance, we employed various measures, including ME, MAE, RMSE, and STD. MAE is widely used to validate deep learning models, particularly those that address regression problems.^[^ ^86^ ^]^ The model's precision was further evaluated using linear correlation analysis and Bland–Altman plots for the two datasets to assess the concordance between the predicted and reference values on the COCHE datasets, which complied with the guidelines of the BHS and the AAMI.

##### Computational Details

In computational studies, the Ti_3_C_2_T_*x*_ nanosheet structure, with dimensions of 35.15 × 35.15 × 35.15 Å^3^ and angles of 90° × 90° × 120°, was designed using VASP. It was then embedded with 514 water molecules, six glycerol molecules, six PAM chains, and three CaCl_2_ molecules. MD runs were conducted in the NPT ensemble at 298 K and 1 GPa for a time of 5 ns. Following standard procedures, periodic boundary conditions were applied to maintain constant temperature and pressure levels throughout the simulation process.

Interatomic interactions were calculated with a cutoff distance of 12.5 Å for van der Waals, and electrostatic interactions were calculated according to the Ewald summation method. Numerical integration of the equations of motion was performed using the velocity Verlet algorithm with a time step of 1 fs, ensuring both accuracy and stability in the simulation. MD simulations were done on the Large‐scale Atomic/Molecular Massively Parallel Simulator.^[^ ^95^ ^]^ Lennard–Jones (LJ) 12‐6 potential was employed to model van der Waals interactions, while electrostatic interactions were computed by particle–particle–mesh summation with a cutoff of 12.0 Å. Nosé–Hoover algorithm was utilized for controlling temperature and pressure, and the simulation was run at a time step of 1 fs. After energy minimization, the system was gradually increased from 30 to 298 K under an NPT ensemble (constant temperature and pressure) of 1 bar for 100 ps. A subsequent 10 ns NPT simulation was performed under a constant temperature of 298 K.

Density functional theory (DFT) calculations were performed to optimize molecular structures using the B3LYP functional and the 6‐31G(d,p) basis set.^[^ ^96^ ^]^ RDG and electrostatic potential analyses were performed on the DFT‐optimized structures of PAM, CaCl_2_, PAM–CaCl_2_, Ti_3_C_2_T_*x*_, Ti_3_C_2_T_*x*_–CaCl_2_, and ion‐driven Ti_3_C_2_T_*x*_–CaCl_2_ molecules using the Multiwfn package.^[^ ^39^ ^]^ The resulting data were visualized using VMD software.^[^ ^97^ ^]^

## Supporting Information

Supporting Information is available from the Wiley Online Library or from the author.

## Conflict of Interest

The authors declare no conflict of interest.

## Supporting information

Supplementary Material

## Data Availability

The COCHE dataset, which supports the findings of this research, is available from the corresponding author upon reasonable request.
