# Correction to “Ion‐Driven Interfacial Engineering of MXene‐PAM Hydrogels for Advanced Wearable ECG and AI‐Driven Blood Pressure Monitoring”

**DOI:** 10.1002/smsc.70297

**Published:** 2026-05-20

1

Bangul, K., Bilawal, K., Syed, B. A., Waseem, U. K., Junchen, L., Shohidul, I., Iyapan, G., Raffi U. S. A., Mohamed E. H., Bee L. K. Ion‐Driven Interfacial Engineering of MXene‐PAM Hydrogels for Advanced Wearable ECG and AI‐Driven Blood Pressure Monitoring. Small Science. 2026; 6: e202500526. https://doi.org/10.1002/smsc.2025005264

In the originally published version of this article, a notation error appeared in the *y*‐axis labels of the strain‐dependent resistance change graphs. Specifically, the relative resistance change was incorrectly labeled as Δ*R*/*R* instead of the correct notation Δ*R*/*R*_0_. This error occurred in the following figures: Figure 5 in the main manuscript and Supporting Figures 13, 14, and 15 in the Supporting Information. The figures have now been corrected to display the accurate notation Δ*R*/*R*_0_. This correction does not affect any data, results, or conclusions presented in the article.

**FIGURE 5:**
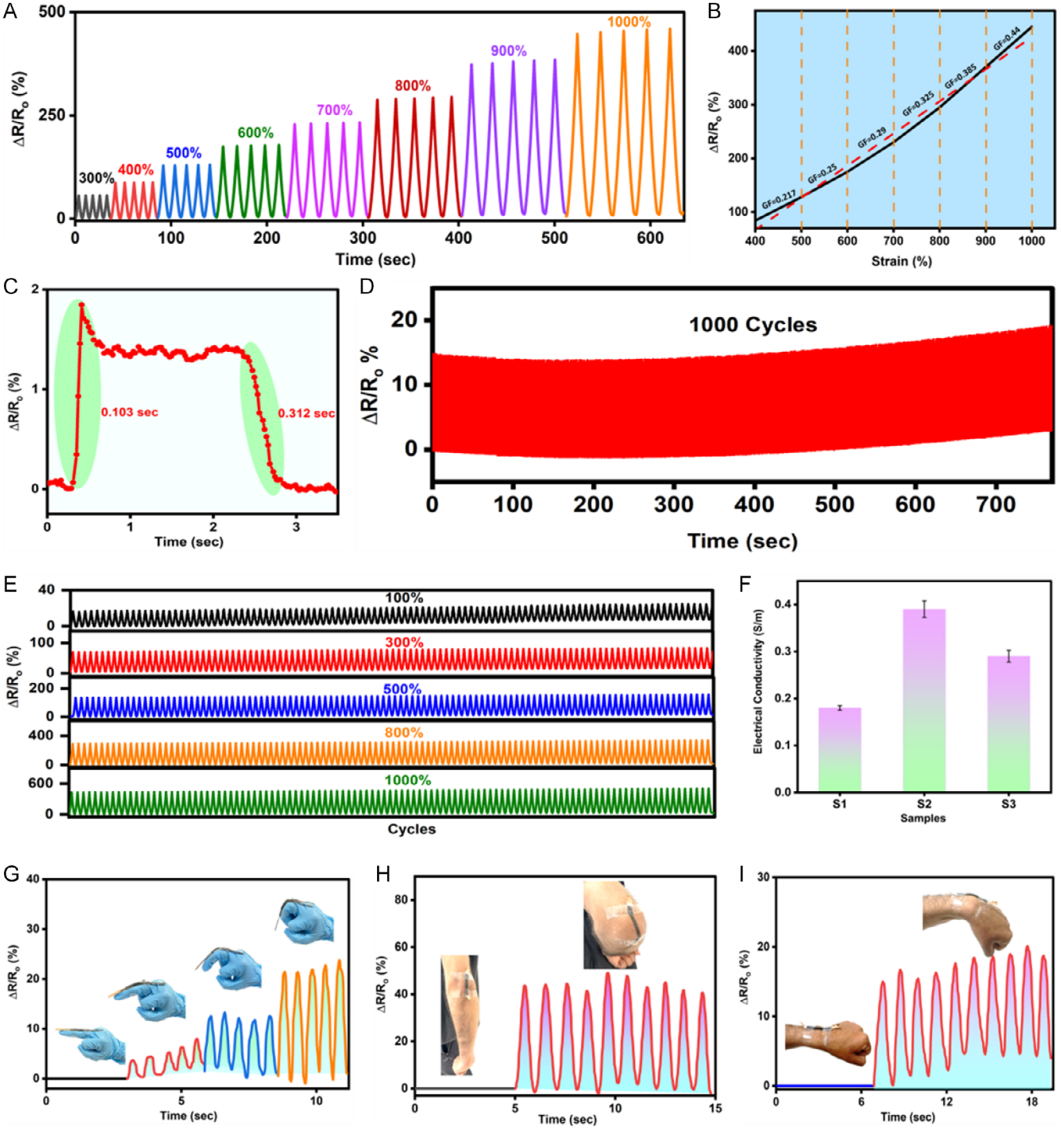
Strain performance characterization of PAM, PAM–CaCl_2_, and ion‐driven Ti_3_C_2_T_*x*_–PAM hydrogel. (A) Relative resistance changes (Δ*R*/*R*_0_) in ion‐driven Ti_3_C_2_T_*x*_–PAM hydrogel over a strain range from 300 to 1000%. (B) Gauge factors and relative resistance changes (Δ*R*/*R*_0_) in ion‐driven Ti_3_C_2_T_*x*_–PAM hydrogel at 400 to 1000% strains. (C) Response and recovery times for the ion‐driven Ti_3_C_2_T_*x*_–PAM hydrogel at 25% strain. (D) Stability characterization through relative resistance variations (Δ*R*/*R*_0_) in ions‐driven Ti_3_C_2_T_*x*_–PAM hydrogel when stretched to 100%, across 1000 consecutive cycles. (E) Stability characterization through relative resistance variations (Δ*R*/*R*_0_) in ion‐driven Ti_3_C_2_T_*x*_–PAM hydrogel when stretched to 100%, 300%, 500%, 800%, and 1000% strain across 100 consecutive cycles. (F) Conductivity measurements for hydrogel samples: S‐1 (PAM + 0.5M CaCl_2_ + MX 0.5%), S‐2 (PAM + 0.5M CaCl_2_ + MX 1%), and S‐3 (PAM + 0.5M CaCl_2_ + MX 1.5%). Error bars represent mean ± SD (*n* = 3). (G) Real‐time monitoring of relative resistance changes (Δ*R*/*R*_0_) in ion‐driven Ti_3_C_2_T_*x*_–PAM hydrogel during finger bending at various angles. (H) Real‐time monitoring of relative resistance changes (Δ*R*/*R*_0_) in ion‐driven Ti_3_C_2_T_*x*_–PAM hydrogel during elbow bending at various angles. (I) Real‐time relative resistance changes (Δ*R*/*R*_0_) in ion‐driven Ti_3_C_2_T_*x*_–PAM hydrogel are monitored during wrist bending at various angles.

**Supporting Figure 13:**
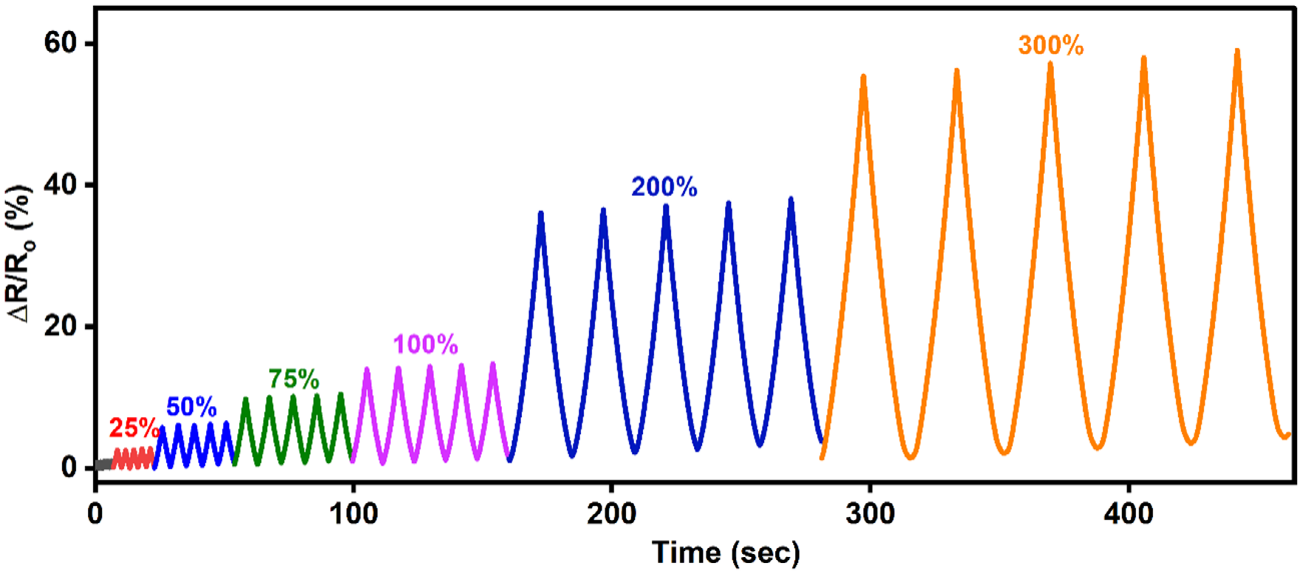
Relative resistance changes (Δ*R*/*R*_0_) in Ions‐driven Ti_3_C_2_T_x_–PAM hydrogel over a 10–300% strain range.

**Supporting Figure 14:**
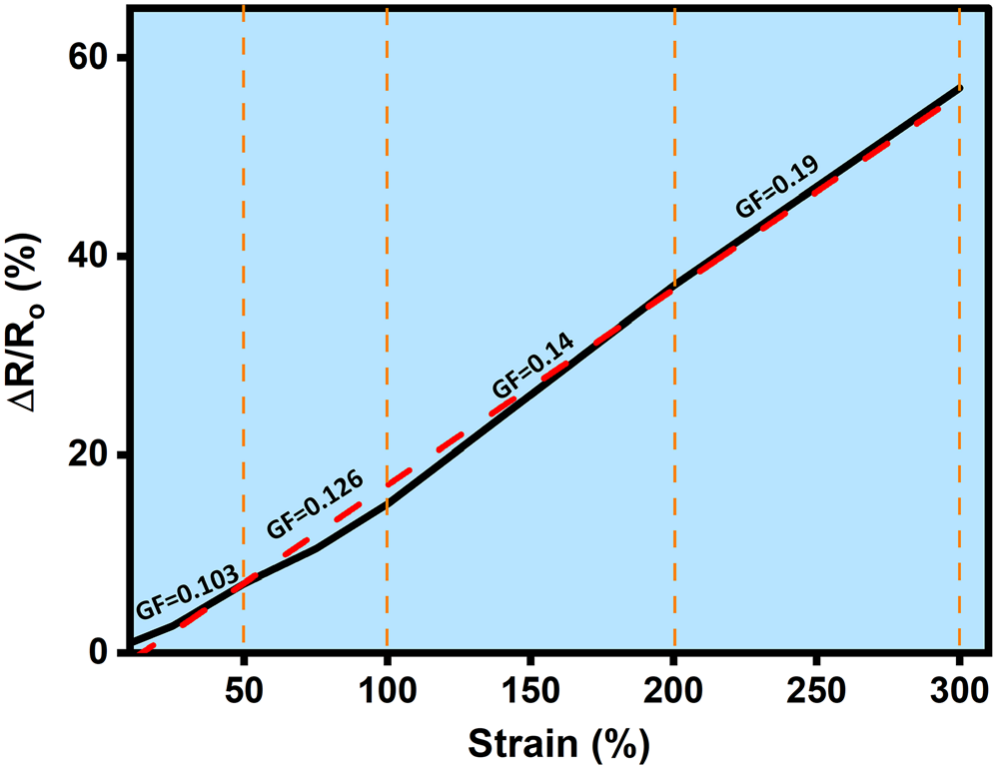
Gauge factors and relative resistance changes (Δ*R*/*R*_0_) in Ions‐driven Ti_3_C_2_T_x_–PAM hydrogel at 10 to 300% strains.

**Supporting Figure 15:**
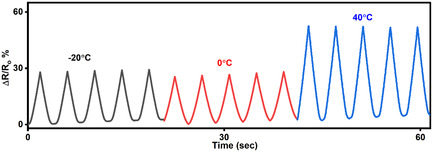
Relative resistance changes (Δ*R*/*R*_0_) in Ions‐driven Ti_3_C_2_T_x_–PAM hydrogel over a 100% strain range at −20°C, 0°C and 40°C.

We apologize for this error.

