# Supplementary material for: Ion‐Driven Interfacial Engineering of MXene–Polyacrylamide Hydrogels for Advanced Wearable Electrocardiography and AI‐Driven Blood Pressure Monitoring
Source: Small Sci. 2026 Mar 28;6(4):e202500526. doi: 10.1002/smsc.202500526 (PMC13154919; doi:10.1002/smsc.202500526)
Supplement: Supplementary file 1 — Supplementary Material [file SMSC-6-e202500526-s001.pdf]

Ion-Driven Interfacial Engineering of MXene-Polyacrylamide Hydrogels for Advanced Wearable Electrocardiography and AI-Driven Blood Pressure Monitoring

Bangul Khan[1,2], Bilawal Khan[3], Syed Bilal Ahmed[3], Wasim Ullah Khan[4], Junchen LIAO[1], Md Shohidul Islam[2], Iyappan Gunasekaran[2], Rafi U Shan Ahmad[1,2], Mohamed Elhousseini Hilal[2]*, Bee Luan KHOO[1,2,5]*

[1]Department of Biomedical Engineering, College of Biomedicine, City University of Hong Kong, Kowloon Tong, 999077, Hong Kong.

[2]Hong Kong Centre of Cerebro-Cardiovascular Health Engineering (COCHE), Shatin, 999077, Hong Kong.

[3]Department of Materials Science and Engineering, City University of Hong Kong, Kowloon Tong ,999077, Hong Kong.

[4]School of Information Engineering, Yango University,350015, Fuzhou, Fujian, China.

[5]Institute of Digital Medicine, City University of Hong Kong, Kowloon Tong, 999077, Hong Kong.

*Correspondence: Bee Luan Khoo, blkhoo@cityu.edu.hk, Mohamed Elhousseini Hilal, melhouss@um.cityu.edu.hk

Supporting Table 1: SEM-EDS elemental weight percentage of ion-driven $Ti_3C_2T_x$

Elements	wt%	wt % Sigma	At%
C	23.55	0.37	44.64
O	10.28	0.41	14.63
F	12.75	0.23	15.28
Cl	0.11	0.02	0.07
Ca	0.43	0.03	0.24
Ti	52.88	0.38	25.13
Total	100.00		100.00

Supporting Table 2: XRD d-Spacing Calculations Using Bragg's Law

Material	2θ (°)	θ (°)	sin(θ)	d-spacing (Å)
Ions-driven-$Ti_3C_2T_x$	6.86	3.430	0.0599	12.86
$Ti_3C_2T_x$	7.46	3.730	0.0651	11.83
MAX Phase	9.69	4.845	0.0845	9.11

Supporting Table 3: XPs deconvoluted peaks of Pristine $Ti_3C_2T_x$ (eV) & Ions-driven $Ti_3C_2T_x$ (eV)

Element	Peak	Pristine $Ti_3C_2T_x$ (eV)	Ion-Driven $Ti_3C_2T_x$
C 1s	C-Ti-Tx	282.0	282.1
	C-C	284.6	285.0
	C-O, CHx	286.8	286.5
Ti 2p	Ti (I, II, or IV)	455.0 & 461.2	454.7 & 460.2
	Ti^{2+} (I, II, or IV)	455.8 & 461.5	455.5 & 461.2
	Ti^{3+} (I, II, or IV)	457.3 & 463.1	457.0 & 462.4
	TiO_2	458.5 & 464.5	458.4 & 464.2
	TiO_2-xFx	459.2	459.5
F 1s	C-Ti-Fx	684.9	685.1
	TiO_2-xFx	685.3	686.2
	Al-Fx	686.4	-
	Al-OFx	687.5	-
O 1s	TiO_2	530.0	529.7
	C-Ti-Ox	531.2	530.9
	C-Ti-(OHx)	532.0	531.8
	Al_2O_3-(OR)	532.9	532.8
	H_2Oads (IV)-OR	533.8	533.5
Ca 2p	Ca $2p_{3/2}$	-	348.5
	Ca $2p_{1/2}$	-	352.0

Supporting Table 4: Detailed Formulation of the samples

S: No	AM	$CaCl_2.2H_2O$	$Ti_3C_2T_x$	H_2O	Glycerol	MBAA	APS
S1	1.8g	-	-	4ml	1ml	0.004g	0.06g
S2	1.8g	0.1 M	-	4ml	1ml	0.004g	0.06g
S3	1.8g	0.5 M	-	4ml	1ml	0.004g	0.06g
S4	1.8g	1 M	-	4ml	1ml	0.004g	0.06g
S5	1.8g	0.5 M	0.009 g	4ml	1ml	0.004g	0.06g
S6	1.8g	0.5 M	0.018g	4ml	1ml	0.004g	0.06g
S7	1.8g	0.5 M	0.025g	4ml	1ml	0.004g	0.06g

AM: Acrylamide, APS: Ammonium persulfate, MBA: N, N'-methylene bisacrylamide

Supporting Table 5: XPS deconvoluted peaks of PAM, PAM-$CaCl_2$ and ion-driven $Ti_3C_2T_x$-PAM (eV)

Element	Peak (eV)	PAM (eV)	PAM-$CaCl_2$ (eV)	Ion-Driven $Ti_3C_2T_x$-PAM (eV)
C1s	C–C	285.0	285.1	285.3
	C–O/C–N	286.2	286.2	286.6
	C=O	288.1	288.6	288.5
	Ti-C	-	-	284.4
O1s	C=O	-	531.9	532.0
	O–H	-	532.8	533.0
N1s	C-N	399.5	400.1	400.5
	N-H_2	400.2	400.2	400.2
Ca 2p	Ca $2p_{3/2}$	-	347.8	Slight shifts
	Ca $2p_{1/2}$	-	351.3	Slight shifts
Ti 2p	Ti2P	-	-	454-460

Supporting Table 6: Comparison of Strain (%) vs Toughness (MJ/m^3) of ion-driven $Ti_3C_2T_x$-PAM hydrogel with the literature

Material	Strain (%)	Toughness (MJ/m^3)	Ref
PAAm-oxCNTs hydrogel	1041	2.29	[1]
PAM/gelatin/ILs/	602.82	0.649	[2]

MXene/glycerol (PGIMG) hydrogel			
Gelatin/PAM	1080	0.16	[3]
Al-TA/GG-PAM	786	0.306	[4]
phenylboronic acid supramolecular hydrogel			
Polyacrylamide hydrogel loaded with copper sulfate and ammonium chloride (PAC),	390	0.08	[5]
Polyacrylamide-chitosan-Al 3+ (PAM-CS-Al 3+) double network (DN) hydrogel	1040	0.73	[6]
PAM-MXene-SF	1560	0.165	[7]
MXene doped PAM–SA	1728	2.6337	[8]
PAM-SA hydrogel	1465	1.3	[9]
PAA/PAM/MXene/TA hydrogel	560.82	0.895	[10]
**Ion-Driven $Ti_3C_2T_x$ Hydrogel**	**2833**	**2.549**	**This work**

Supporting Table 7: Performance Comparison of Ion-driven $Ti_3C_2T_x$-PAM hydrogel with the literature.

**Hydrogel**	**Elongation at break %**	**Toughness (kJ/m^3)**	**Conductivity S/m**	**Gelation time (min)**	**Biocompatibility**	**Self-Adhesion**	**Electrophysiology**	**AI integration**	Ref
PAAM-SA-MXene-	1350	NA	1.6	120	NA	NA	NA	Yes	[11]
TiO2@MXene-PAA	1400%	225	0.1	21	NA	Yes	Yes	No	[12]
C2-PIL/MXene	600%	NA	0.49	NA	Yes	Yes	NA	NA	[13]
gelatin-modified MXene polyacrylamide (PAAm)	1100%	205	0.15	240	NA	Yes	NA	NA	[14]
N-isopropyl acrylamide, PAM, MXene	600%	NA	0.21	10	NA	NA	Yes	NA	[15]
A-PS-I@M hydrogel	1903 %	806	0.148	60	Yes	Yes	Yes	NA	[16]
PAA/SCMC/Ti3C2TX/Sn4+ hydrogel	1688%	950	0.82	NA	NA	Yes	Yes	NA	[17]
TA@MXene	690 %	NA	NA	240	Yes	Yes	Yes	NA	[18]
PU@MXene/PAM Hydrogel	1834%	385.6	0.05	6	NA	Yes	Yes	NA	[19]
PAM/MXene	900%	1340	NA	15	NA	NA	Yes	NA	[20]
**Ions-Driven $Ti_3C_2T_x$-PAM Hydrogel**	**2920%**	**2550**	**0.39**	**5**	**Yes**	**Yes**	**Yes**	**Yes**	**This work**

Supporting Table 8: BHS standard requirements

	Cumulative Percentage Error (SBP, DBP)		
	**≤ 5 mmHg**	**≤ 10 mmHg**	**≤ 15 mmHg**
**Grade A**	60%	85%	95%
**Grade B**	50%	75%	90%
**Grade C**	40%	65%	85%

Supporting Table 9: AAMI standard requirements

Metrics	BP Parameter	AAMI Standards
ME	SBP, DBP	≤ 5 mmHg
STD		≤ 8 mmHg

Supporting Table 10: Subjects' general information

Subject Information	Min	Max
Age (year)	24	35
Hight (cm)	160	184
Weight (kg)	46	83
Gender	14-Male	3-Female

Supporting Table 11: Statistics of reference DBP and SBP values of the raw dataset

Overview	DBP	SBP
**Segment**	6025	6025
**Mean**	87	129
**STD**	12	17
**Min**	58	100
**Max**	123	206

Supporting Table 12: Statistics of reference DBP and SBP values of the pre-processed dataset

Overview	SBP	DBP
**Segment**	5143	5143
**Mean**	84	128
**STD**	9	14
**Min**	57	100
**Max**	100	179

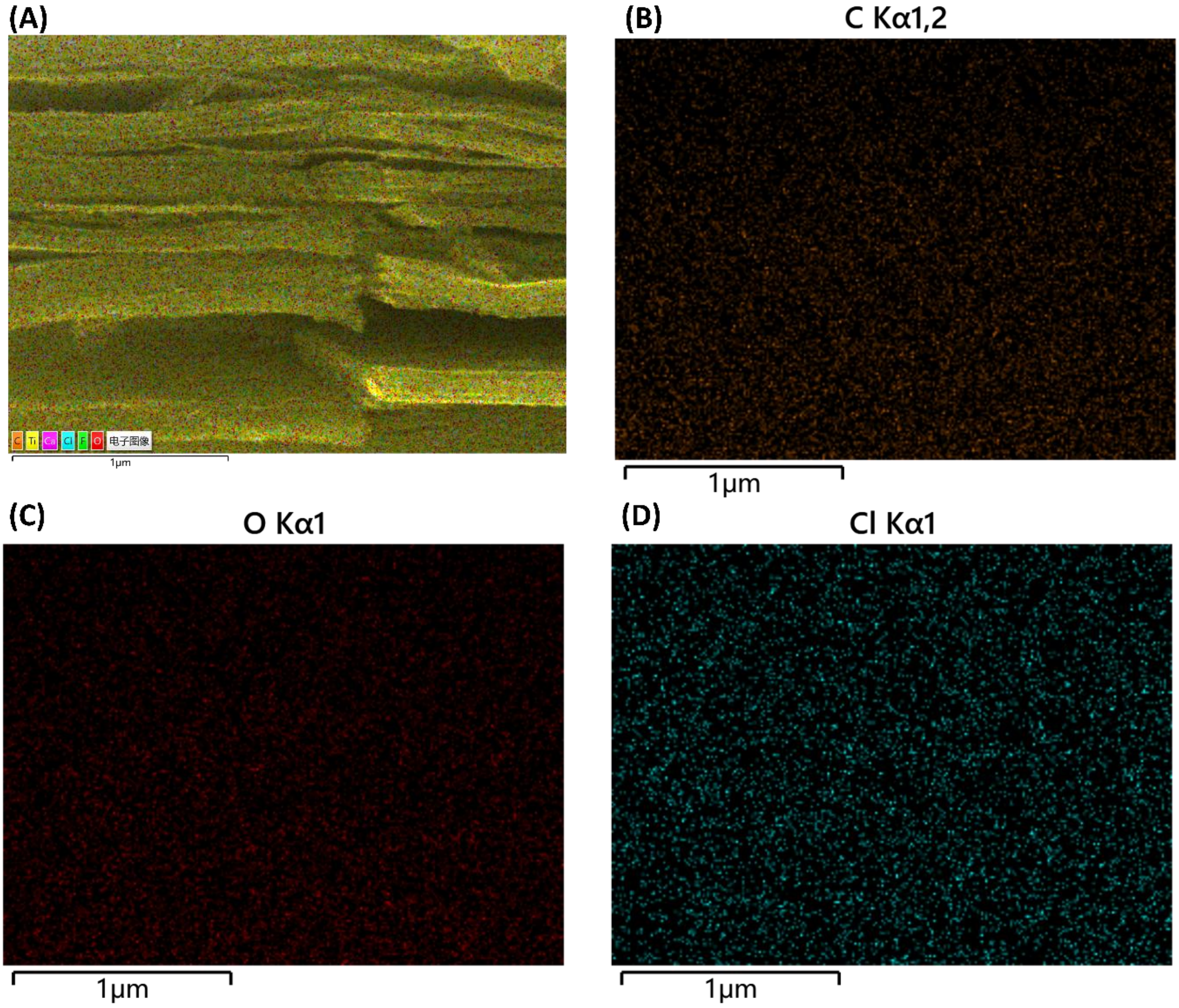

Supporting Figure 1: SEM-EDS photographs of ion-driven $Ti_3C_2T_x$. (A) SEM-EDS of ion-driven $Ti_3C_2T_x$ with combined elements at 1um scale. (B) SEM-EDS mapping of Carbon (C) in ion-driven $Ti_3C_2T_x$ at 1um scale. (C) SEM-EDS mapping of Oxygen (O) in ion-driven $Ti_3C_2T_x$ at 1um scale. (D) SEM-EDS mapping of Chlorine (Cl) in ion-driven $Ti_3C_2T_x$ at 1um scale.

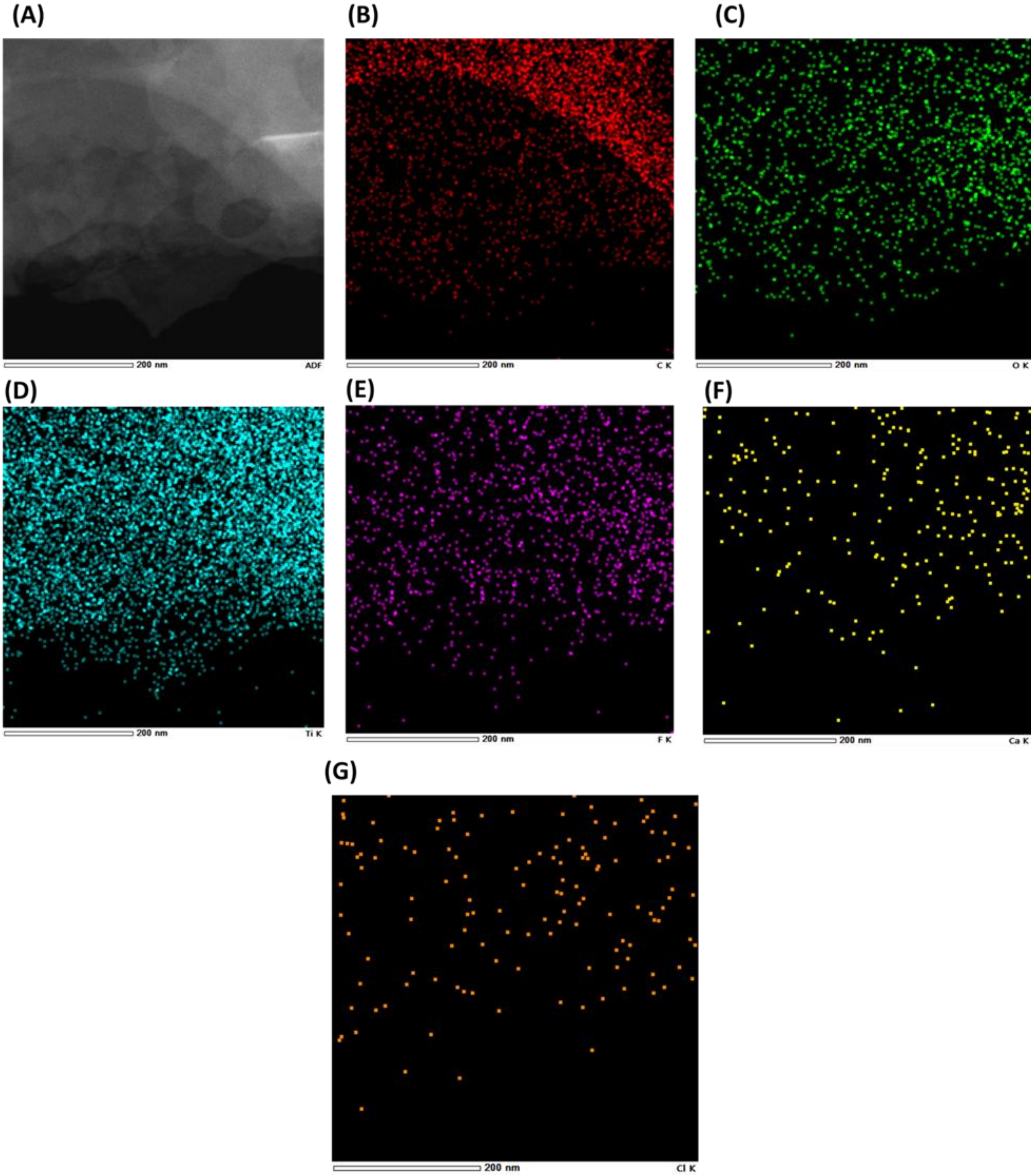

Supporting Figure 2: TEM-EDS photographs of Ion-driven $Ti_3C_2T_x$ at 200 nm, (A) TEM energy-dispersive spectroscopy (EDS) mapping of ion-driven $Ti_3C_2T_x$ nanosheets. (B) TEM energy-dispersive spectroscopy (EDS) mapping of Carbon (C) of ion-driven $Ti_3C_2T_x$ nanosheets. (C) TEM energy-dispersive spectroscopy (EDS) mapping of Oxygen (O) of ion-driven $Ti_3C_2T_x$ nanosheets. (D) TEM energy-dispersive spectroscopy (EDS) mapping of Titanium (Ti) of ion-driven $Ti_3C_2T_x$ nanosheets. (E) TEM energy-dispersive spectroscopy (EDS) mapping of Fluorine (F) of ion-driven $Ti_3C_2T_x$ nanosheets. (F) TEM energy-dispersive

spectroscopy (EDS) mapping of Calcium (Ca) of ion-driven $Ti_3C_2T_x$ nanosheets. (G) TEM energy-dispersive spectroscopy (EDS) mapping of Chlorine (Cl) of ion-driven $Ti_3C_2T_x$ nanosheets.

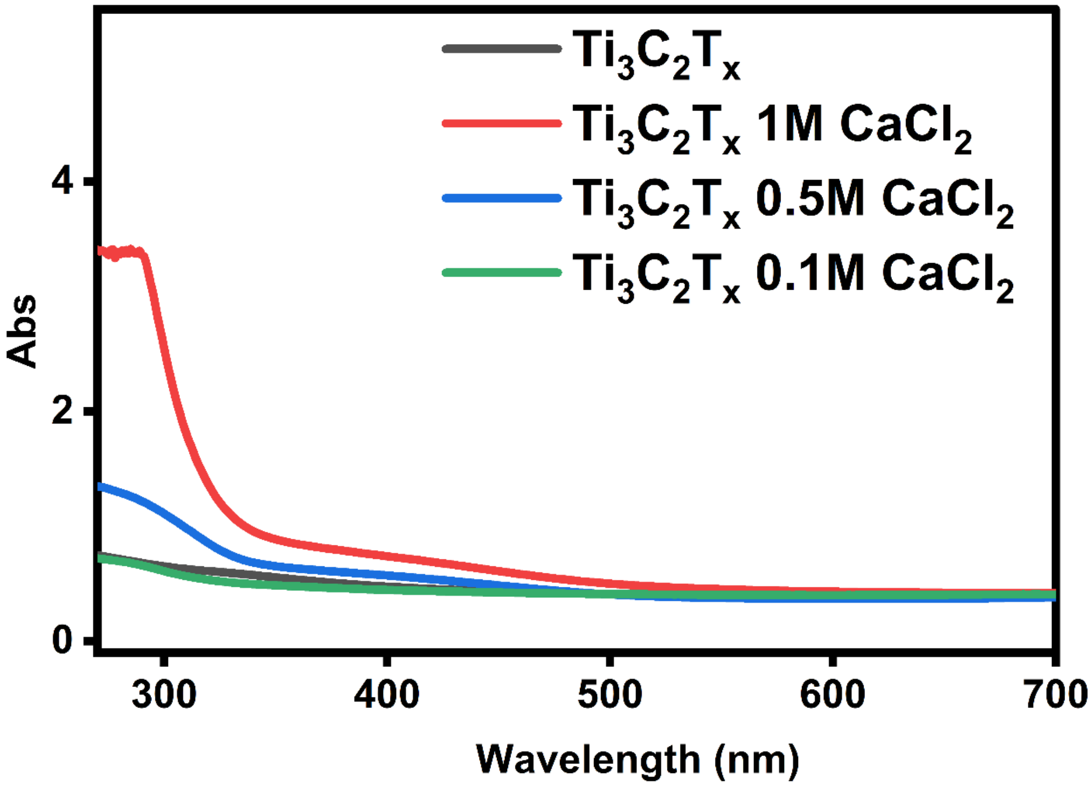

Supporting Figure 3: Solution dispersion study of $Ti_3C_2T_x$ with different concentrations (0.1M, 0.5M, 1M) of $CaCl_2$ using UV vis spectroscopy.

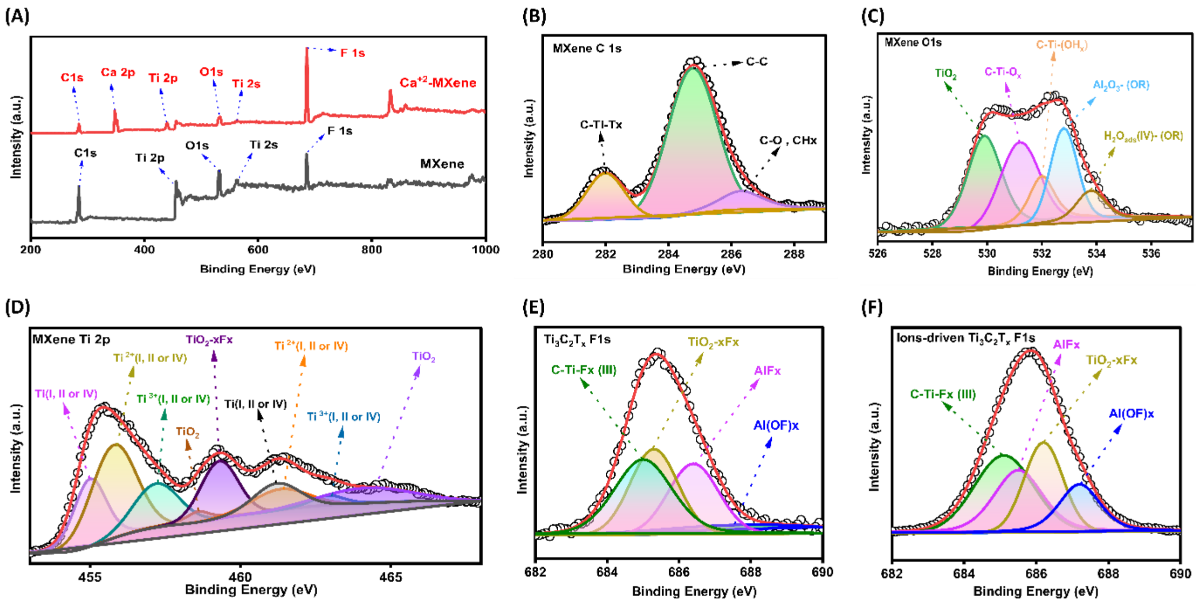

Supporting Figure 4: XPS Spectra of $Ti_3C_2T_x$ and ion-driven $Ti_3C_2T_x$. (A) XPS survey spectrum of $Ti_3C_2T_x$ and ion-driven $Ti_3C_2T_x$ (B) XPS deconvolution of the C1s peak of $Ti_3C_2T_x$ (C) XPS deconvolution of the O1s peak of $Ti_3C_2T_x$ (D) XPS deconvolution of the Ti2p peak of $Ti_3C_2T_x$ (E) XPS deconvolution of the F1s peak of $Ti_3C_2T_x$ (F) XPS deconvolution of the F1s peak of ion-driven $Ti_3C_2T_x$.

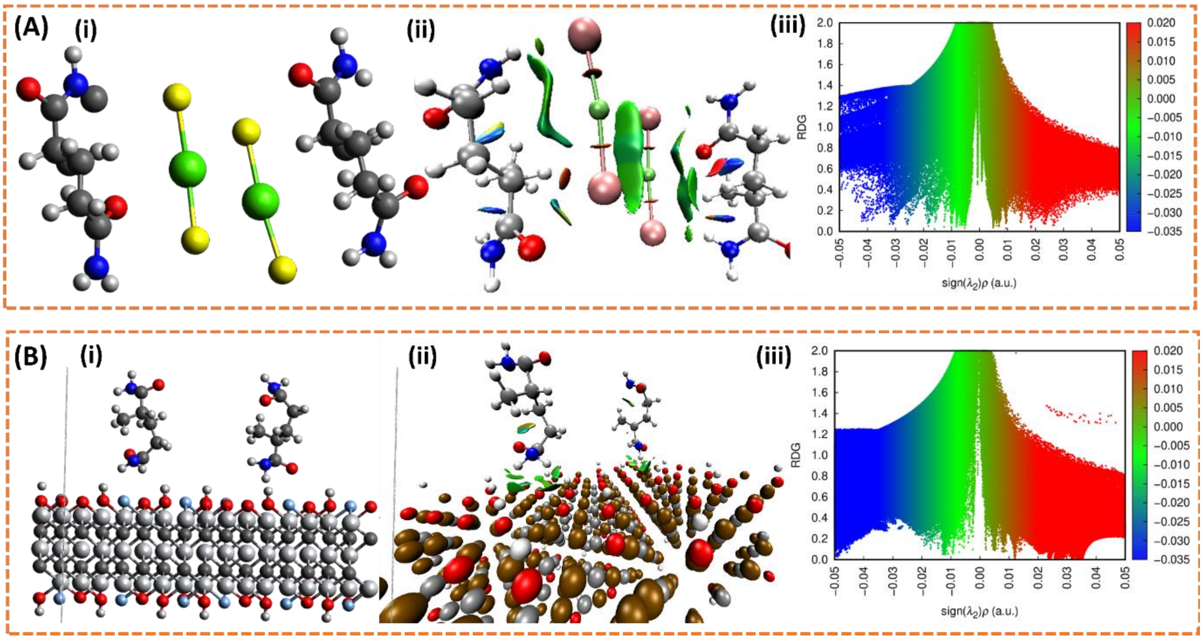

Supporting Figure 5: Non-Covalent Interactions (NCI) of $Ti_3C_2T_x$, PAM and $CaCl_2$ (A) Non-Covalent Interactions (NCI) of PAM and $CaCl_2$ (i) Molecular structure of PAM and $CaCl_2$, (ii) NCI visuals of PAM and $CaCl_2$, (iii) Reduced density gradient (RDG) of PAM and $CaCl_2$ (C) Non-Covalent Interactions (NCI) of $Ti_3C_2T_x$ and PAM (i) Molecular structure of $Ti_3C_2T_x$ and PAM, (ii) NCI visuals of $Ti_3C_2T_x$ and PAM, (iii) Reduced density gradient (RDG) of $Ti_3C_2T_x$ and PAM.

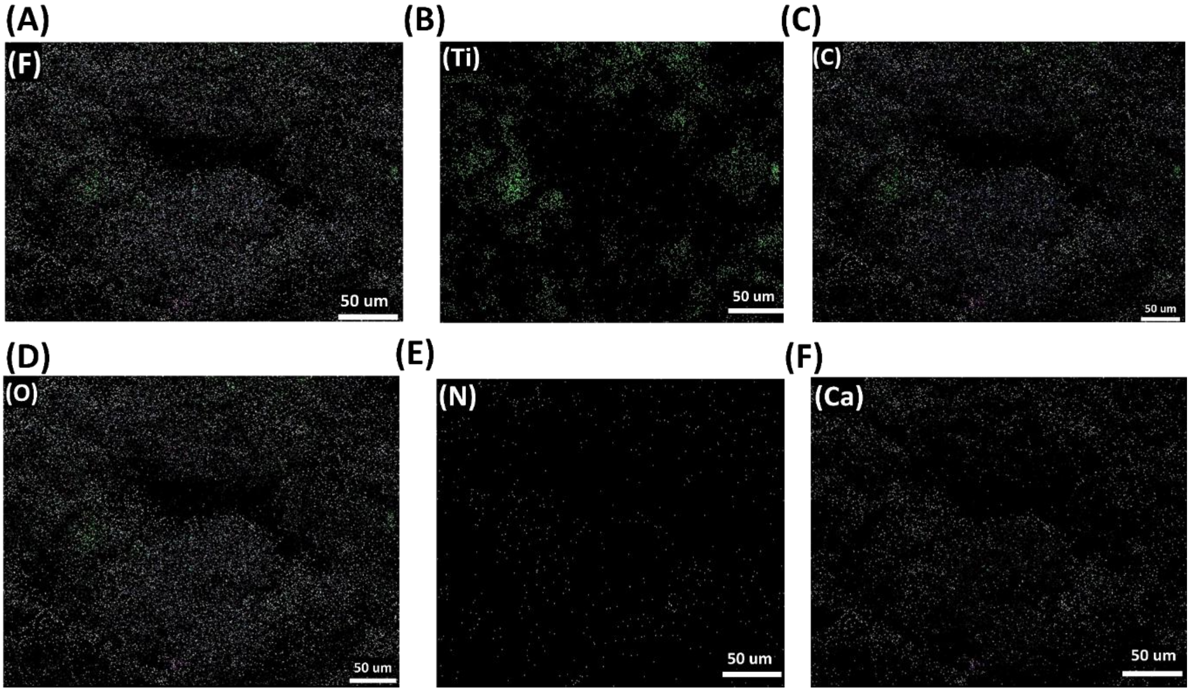

Supporting Figure 6: SEM-EDS photographs of Ion-driven $Ti_3C_2T_x$-PAM Hydrogel. (A) SEM- Energy-dispersive spectroscopy (EDS) mapping of Fluorine (F) of Ions-driven $Ti_3C_2T_x$-PAM Hydrogel. (B) SEM- Energy-dispersive spectroscopy (EDS) mapping of Titanium (Ti) of Ions-driven $Ti_3C_2T_x$-PAM Hydrogel. (C) SEM-Energy-dispersive spectroscopy (EDS) mapping of Carbon (C) of Ions-driven $Ti_3C_2T_x$-PAM hydrogel. (D) SEM-Energy-dispersive spectroscopy (EDS) mapping of Oxygen (O) of Ions-driven $Ti_3C_2T_x$-PAM hydrogel. (E) SEM- Energy-dispersive spectroscopy (EDS) mapping of Nitrogen (N) of Ions-driven $Ti_3C_2T_x$-PAM Hydrogel. (F) SEM- Energy-dispersive spectroscopy (EDS) mapping of Calcium (Ca) ions-driven $Ti_3C_2T_x$-PAM hydrogel.

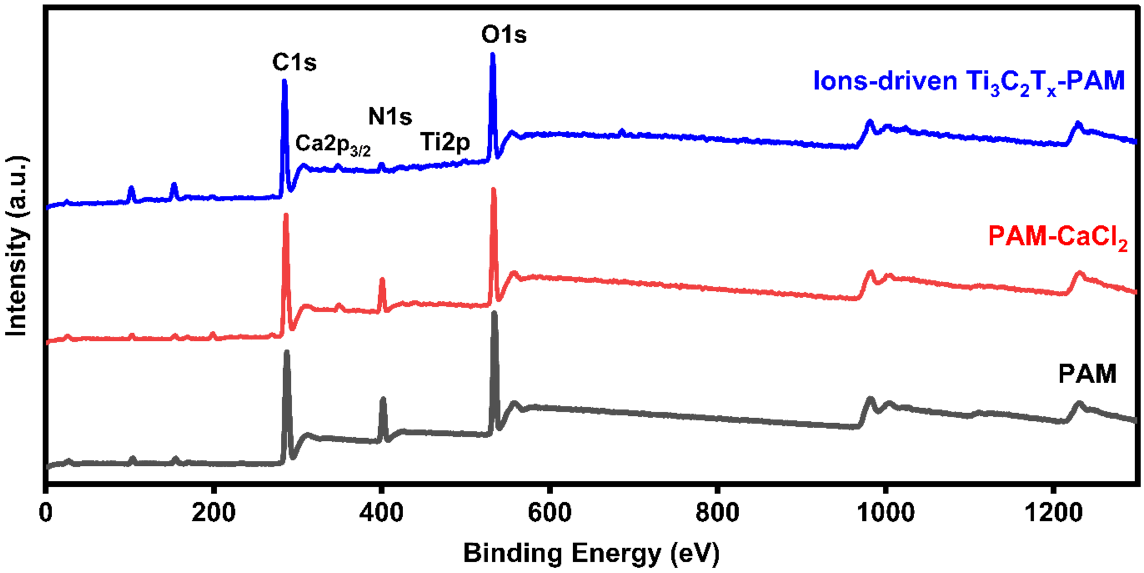

Supporting Figure 7: XPS Survey of PAM, PAM-$CaCl_2$, and Ion-driven $Ti_3C_2T_x$ -PAM hydrogels.

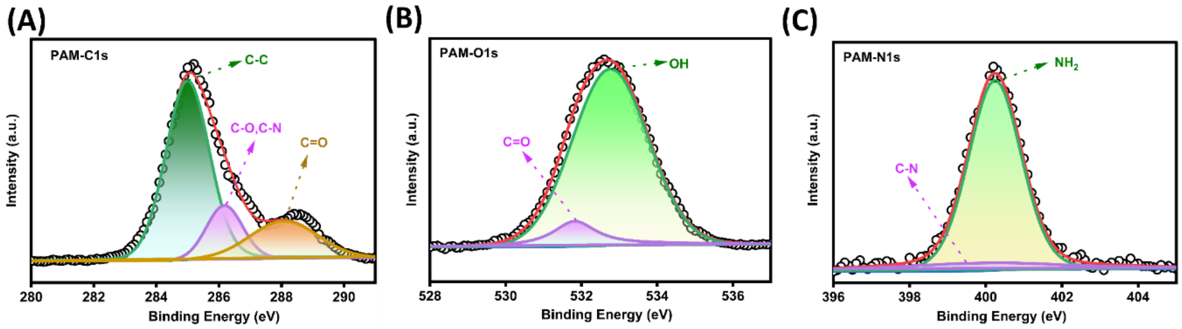

Supporting Figure 8: XPS Spectra of PAM hydrogels. (A) XPS deconvolution of the C1s peak of PAM. (B) XPS deconvolution of the O1s peak of PAM (C) XPS deconvolution of the N1s peak of PAM

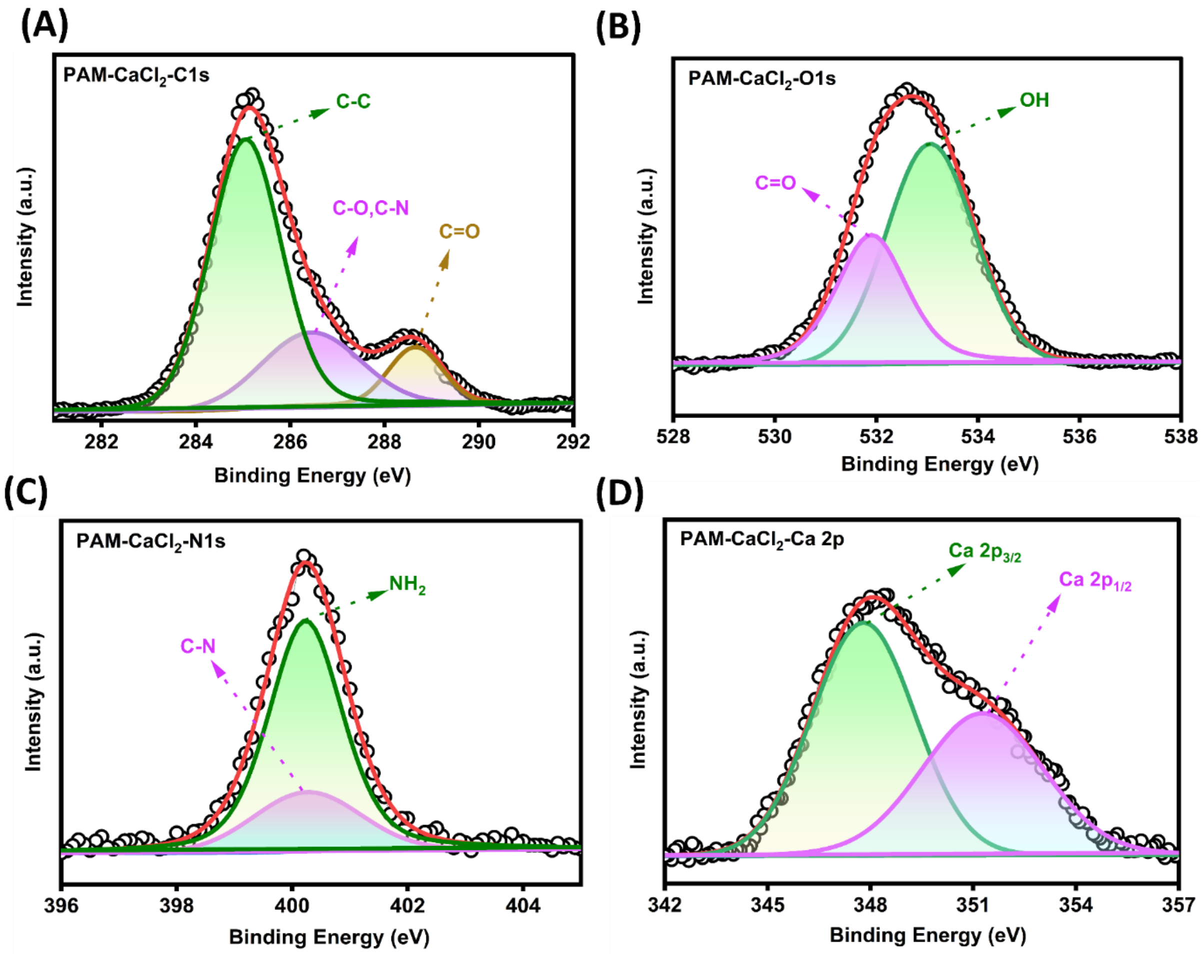

Supporting Figure 9: XPS Spectra of PAM-$CaCl_2$ hydrogels. (A) XPS deconvolution of the C1s peak of PAM-$CaCl_2$. (B) XPS deconvolution of the O1s peak of PAM-$CaCl_2$ (C) XPS deconvolution of the N1s peak of PAM-$CaCl_2$. (D) XPS deconvolution of the Ca 2p peak of PAM-$CaCl_2$.

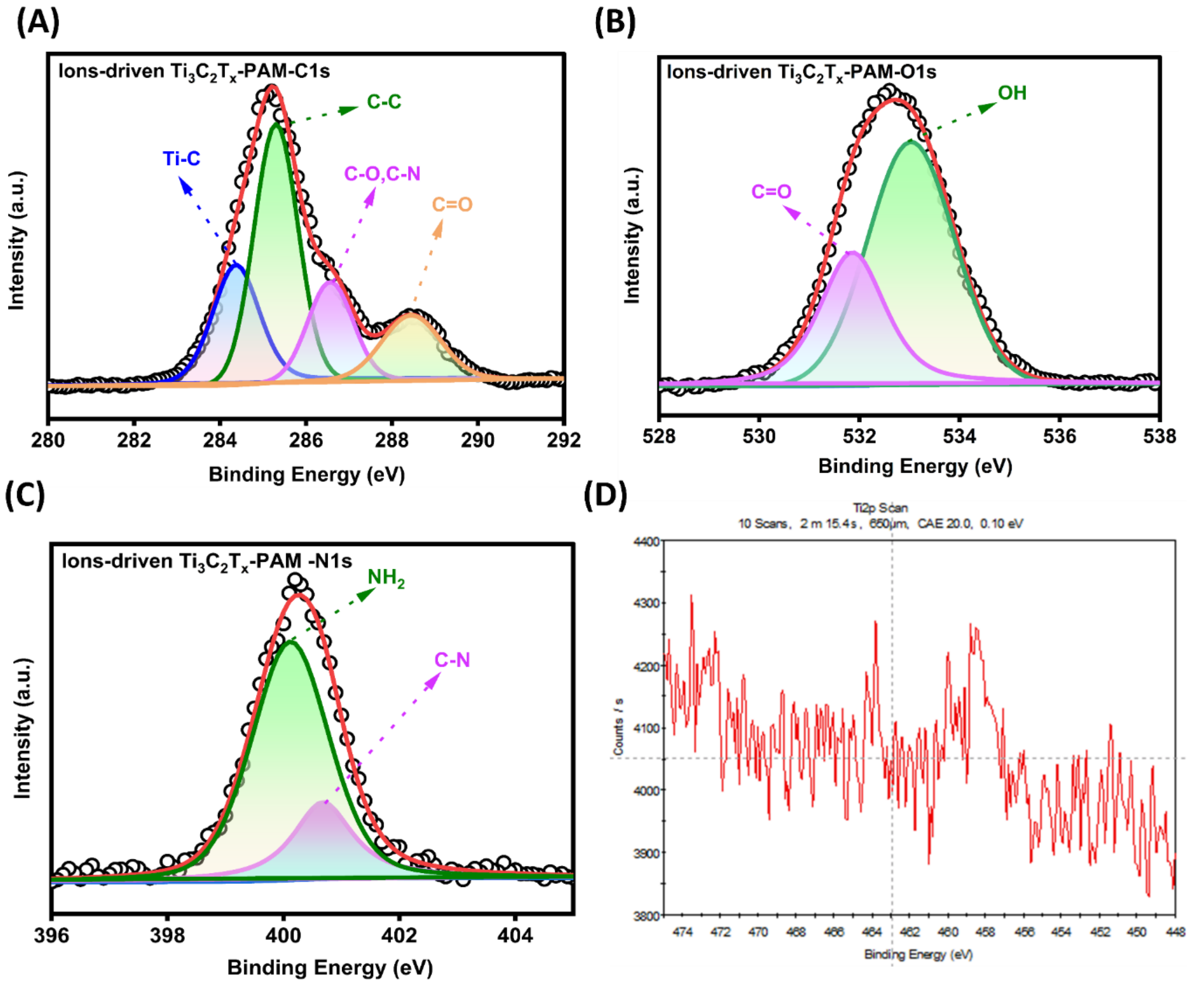

Supporting Figure 10: XPS Spectra of Ions-driven $Ti_3C_2T_x$-PAM hydrogels. (A) XPS deconvolution of the C1s peak of Ions-driven $Ti_3C_2T_x$-PAM hydrogel. (B) XPS deconvolution of the O1s peak of Ions-driven $Ti_3C_2T_x$-PAM hydrogel. (C) XPS deconvolution of the N1s peak of Ions-driven $Ti_3C_2T_x$-PAM hydrogel. (D) XPS Ti2p peak of Ions-driven $Ti_3C_2T_x$-PAM hydrogel.

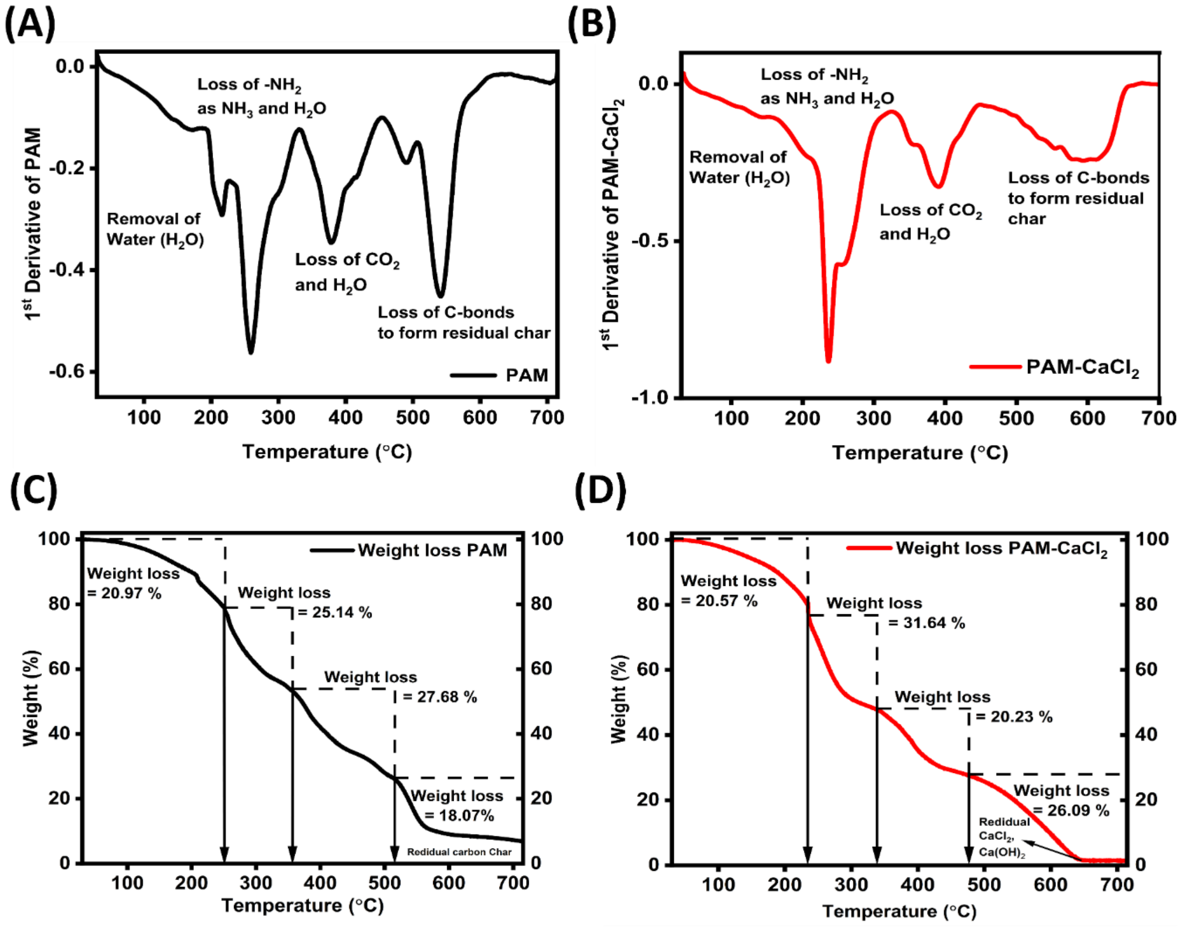

Supporting Figure 11: Thermal Characterization of PAM and PAM-$CaCl_2$ (A) Differential scanning calorimetry (DSC) graph of PAM hydrogel. (B) Differential scanning calorimetry (DSC) graph of PAM-$CaCl_2$ hydrogel (C). Thermogravimetric analysis (TGA) graph of PAM hydrogel. (D) Thermogravimetric analysis (TGA) graph of PAM-$CaCl_2$ hydrogel.

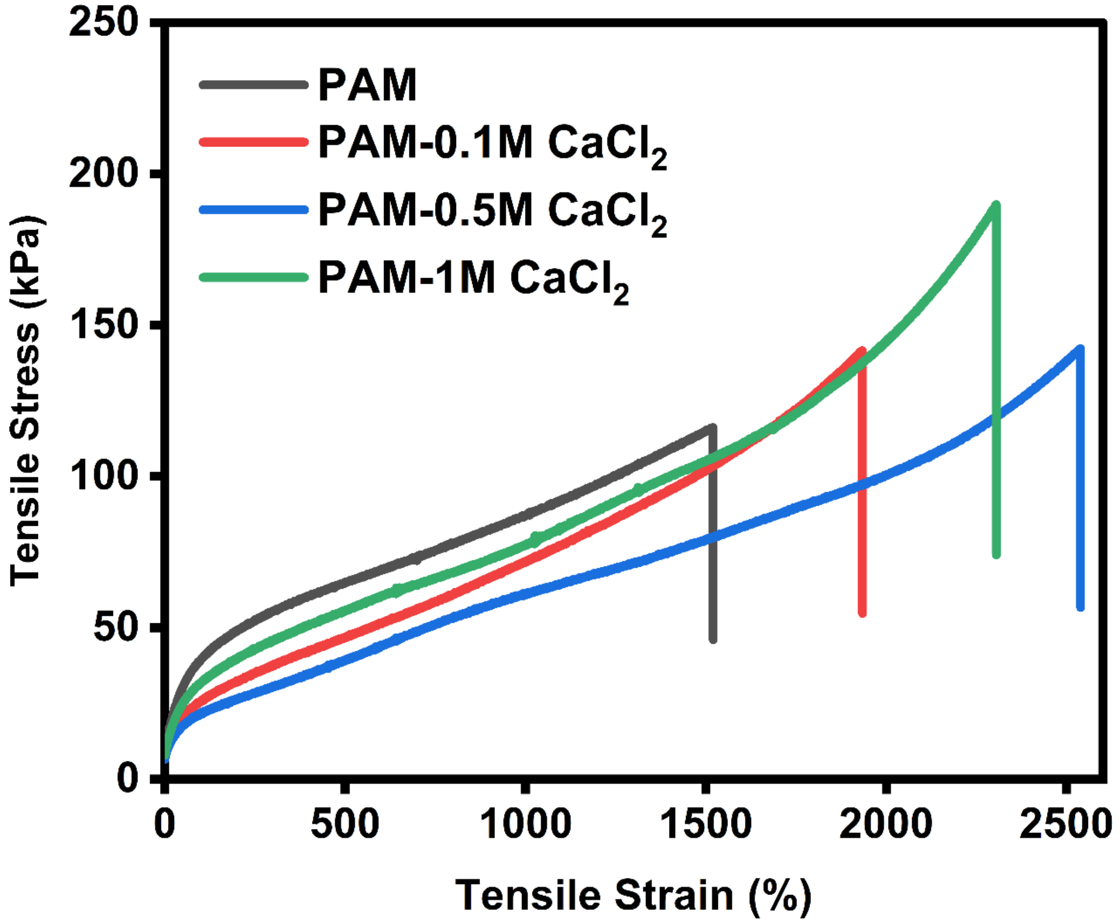

Supporting Figure 12: Tensile Testing of PAM, PAM 0.1M $CaCl_2$, PAM 0.5 M $CaCl_2$, PAM 1M $CaCl_2$

.

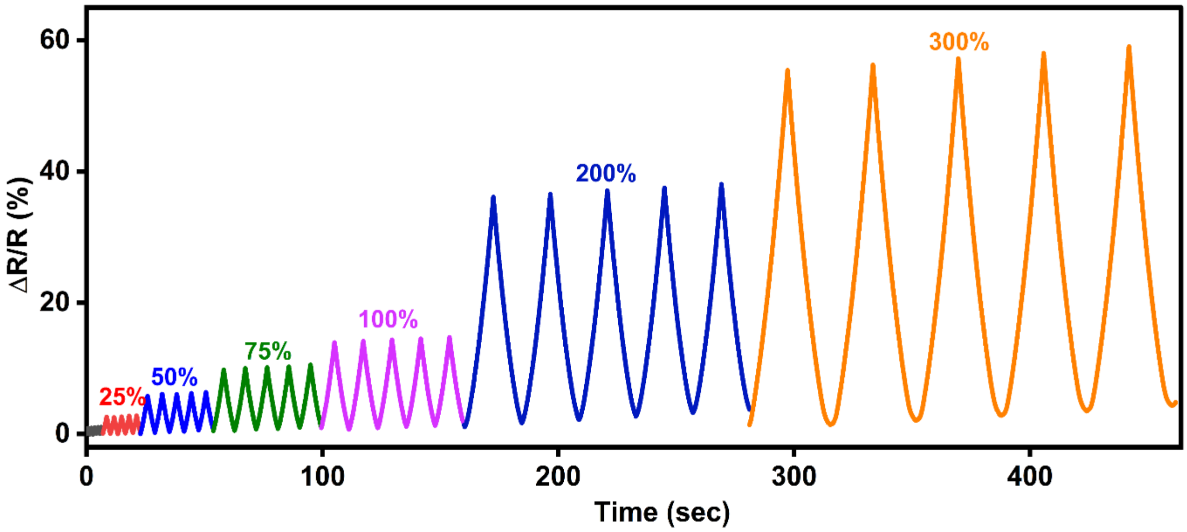

Supporting Figure 13: Relative resistance changes ($\Delta R/R_0$) in Ions-driven $Ti_3C_2T_x$-PAM hydrogel over a 10–300% strain range

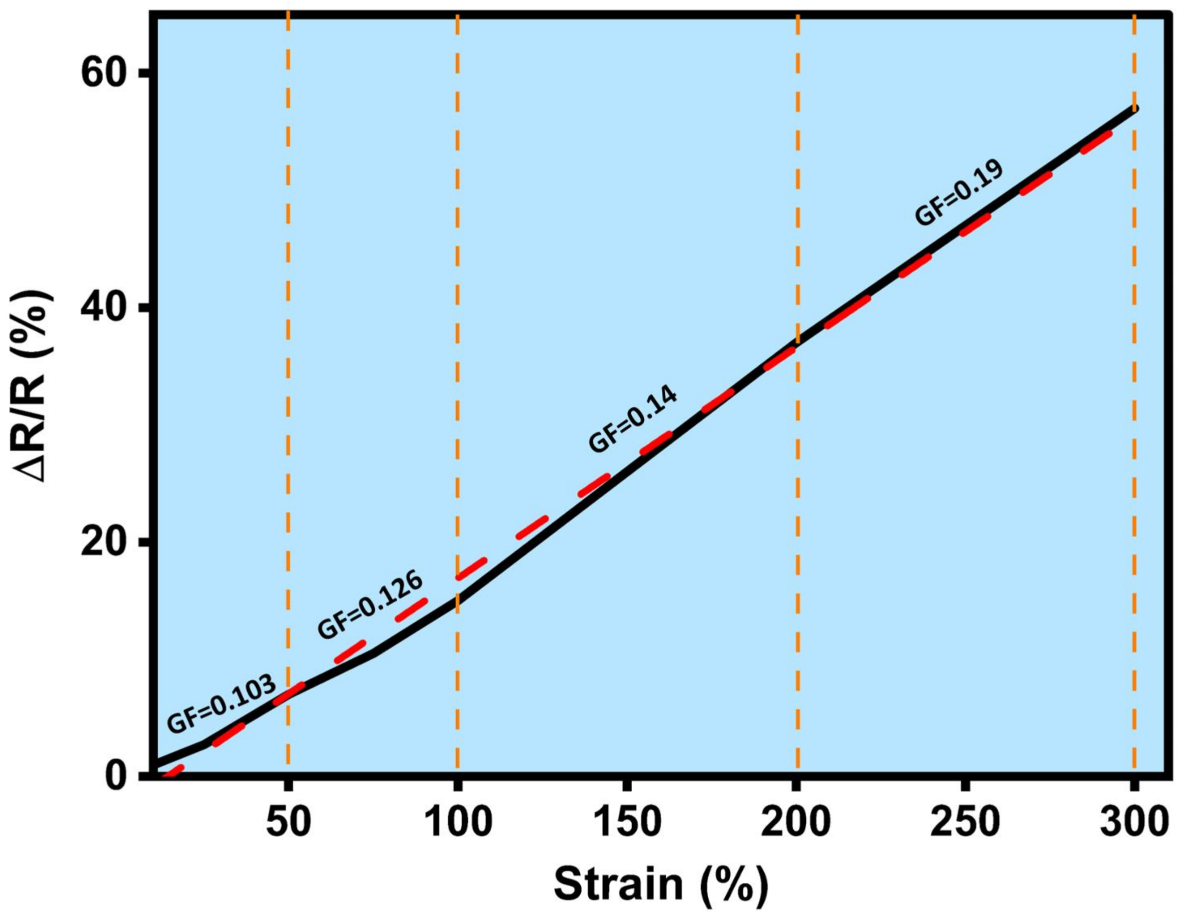

Supporting Figure 14: Gauge factors and relative resistance changes ($\Delta R/R_0$) in Ions-driven $Ti_3C_2T_x$-PAM hydrogel at 10 to 300% strains.

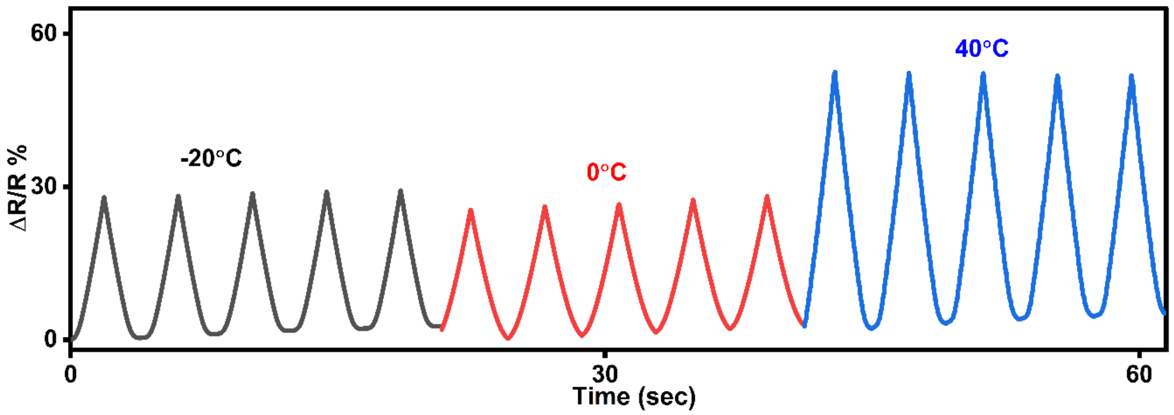

Supporting Figure 15: Relative resistance changes ($\Delta R/R_0$) in Ions-driven $Ti_3C_2T_x$-PAM hydrogel over a 100% strain range at -20°C, 0°C and 40 °C

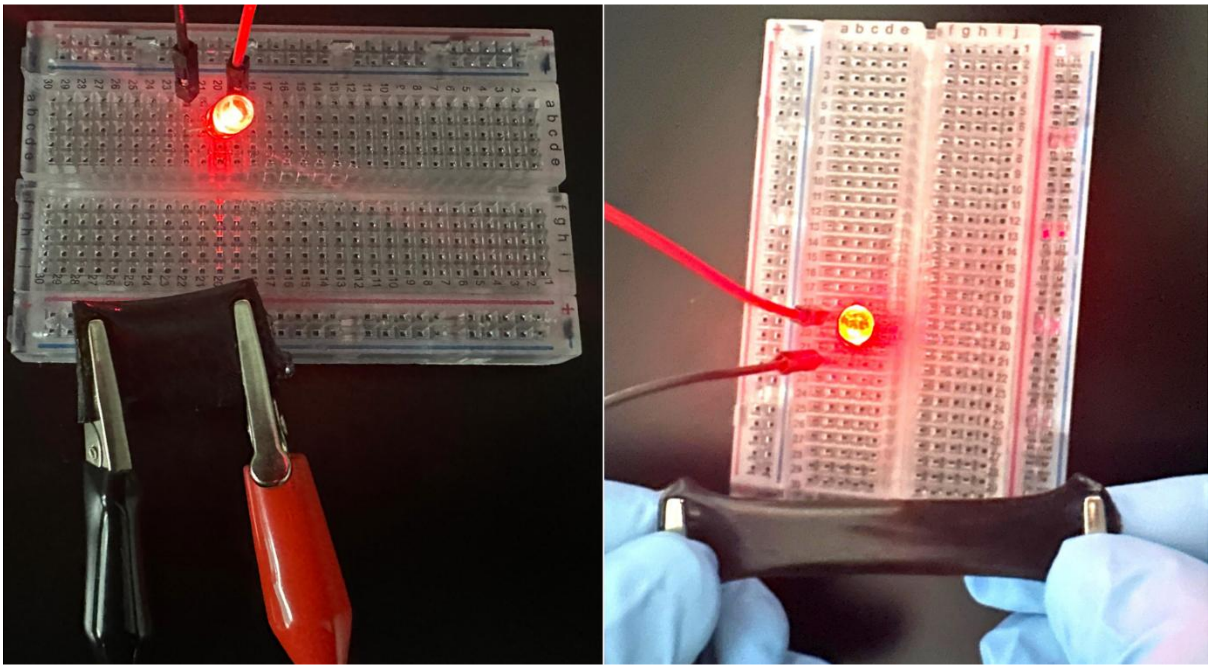

Supporting Figure 16: Visual demonstration of the conductivity of ion-driven $Ti_3C_2T_x$-PAM hydrogel under various strain percentages using an LED.

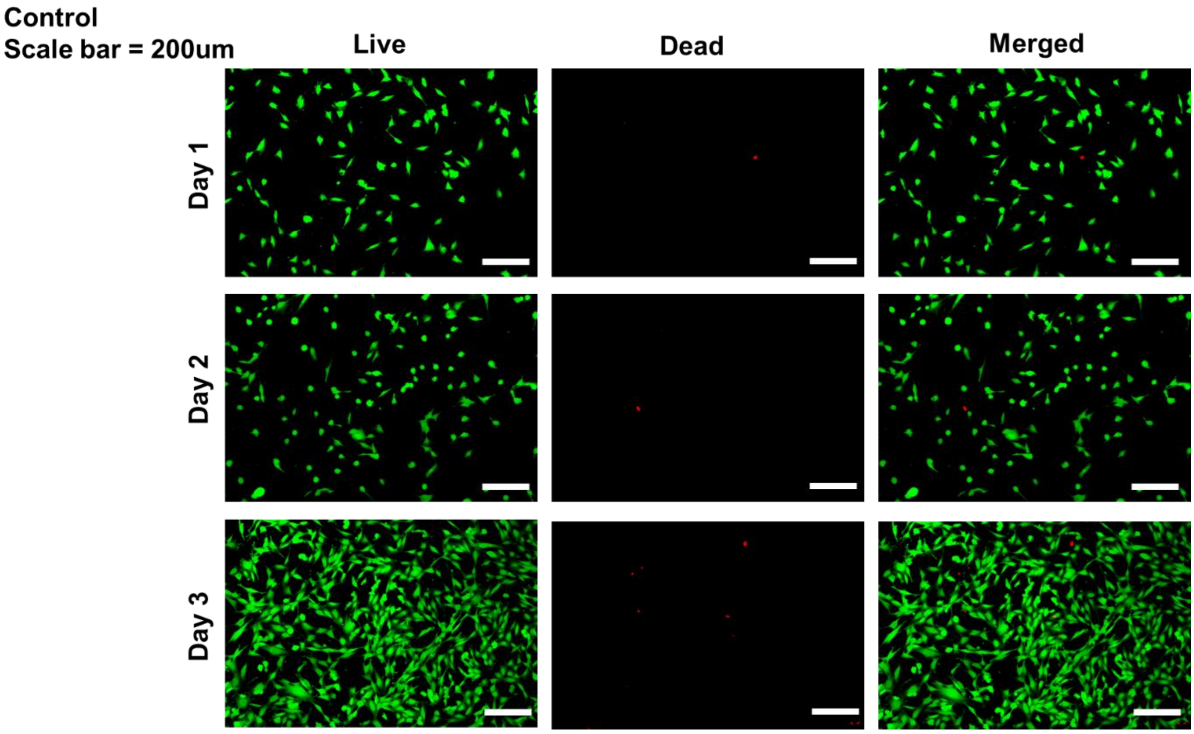

Supporting Figure 17: Fluorescence Cell Viability of Control of Ions-driven $Ti_3C_2T_x$−PAM hydrogel.

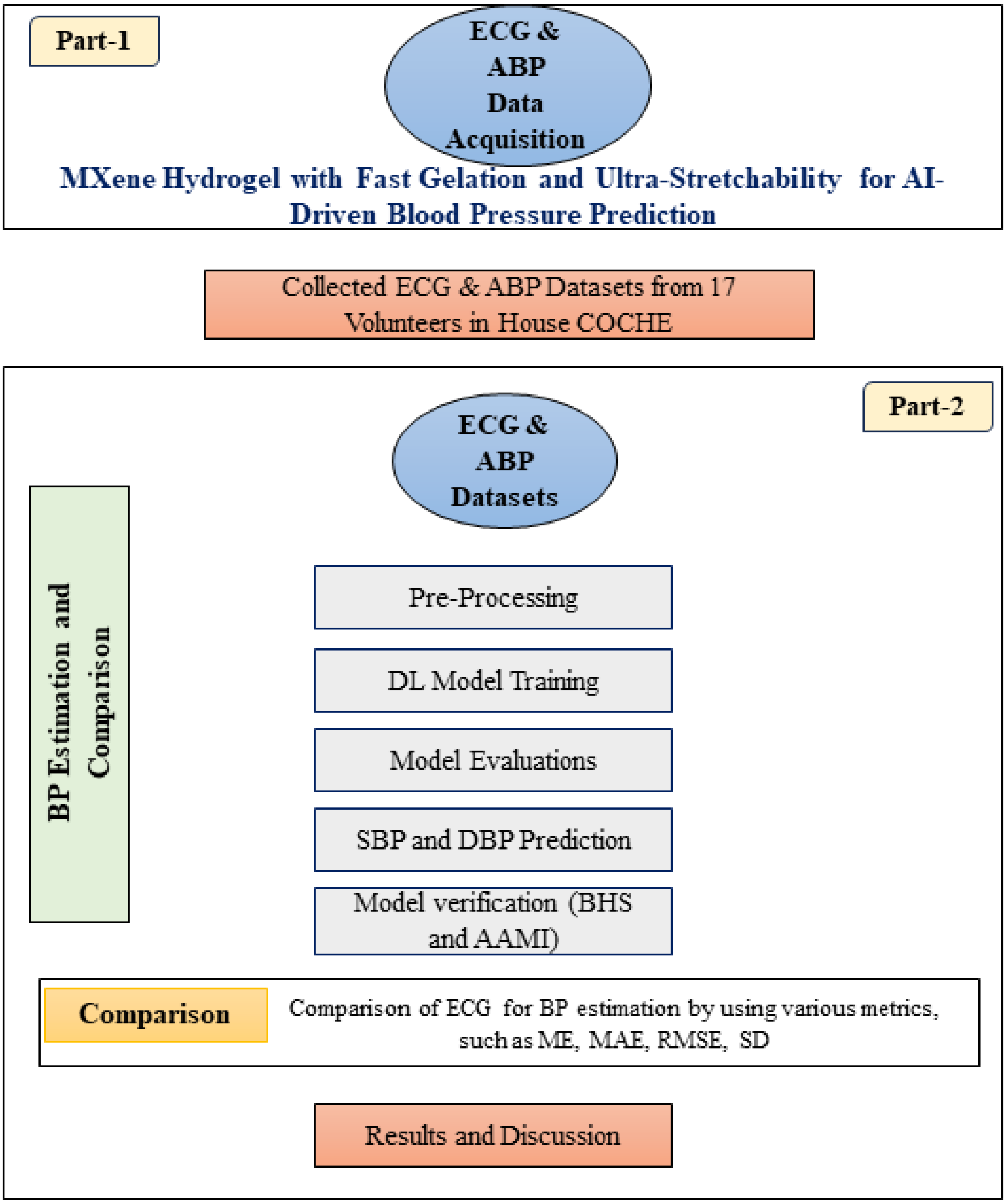

Supporting Figure 18: Flowchart of proposed AI-enabled PB Prediction

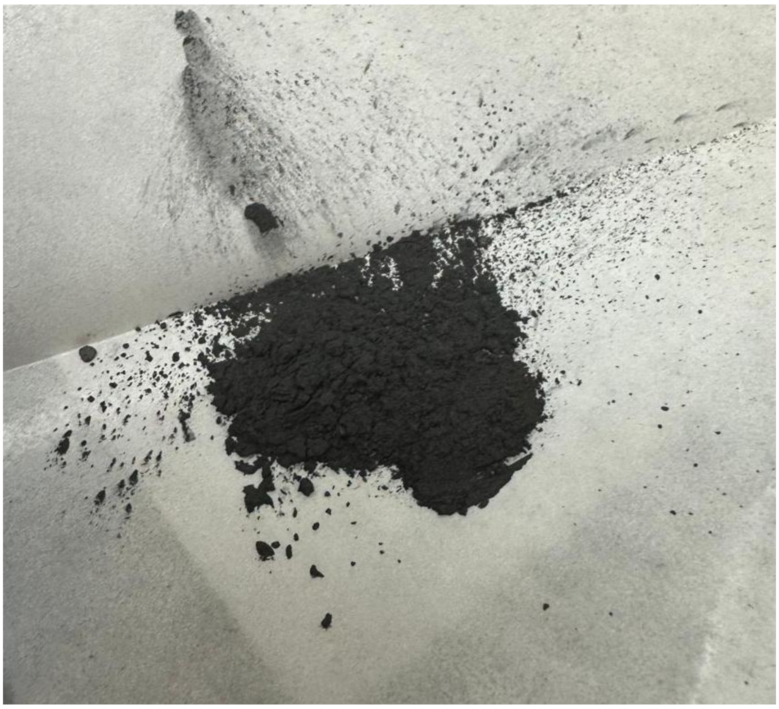

Supporting Figure 19: MXene ($Ti_3C_2T_x$) nanolayer powder

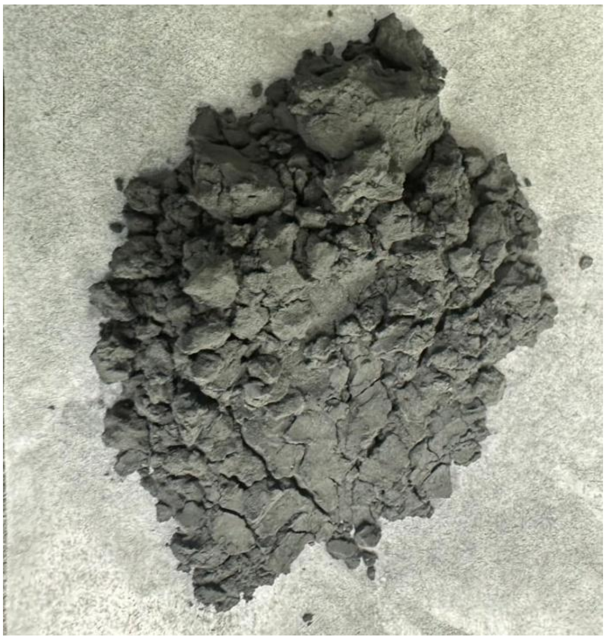

Supporting Figure 20: MAX (Ti_3AlC_2) powder

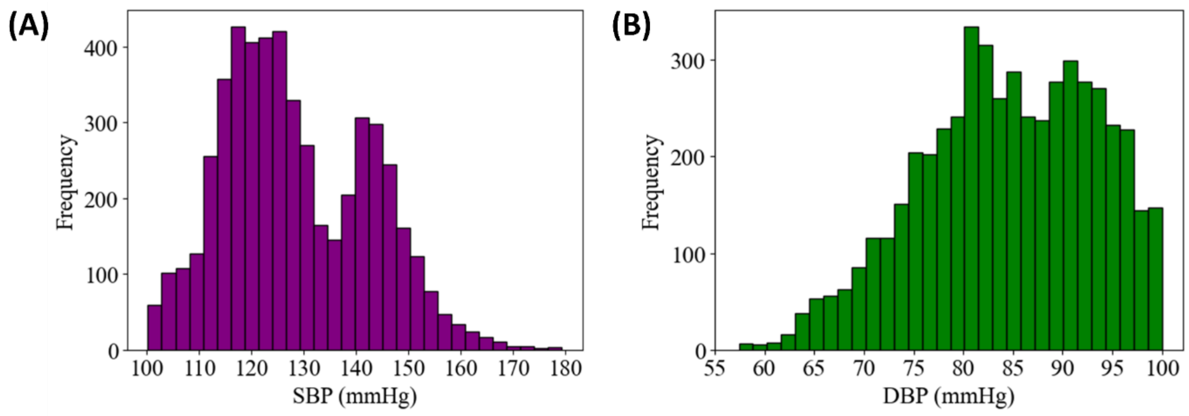

Supporting Figure 21: The arterial blood pressure bar plot of SBP (a) and DBP (b) of the pre-process dataset

References:

[1] X. Sun *et al.*, "Carbon nanotubes reinforced hydrogel as flexible strain sensor with high stretchability and mechanically toughness," *Chemical Engineering Journal,* vol. 382, p. 122832, 2020.

[2] Y. Wan, L. Zhang, T. Wu, C. Tang, H. Song, and Q. Cao, "High-performance and frost-resistance MXene co-ionic liquid conductive hydrogel printed by electrohydrodynamic for flexible strain sensor," *Journal of Colloid and Interface Science,* vol. 669, pp. 688-698, 2024.

[3] Y. Zhang, Y. Dai, F. Xia, and X. Zhang, "Gelatin/polyacrylamide ionic conductive hydrogel with skin temperature-triggered adhesion for human motion sensing and body heat harvesting," *Nano Energy,* vol. 104, p. 107977, 2022.

[4] J. Du *et al.*, "Application of adhesive controllable galactomannan hydrogel initiated by aluminum ions at room temperature in flexible sensors," *Reactive and Functional Polymers,* vol. 193, p. 105738, 2023.

[5] J. Yang *et al.*, "Self-powered, frost-resistant, and antimicrobial hydrogel with high open-circuit voltage for flexible device energy supply and stimuli feedback," *Chemical Engineering Journal,* vol. 496, p. 153933, 2024.

[6] X. Li, X. Li, M. Yan, and Q. Wang, "Chitosan-based transparent and conductive hydrogel with highly stretchable, adhesive and self-healing as skin-like sensor," *International Journal of Biological Macromolecules,* vol. 242, p. 124746, 2023.

[7] N. Li *et al.*, "Ultrastretchable, Self-Adhesive and conductive MXene nanocomposite hydrogel for body-surface temperature distinguishing and electrophysiological signal monitoring," *Chemical Engineering Journal,* vol. 483, p. 149303, 2024.

[8] K. Li *et al.*, "An ultrasound-induced MXene doped PAM–SA super-tough hydrogel," *Journal of Materials Chemistry C,* vol. 11, no. 5, pp. 1908-1918, 2023.

[9] F. Zhang *et al.*, "Ionic organohydrogel with long-term environmental stability and multifunctionality based on PAM and sodium alginate," *Chemical Engineering Journal,* vol. 485, p. 149810, 2024.

[10] M. Qin *et al.*, "Preparation of PAA/PAM/MXene/TA hydrogel with antioxidant, healable ability as strain sensor," *Colloids and Surfaces B: Biointerfaces,* vol. 214, p. 112482, 2022.

[11] W. Zhang, X. Zhang, W. Zhao, and X. Wang, "High-sensitivity composite dual-network hydrogel strain sensor and its application in intelligent recognition and motion monitoring," *ACS Applied Polymer Materials,* vol. 5, no. 4, pp. 2628-2638, 2023.

[12] Q. Wang *et al.*, "Modified Ti3C2TX (MXene) nanosheet-catalyzed self-assembled, anti-aggregated, ultra-stretchable, conductive hydrogels for wearable bioelectronics," *Chemical Engineering Journal,* vol. 401, p. 126129, 2020.

[13] W. Zhao, J. Jiang, W. Chen, Y. He, T. Lin, and L. Zhao, "Radiation synthesis of rapidly self-healing, durable, and flexible poly (ionic liquid)/MXene gels with anti-freezing property for multi-functional strain sensors," *Chemical Engineering Journal,* vol. 468, p. 143660, 2023.

[14] X. Wang *et al.*, "Interface interaction-mediated design of tough and conductive MXene-composited polymer hydrogel with high stretchability and low hysteresis for high-performance multiple sensing," *Science China Materials,* vol. 66, no. 1, pp. 272-283, 2023.

[15] J. Zeng, X. Jing, L. Lin, G. Wang, Y. Zhang, and P. Feng, "Smart sensing hydrogel

actuators conferred by MXene gradient arrangement," *Journal of Colloid and Interface Science,* vol. 677, pp. 816-826, 2025.
[16] X. Zhang *et al.*, "Self-Adhesive ILn@ MXene multifunctional hydrogel with excellent dispersibility for human-machine interaction, capacitor, antibacterial and detecting various physiological electrical signals in humans and animals," *Nano Energy,* vol. 133, p. 110484, 2025.
[17] H. Yin *et al.*, "MXene-based conductive hydrogels with toughness and self-healing enhancement by metal coordination for flexible electronic devices," *Materials Today Physics,* vol. 47, p. 101537, 2024.
[18] Y. Liu *et al.*, "Highly Stretchable, Low-Hysteresis, and Adhesive TA@ MXene-Composited Organohydrogels for Durable Wearable Sensors," *Advanced Functional Materials*, vol. 34, no. 30, p. 2315813, 2024.
[19] L. Lin *et al.*, "Fabrication of high-toughness, puncture-resistant hydrogels based on nanoengineered MXene for flexible electronics," *ACS Applied Polymer Materials,* vol. 6, no. 18, pp. 11497-11507, 2024.
[20] J. Zou, X. Jing, S. Li, P. Feng, Y. Chen, and Y. Liu, "MXene crosslinked hydrogels with low hysteresis conferred by sliding tangle island strategy," *Small,* vol. 20, no. 35, p. 2401622, 2024.
